# Supplementary material for: Efficacy of maternal B12 supplementation in vegetarian women for improving infant neurodevelopment: protocol for the MATCOBIND multicentre, double-blind, randomised controlled trial
Source: BMJ Open. 2020 May 25;10(5):e034987. doi: 10.1136/bmjopen-2019-034987 (PMC7252986; doi:10.1136/bmjopen-2019-034987)
Supplement: Supplementary data [file bmjopen-2019-034987supp001.pdf]

Supplementary Material: Table 1. Visit schedule

**Study Plan**	**Initial Contact (<12 weeks gestation)**		**M1**	**M2-M9**	**M10**	**At Birth**	**I1**	**I2-I6**	**I7**
**Schedule**	**Screening**	**Recruitment**	**Antenatal Follow-up**	**Antenatal Follow-up (monthly until second trimester, fortnightly in the third trimester**	**Antenatal Follow-up (weekly after 36 weeks until delivery)**		**Infant 7-14 day**	**Infant follow-up: At months- 1 (± 1 wk), 2 (± 2 wks), 3 (± 2 wks), 4 (± 2 wks) & 6 (± 2 wks)**	**Infant follow-up Age 9 months ± 14 days;**
First Screening Visit	X								
Maternal Inclusion/Exclusion criteria	X	X							
Informed consent of the participant		X							
Questionnaire Administration		X							
Randomization		X							
Supplementation		X	X	X	X	X	X	X	
Blood Sampling (1st trimester)		X							
Home Visit (if required)			X	X	X	X	X	X	X
Monitoring of the Compliance			X	X	X	X	X	X	X
Adverse events			X	X	X	X	X	X	X
Blood Sampling (3rd trimester, >26 weeks gestation)				X					
Mother Food Frequency Questionnaire (>26 weeks gestation)				X					
Infant Anthropometry (Weight/length/Head Circumference)						X	X	X	X
Monitoring for neonatal morbidity*						X	X	X	X
Application of Exit Criteria (specified earlier)			X	X	X	X	X	X	X
Metabolic Screening of infant							X		
Infant morbidity							X	X	X
Infant blood sampling									X
Complementary feeding 72 hr. Dietary Recall									X
DASII Scale									X

HOME Inventory									X
