# Supplementary material for: Efficacy of maternal B12 supplementation in vegetarian women for improving infant neurodevelopment: protocol for the MATCOBIND multicentre, double-blind, randomised controlled trial
Source: BMJ Open. 2020 May 25;10(5):e034987. doi: 10.1136/bmjopen-2019-034987 (PMC7252986; doi:10.1136/bmjopen-2019-034987)
Supplement: Author's manuscript [file bmjopen-2019-034987.draft_revisions.pdf]

BMJ Open is committed to open peer review. As part of this commitment we make the peer review history of every article we publish publicly available.

When an article is published we post the peer reviewers' comments and the authors' responses online. We also post the versions of the paper that were used during peer review. These are the versions that the peer review comments apply to.

The versions of the paper that follow are the versions that were submitted during the peer review process. They are not the versions of record or the final published versions. They should not be cited or distributed as the published version of this manuscript.

BMJ Open is an open access journal and the full, final, typeset and author-corrected version of record of the manuscript is available on our site with no access controls, subscription charges or pay-per-view fees (http://bmjopen.bmj.com).

If you have any questions on BMJ Open's open peer review process please email info.bmjopen@bmj.com

Efficacy of maternal Vitamin B-12 supplementation in improving infant neurodevelopment: Protocol for the MATCOBIND multicentre, double-blind, randomised controlled trial

Journal:	*BMJ Open*
Manuscript ID	bmjopen-2019-034987
Article Type:	Protocol
Date Submitted by the Author:	20-Oct-2019
Complete List of Authors:	Nagpal, Jitender; Sitaram Bhartia Institute of Science and Research, Pediatrics<br>Mathur, Manu; Public Health Foundation of India,<br>Rawat , Swapnil ; Sitaram Bhartia Institute of Science and Research, Pediatrics<br>Nagrath, Deepti; Public Health Foundation of India,<br>Lee, Charlotte; University College London, Institute of Child Health<br>Singhal, Atul ; University College London Institute of Child Health<br>Heys, Michelle; University College London Institute of Child Health, University College London Institute of Child Health<br>Cortina Borja, Mario; UCL, Institute of Child Health<br>Augustin, Katrin ; University College London, Institute of Child Health<br>Gautam , Jageshwor<br>Pant , Rajendra<br>Lakhanpaul, Monica; University College London, Institute of Child Health
Keywords:	Maternal medicine < OBSTETRICS, PAEDIATRICS, PUBLIC HEALTH

SCHOLARONE™
Manuscripts

BMJ

I, the Submitting Author has the right to grant and does grant on behalf of all authors of the Work (as defined in the below author licence), an exclusive licence and/or a non-exclusive licence for contributions from authors who are: i) UK Crown employees; ii) where BMJ has agreed a CC-BY licence shall apply, and/or iii) in accordance with the terms applicable for US Federal Government officers or employees acting as part of their official duties; on a worldwide, perpetual, irrevocable, royalty-free basis to BMJ Publishing Group Ltd ("BMJ") its licensees and where the relevant Journal is co-owned by BMJ to the co-owners of the Journal, to publish the Work in this journal and any other BMJ products and to exploit all rights, as set out in our licence.

The Submitting Author accepts and understands that any supply made under these terms is made by BMJ to the Submitting Author unless you are acting as an employee on behalf of your employer or a postgraduate student of an affiliated institution which is paying any applicable article publishing charge ("APC") for Open Access articles. Where the Submitting Author wishes to make the Work available on an Open Access basis (and intends to pay the relevant APC), the terms of reuse of such Open Access shall be governed by a Creative Commons licence – details of these licences and which Creative Commons licence will apply to this Work are set out in our licence referred to above.

Other than as permitted in any relevant BMJ Author's Self Archiving Policies, I confirm this Work has not been accepted for publication elsewhere, is not being considered for publication elsewhere and does not duplicate material already published. I confirm all authors consent to publication of this Work and authorise the granting of this licence.

For peer review only

TITLE PAGE

Title

Efficacy of maternal Vitamin B-12 supplementation in improving infant neurodevelopment: Protocol for the MATCOBIND multicentre, double-blind, randomised controlled trial

Running Head: Maternal Vit-B12 and Infant Neurodevelopment Protocol

For peer review only

Authors

Jitender Nagpal[a], Manu Raj Mathur[b], Swapnil Rawat[a], Deepti Nagrath[b], Charlotte Lee[c], Atul Singhal[c], Michelle Heys[c], Mario Cortina-Borja[c], Katrin Augustin[c],Jageshwor Gautam[d], Rajendra Pant[d], Monica Lakhanpaul[c*]

a. *Sitaram Bhartia Institute of Science and Research (SBISR), B16, Qutab Institutional Area, New Delhi, 110016, India.* jitendernagpal@gmail.com; swapnil.rawat@gmail.com
b. *Public Health Foundation of India (PHFI)* Plot Number 47, Sector 44, Institutional Area, Gurugram – 122002, India. manu.mathur@phfi.org ; deepti.nagrath@phfi.org
c. *UCL Great Ormond Street Institute of Child Health, University College London, 30 Guildford Street, London, WC1N 1EH, United Kingdom.* a.singhal@ucl.ac.uk; m.heys@ucl.ac.uk; m.cortina@ucl.ac.uk; k.augustin@ucl.ac.uk; c.lee.12@ucl.ac.uk; m.lakhanpaul@ucl.ac.uk .
d. *Paropakar Maternity and Womens' Hospital (PMWH), Thapathali, Kathmandu, Nepal.* drjgautam48@gmail.com; rajendrapant8@gmail.com .

*Corresponding Author

Professor Monica Lakhanpaul; UCL Great Ormond Street Institute of Child Health, University College London, 30 Guildford Street, London, WC1N 1EH, United Kingdom; m.lakhanpaul@ucl.ac.uk .

Word Count

Abstract: 250

Main text: 3930

Tables: 2

Figures: 1

For peer review only - http://bmjopen.bmj.com/site/about/guidelines.xhtml

Key Words- Maternal Health, Vitamin B-12, Infant Neurodevelopment

ABSTRACT

Introduction: Vitamin B-12 deficiency is widely prevalent across many low- and middle-income countries especially where the diet is low in animal sources of food. While many observational studies document the relationship between B-12 deficiency in pregnancy and infant cognitive functions including memory, language and motor skills, evidence from clinical trials documenting preventability/reversibility on supplementation is sparse and inconclusive.

Methods and Analysis: 720 pregnant women in the first trimester will be recruited in this multicentric double blind randomised control trial from two sites (360 each). Enrolled participants will be randomised into two groups to receive either 250 mcg methylcobalamin or 50 mcg (quasi control dose) as oral supplements from enrolment to 6 months post-partum while ensuring blinding and allocation concealment. All mothers and their infants will continue to receive standard care at their respective institutes. The infants' neurodevelopment at 9 months of age will be compared between the two groups as the primary outcome. Biochemical markers of B-12 status will be assessed for the mother at the first trimester and third trimester while the infants' B-12 status will be assessed at 9 months of age as secondary outcomes of efficacy.

Ethical Considerations and Dissemination: The Study Protocol was approved by the Institutional Ethics Committee (IEC) at SBISR, PHFI, PMWH, NHRC and UCL Research Ethics Committee. The study results will be disseminated globally with academic and health professionals via conferences, presentations and publications. Researchers at each study site will share emerging results with participants during their follow-up visits.

For peer review only - http://bmjopen.bmj.com/site/about/guidelines.xhtml

ARTICLE SUMMARY

Study strengths

- First large-scale, multi-centre randomised controlled, double-blind trial to determine efficacy of vitamin B-12 supplementation up to 6 months post-partum in improving neurodevelopmental outcomes in infancy.
- Study design benefits from preceding qualitative patient and public involvement (PPI), and nested qualitative sub-study will ensure participant feedback and process evaluation to optimise trial conduct.
- The study is being conducted across two diverse socio-demographic populations, to increase generalisability of study findings.

Study limitations

- Different regulatory structures in two different countries.
- Normal/Routine care delivered in the two study sites differs. This could lead to differences in maternal and new-born care and monitoring between study sites.

For peer review only

For peer review only - http://bmjopen.bmj.com/site/about/guidelines.xhtml

1. INTRODUCTION

Malnutrition during pregnancy is a global health problem. Micronutrient deficiencies are associated with adverse developmental outcomes in children, including poorer neurocognitive functioning potentially leading to poorer educational achievement and economic prospects in affected children [1, 2].

Vitamin B-12 deficiency is prevalent across many low and middle-income countries (LMICs)[3-6]. Vegetarian diets and those low in animal source foods are risk factors for B-12 deficiency [7, 8]. During pregnancy sufficient B-12 intake is vital for foetal brain development and deficiency in pregnancy is linked to infant birth defects, intrauterine growth restriction, preterm delivery and neural tube defects [9-14]. Many observational studies document that B-12 deficiency during pregnancy and early infancy adversely affects infant cognitive function [15, 16] including memory [17], language and motor skills [18].

The evidence supporting a causal link between maternal vitamin B-12 levels and infant cognitive outcomes from Randomised Controlled Trials (RCTs) is inconclusive [11]. Maternal B-12 supplementation given as 50mcg a day from ≤14 weeks' gestation through to 6 weeks postpartum did not improve infant neurodevelopmental outcomes at 9 months in a previous trial [11]. However, hyperhomocysteinemia (a marker of B-12 insufficiency) during pregnancy was negatively associated with infant cognitive outcomes, suggesting that the dose or duration of B-12 supplementation may have been inadequate. Additionally, a high rate of participant attrition (51.4%), poor monitoring of supplement compliance, and lack of biochemical evaluation of B-12 status in the supplemented group further limit the conclusiveness of the findings from this trial.

Other RCTs that assessed a 250mcg dose of B-12 per day throughout pregnancy until 3 months' post-partum in Bangladesh [19], and ≥2000mcg from 17 to 34 weeks' gestation in India [20] did not evaluate neurodevelopment outcomes in infancy. Hence, there is insufficient evidence concerning timing, dose, duration and efficacy of B-12 supplementation. To address this evidence gap, we are conducting a double-blind RCTs in one LMIC (India) and one low income country (Nepal), which will evaluate the efficacy of 250mcg/day vitamin B12 from the first trimester to 6 months' post-partum on infant neurodevelopment outcomes measured at age 9 months.

For peer review only - http://bmjopen.bmj.com/site/about/guidelines.xhtml

2. AIMS AND OBJECTIVES

Primary objective

To determine the effect of 250mcg daily oral maternal vitamin B-12 supplementation on infant neurodevelopment as compared to 50mcg.

Secondary objectives

To determine the effect of 250mcg versus 50mcg of oral maternal vitamin B-12 supplementation on biochemical parameters of B-12 status including:

a. Maternal - at the end of the third trimester;
b. Infant - at 9 months post-partum.

Tertiary objectives

To determine the:

a. Socio-economic mediators (income, education, profession) of the relationship between maternal B12 status, supplementation and infant neurodevelopment;
b. Effect of maternal diet on maternal vitamin B12 status, infant B12 status and infant neurodevelopment;
c. Effect of type of milk feeding on infant vitamin B12 status and infant neurodevelopment at 9 months;
d. Effect of infant complementary feeding on infant vitamin B12 status and infant neurodevelopment at 9 months;
e. Effect of maternal iron and vitamin D status in first and third trimester on the relationship between infant vitamin B12 status and infant neurodevelopment at 9 months;
f. Effect of maternal B12 status on infant B12 status and neurodevelopment at 9 months after birth.

For peer review only - http://bmjopen.bmj.com/site/about/guidelines.xhtml

For peer review only

3. METHODOLOGY

Trial Registration:

The study is registered as a primary clinical trial at the WHO International Clinical Trials Registry Platform through the Clinical Trial Registry of India (CTRI/2018/07/015048) and at ClinicalTrials.gov (ID NCT03783104).

Setting

This study will be conducted across two sites, in Delhi, India and Kathmandu, Nepal. Both countries have a documented high prevalence of B-12 deficiency. [5,6]

Participants

Approximately 700 women deliver annually at Sitaram Bhartia Institute of Science and Research (SBISR), New Delhi, India. SBISR is a privately funded, tertiary hospital catering to the higher and middle-income populace. Approximately 22, 000 women deliver annually at Paropakar Maternity and Women's Hospital (PMWH), Kathmandu, Nepal. PMWH is a government hospital facility and caters to people from poorer socio-economic strata. Data from SBISR from our earlier work (2011- 2014) documented that most of the mothers delivering at SBISR were well-educated (96.8% college education), working ladies (63.2%) living in well-earning (Mean Gross Family Income 16000 USD) nuclear families (57.9%) [21]. A directly comparable dataset was not available from PMWH.

Feasibility Data and Preliminary Work

Feasibility of data and preliminary qualitative work was conducted for the trial in 2017-18. Feasibility of recruitment within the given timelines was studied on the basis of previous data available from the study sites. The lead author (JN) examined neurodevelopmental outcome of 413 babies born at SBISR, in a cohort study between 2011 and 2014. The project had a similar antenatal recruitment and post-delivery infant neurodevelopmental follow-up design as proposed in this trial [22]. Approximately 2/3 (67%) of subjects completed the cohort study. The loss to follow-up (33%) and speed of recruitment have been taken into account in the sample size calculations and the recruitment timeline for this trial. No similar recruitment and retention data were available from PMWH.

For peer review only - http://bmjopen.bmj.com/site/about/guidelines.xhtml

Patient and Public Involvement:

To represent the participants' perspective a Patient and Public Involvement (PPI) panel was established consisting of four parent pairs who had delivered a baby within the previous 2 years. One parent pair is also included in continued project development and the Trial Oversight Committee (TOC). We further conducted a qualitative study with pregnant mothers at both study sites to better understand the factors likely to influence the uptake and implementation of the proposed intervention, and proposed barriers to implementation. This qualitative study established that most respondents were unaware of B-12 dietary sources and the importance of B-12. All the participants understood the burden of the intervention and were eager to know about symptoms, treatment and prognosis of B-12 deficiency. Respondents' willingness to accept the intervention and drawing extra blood from the baby as a part of routine sampling for checking B-12 levels guided the study design. Perceived barriers including concerns around potential side effects of the B-12 supplement, relocation and financial difficulties were considered in the design of patient information, consultation and follow-up procedures as follows:

- All participants presenting at the two study sites will be informed of the role and importance of vitamin B-12 in human health and its sources. This will be done through educational banners and posters and through community workshops.
- The initial information about the trial will be given by the doctor providing antenatal care to the mother. Subsequent detailed consent and recruitment will be conducted by study research staff.
- In order to improve compliance and study retention, reminder text messages are sent weekly to participating mothers.
- To incentivise women (sponsoring various biochemical tests at the Indian trial site and covering transportation cost at the Nepal site) at both sites to avoid the risk of financial stress resulting from study participation.
- To add Bradleys' Home Observation for Measurement of the Environment (HOME) inventory to the assessment given the divergent environmental profile of the two study sites [23] in order to assess the infant's environment and possible confounders of neurodevelopment.

The study results will be disseminated globally with academic and health professionals via conferences, presentations and publications. Researchers at each study site will share emerging results with participants during their follow-up visits.

We adapted a pre-validated Food Frequency Questionnaire (FFQ) for Vitamin B-12 intake developed by Gael et al. [24] to the Indian and Nepalese settings. For our purpose, the original Vitamin B12 FFQ

For peer review only - http://bmjopen.bmj.com/site/about/guidelines.xhtml

by Gael et al. containing 30 questions on consumption of food and beverages containing B12 was reviewed independently by two nutritionists. A market survey was done to obtain information on egg and milk products in the diet as well as B-12 fortified items available in local markets of Delhi and Kathmandu. Homemade recipes which include egg and/or dairy products as a main ingredient were incorporated in the FFQ. Locally available household utensils were standardised to measure the amount of food consumed to maintain consistence across the sites.

All research staff from India and Nepal were jointly trained in study design, research methodology, good clinical practice, study protocol and study tools. Training lectures were video recorded and made available to the teams at both study sites as a reference material for continuous training.

Eligibility

Potentially eligible participants are screened at first presentation to the antenatal clinic at ≤12 weeks of gestation in accordance with the following selection criteria.

Inclusion Criteria:

- Vegetarian women defined as a self-reported dietary pattern that includes veganism and/or people who do eat egg and/or do consume milk and/or a portion meat/chicken/fish ≤ once a month;
- Living within an *a-priori* defined geographical area;
  - Delhi – national capital region;
  - Nepal – 10-km radius of PMWH;
- Able to understand at least one of English, Hindi or Nepalese.

Exclusion Criteria:

- Wish for medical termination of pregnancy;
- Aged <18 or >35 years;
- Already on medicinal vitamin B-12 supplementation including B-complex or multivitamins;
- Multiple gestation;
- Diagnosed with a chronic medical condition (including diabetes mellitus, hypertension, heart disease, neurological disease, or thyroid disease);
- Tested positive for hepatitis B, human immunodeficiency virus (HIV), or syphilis;
- Anticipate moving out of the *a-priori* defined geographical area before or after delivery;
- Treated for infertility;

For peer review only - http://bmjopen.bmj.com/site/about/guidelines.xhtml

- Having a known pre-diagnosed mental health disorder including depression, drug or alcohol abuse;
- Currently participating or having participated in another study within four weeks prior to trial start;
- Having a known allergy to vitamin B-12 or another supplement constituent.

Consent

Eligible participants meeting the selection criteria are given time to think over continuation of pregnancy, site of delivery, participation in the trial and any other considerations. They are contacted within 48 hours by the research team about their decision and invited to provide written or video-recorded consent. Participants are able to opt into the trial any time before 12 weeks of gestation.

Randomisation

As presented in Figure 1, all eligible and consenting participants are randomised with an allocation ratio of 1:1 to either the intervention (250mcg), or control (50mcg) group. The randomisation sequence is generated at a single third-party location (non-study site) using a computer-generated sequence stratified for location. The sequence is allocated to serial numbers by a neutral person in each country using sequentially numbered, sealed, opaque envelopes. Codes within envelopes will identify boxes labelled with 5-digit codes. Each box contains enough dosages to cater to the study period of one participant. The strips containing intervention and control doses of B-12 are identical in taste, smell and appearance. All participants, recruiters, developmental therapists, laboratory staff and the data analyst are blinded to the B-12 allocation until final analysis.

Figure 1. **Study design and participant flow through the study**.

Procedure

At enrolment participants are invited to provide demographic details including age, height, weight, ethnicity, education and socio-economic status. Their preferred channel of subsequent communication (phone, text message, email) is also noted. Participants are allocated a study identification number and accompanied for blood sampling. The timing of blood sampling is synchronised with that of any other samples being drawn in the first trimester to minimise

For peer review only - http://bmjopen.bmj.com/site/about/guidelines.xhtml

unnecessary venepunctures. Twelve ml of blood sample is drawn into 2 plain vials (5 ml each) and 2 ml is drawn into an EDTA vial. The EDTA sample is processed immediately for complete blood counts while the plain vial samples is centrifuged at 3500 rpm and the separated serum stored at -70° C. Participants are given B-12 supplement doses to last five days beyond the expected date of their next visit after being fully informed as to the supplement and its administration. Within 48 hours, participants are contacted to confirm initialisation of supplementation and re-emphasise compliance.

Subsequent maternal visits take place monthly until 36 weeks and then weekly. At these visits, all participants visit their obstetrician as part of routine care. Participants also meet with the research staff at a pre-specified location at the study site. To maximise compliance, weekly supplement and pre-appointment reminders are sent via the preferred mode of communication. Acquired morbidity (gestational diabetes, hypertension, and hypothyroidism) and medication history are noted in case record forms (CRFs). Empty supplement packets are collected at each appointment as an indicator of adherence. In the third trimester, mothers' dietary B-12 intake is assessed using a pre-standardised and adapted FFQ and blood sampling will be repeated using the same procedures as the first trimester.

Neonatal assessment: During childbirth, a medical officer and/or paediatrician monitors the birth and post-delivery course of neonates for any influencing morbidity. Gestational age, growth retardation, congenital abnormalities and APGAR (appearance, pulse, grimace, activity, respiration) score is also routinely documented for neonates. Cord pH is measured for all new-borns with delayed cry and who require any intervention beyond the basic steps of resuscitation (ambu bagging, intubation, or pharmacological treatment). Any seizures, neurological problems, hypoglycaemia, hypothermia, hyperbilirubinemia are screened, diagnosed, evaluated, and managed as per institutional guidelines. As per the standard practice, all new-borns are screened for hearing deficits, vision, and critical congenital heart disease as per institutional protocols. Preterm neonates transferred to the neonatal unit are followed as per their customized follow-up and early intervention plans as recommended by the primary care provider. Neurodevelopment and biochemical assessment of preterm neonates is carried out as per their corrected ages.

Infant follow-up is conducted at 7-14 days, 4, 8, 12 and 16 weeks and at 6 and 9 months of age. At these visits, the infants undergo routine care as well as study specific evaluations.

Routine care: Each mother-infant dyad sees a paediatrician for age-appropriate routine care, which involves clinical evaluation, preventative care and vaccination as per standard procedure.

For peer review only - http://bmjopen.bmj.com/site/about/guidelines.xhtml

Anthropometric measurements and signs of micronutrient deficiency (anaemia and rickets) are recorded during each visit as part of routine care. Metabolic disorders (congenital hypothyroidism, congenital adrenal hyperplasia, cystic fibrosis, biotinidase deficiency, phenylketonuria and galactosemia) are screened for and maternal support for breastfeeding by a paediatrician or lactation counsellor is provided at 7-14 days.

Study specific: Supplements are provided to mothers at each visit, with instructions for usage until 6 months after childbirth. Any morbidities and deficiencies are recorded and treated as per institutional guidelines. Maternal and infant tolerance for the supplementation including any gastrointestinal symptoms are recorded at each visit, as is maternal compliance. Provision of supplements to the mother in both groups is be stopped at 6 months post-partum.

The hospital evaluation at 9 months (±14 days) includes neurodevelopmental assessment by a developmental therapist using the Developmental Assessment Scales for Indian Infants (DASII) - an adapted and validated version of the Bayley's Scale for Infant Development Third Edition (BSID III) and blood sampling for estimation of Hb, vitamin B-12, homocysteine and holo-transcobalamin concentrations [25]. The DASII motor scale assesses control of gross and fine motor muscle group whilst cognitive, personal and social skills development is assessed through the mental scale. All neurodevelopmental assessments is conducted by a trained developmental therapist at the institute in a soundproof room. In premature babies corrected age is used to calculate developmental quotients. Additional assessments include a complementary feeding assessment by a nutritionist using a 72-hour dietary recall and home environment assessment using the adapted Bradley HOME inventory [24] by a trained field worker.

Compliance: Research staff dispense blister packaging of ten B-12 capsules and study specific bags for participants to store, carry and keep supplements in one place. In case of loss of supplement or inability to come for follow-up, participants are able to contact the researcher at any time to request more supplements. The supplement packaging is numbered with a serial number. To maximise compliance, weekly reminders are sent through the preferred mode of communication as specified by the trial participants. Each participant is provided with a bag to save empty packaging strips and instructed to carry the bag with her at the next visit to monitor compliance.

Biochemical analysis

All samples are processed as one batch at study completion. Maternal samples are assessed for blood levels for B-12, homocysteine, holo-transcobalamin, 25(OH)D, folate, CBC and RBC indices in the first

For peer review only - http://bmjopen.bmj.com/site/about/guidelines.xhtml

and third trimesters. Infant blood is assessed for Hb, Vitamin B-12, Homocysteine and Holo-transcobalamin measurements.

Outcomes:

The following study outcomes will be investigated:

Primary Outcome:

- DASII scores for infants at 9 months (±2 weeks) post-partum

Secondary Outcome:

- Change in B-12 status (vitamin B12 level, homocysteine, and Holotranscobalamin) of mother between first (weeks' gestation) and third trimester ($\geq$ 27 weeks' gestation);
- B-12 status (vitamin B12 level, homocysteine, and Holotranscobalamin) of infants at 9 months (±2 weeks) post-partum.

Exploratory Outcome(s):

- Haemoglobin (Hb) of infants at 9 months ($\pm$14 days) post-partum;
- Infant anthropometry including weight, height, length and head circumference at 9 months after birth in all subjects.

Sample size considerations

A planned sample of 720 pregnant women, 360 per study site, will be randomly assigned to experimental (*n*=360; *n*=180 in per study site) or control (*n*=360; *n*=180 in per study site) groups. This is based on the number of infants needed to complete the neurodevelopmental screen in each of the two randomised groups to detect a difference in Development Quotient (DQ) at nine months (primary outcome) of 2 points (22). The difference of 2 DQ points was chosen because it is the smallest effect size thought to be clinically significant and hence a null trial result is likely to exclude a clinically relevant effect of maternal B12 supplementation on infant cognitive function. The sample size has been inflated by 35% to account for attrition and assumes 90% statistical power and a 5% two-sided significance level.

Statistical analysis

The primary and secondary outcomes is analysed using an intention-to-treat analysis by a blinded statistician. Comparability of participants assigned to study groups at enrolment is assessed using tests of association for categorical variables and by fitting generalised linear models for continuous

For peer review only - http://bmjopen.bmj.com/site/about/guidelines.xhtml

variables. These models include interaction terms to explore conditional differences between the trial's arms. If necessary, transformations are applied to the response variables to fulfil the model's assumptions. Covariates include trial site, infant feeding, gender, feeding by gender, weight *z*-score at birth, weight gain between birth and 3 months, and maternal body mass index (BMI). *Z*-scores for height and weight are calculated according to the World Health Organisation [26] growth standards using the R language for statistical computing [27].

We will examine the impact of B-12 supplementation on randomised groups in two subgroups – those that have any of the following conditions vs. all others (high risk vs. low risk):

- Maternal morbidity during pregnancy will be classified as high-risk (including gestational diabetes, pregnancy-induced hypertension/pre-eclampsia/eclampsia and absent or reversed end diastolic flow); and low-risk;
- Birth weight *z*-score categorized as high-risk (small for gestational age [SGA]: <10th centile for birth weight); and low-risk (appropriate for weight [>10th centile for birthweight]);
- Gestation categorized as preterm (low-risk; 33-37 weeks gestation); very preterm (high-risk; 28-32 weeks gestation); and term (low-risk: >37 weeks gestation). Extremely preterm (<28 weeks) will be exited;
- Higher risk neonatal course categorized as high risk (if neonate has: APGAR score <7 at 1 minute, hypoglycaemia, hyperbilirubinemia or prolonged neonatal intensive care stay >7 days); and low risk.
- Infants with documented neurological disease/anomaly/illness will be sub-grouped separately (high risk) and compared to those with no disease/anomaly illness (low risk).

Data management and dissemination

Data will be collected under the regulation of the data protection and management guidelines given by Governments of India and Nepal, and as per the UK Data Protection Act 2018. Data will be securely stored in an E-database with password protection. The E-database was designed using Access. Live data entry will be conducted at the study sites and tested for accuracy using duplicate data entry at an offsite location at periodic intervals.

The study results will be disseminated globally with academic and health professionals via conferences, presentations and publications. Researchers at each study site will share emerging results with participants during their follow-up visits. Regular site meetings with press offices, short films, radio spots, periodic blog posts, Twitter and Facebook messages, and online articles are also

For peer review only - http://bmjopen.bmj.com/site/about/guidelines.xhtml

planned to strengthen the trial's online presence and reach young and middle-aged internet users for whom the information may be of interest.

Qualitative evaluation proposed:

The qualitative sub-study aims to explore knowledge, attitudes and perceptions of pregnant women towards B-12 supplementation and child development, and potential barriers to implementation. Better understanding these domains across the two study sites may inform the perceived need for the intervention, help define patient-centric outcomes and allow the evaluation of any differences between the two arms.

We will use convenience sampling and estimate interviewing 30 participants across both trial sites. We will develop and pilot a semi-structured topic guide in collaboration with PPI representatives serving on the Trial Steering Committee (TSC). The topic guide will be designed in English and translated into either Hindi or Nepali for administration across the study sites. The topic guide will then be back translated to English to ensure consistency and accuracy of content. It is intended to help direct the interview whilst remaining sensitive to unsolicited themes. An interviewer with prior training and experience in qualitative methods will facilitate the semi-structured interviews.

Data will be collected in quiet rooms in either hospital, away from other patients and staff. Interviews will be facilitated in English, Hindi or Nepalese. All interviews will be audio recorded, transcribed, translated into English (where necessary) and then crosschecked against the original recording. The translated transcripts will then be coded using the Atlas Ti 7.2 software for qualitative analysis. [28] A research team member will code and categorise data, and prepare an initial narrative. A final report will then be prepared with input from the interviewers and wider research team.

Ethical considerations:

The Study Protocol (10th December 2018. Version: 8.0), Informed Consent Form, Participant Information Sheet, Tool and other supportive materials have been approved by the Institutional Ethics Committee (IEC) at SBISR (FL/SBISR/IEC/2018-01); PHFI (TRC-IEC-380/18); PMWH (59-11-433); Nepal Health Research Council Ethical Review Board (NHRC) (Reg No. 761/2018) and the University College London (UCL) Research Ethics Committee (10901/001). The Investigators at each study site has submitted and, where necessary, obtained approval from the above parties for all substantial amendments to the original approved documents.

The trial is being conducted in accordance to the principles and rules of the Declaration of Helsinki. Informed and written consent is obtained from all participants by the research teams at each study site. The trial is complying with the Data Protection Act (UK), 2018 ensuring the participants'

For peer review only

anonymity. The participants are identified only by a participant identification number on all trial documents and electronic databases. Every effort is made to arrange interviews and examination at a time convenient to the participants without causing much disruption in their daily routine. Study data is stored securely and is only accessible to trial staff and authorised personnel. The Principal Investigator at each study site will submit an annual progress report, end of trial notification and a final report to the Institutional Ethics Committee, host organisations and the funders.

We used the SPIRIT checklist for writing our protocol [29].

DISCUSSION

This study will have considerable impact on current and future policy initiatives focusing on micronutrient supplementation in India and Nepal by underpinning micronutrient supplementation policy in India as envisaged in the recently released "National Health Policy", and the micronutrient initiative in Nepal. The project will provide key insights to strengthen existing national nutrition programmes like "National Iron plus Initiative", "Janini Suraksha Yojna" and "Janini Shishu Suraksha Kariyakram" initiated by the Government of India and similar initiatives by the Government of Nepal. Because of the high worldwide prevalence of maternal B-12 deficiency (74%), the study findings will be highly relevant for maternal and neonatal health in nutritionally vulnerable and at-risk populations globally. The work will also provide evidence on the utility, dose, duration and time of initiation of Vitamin B12 supplementation in pregnant women/infants.

As one of the few large-scale trials of B-12 supplementation in pregnancy, a key aim of the project is to reset significant elements of the research agenda in maternal and child health. We aim to contribute to the evidence base on improving neurodevelopmental outcomes in vulnerable infants. While a positive result for the trial would help develop an intervention program to reduce developmental delay using Vitamin B-12 as a supplementation in pregnant women, a negative result would also add to existing knowledge and lay the groundwork for future work on the subject.

For peer review only

DECLARATIONS

Acknowledgements: CL and ML were partly supported by the National Institute for Health Research (NIHR) Collaborations for Leadership in Applied Health Research and Care (CLARCH) North Thames at the time of this work. We would also like to thank our Patient and Public Involvement (PPI) panel.

Author Statements: JN, MM, and ML conceived the study and AS, MH, MCB, JG, RP contributed to the study design. SR and DN conducted the qualitative study, adaptation of FFQ, standardisation of

For peer review only - http://bmjopen.bmj.com/site/about/guidelines.xhtml

questionnaires and organised the training workshop. SR, DN, KA and CL drafted the manuscript. JN, MM and ML finalised the manuscript, and all authors assisted in developing the protocol and have read and approved the final manuscript.

Funding: The study is funded by the Newton Fund: The Department of Biotechnology (DBT) India, in collaboration with Department of International Development (DFID), the Economic and Social Research Council (ESRC) and the Medical Research Council (MRC) consortium (Grant Number MR/R020396/1). This research was supported by the National Institute for Health Research (NIHR) Biomedical Research Centre based at UCL Great Ormond Street Institute of Child Health/Great Ormond Street Hospital NHS Foundation Trust.

Competing interests: All authors declare no competing interests.

Provenance and peer review: Not commissioned; externally peer reviewed

Data sharing statement: For access to the data set, a formal request should be sent to the MATCOBIND study group. The request will only be considered when the results of the study have been published.

For peer review only

REFERENCES

1. Liu J. Raine A. The effect of childhood malnutrition on externalizing behavior. Curr Opin Pediatr 2006; 18:565–70.
2. Victora CG, Adair L, Fall C, et al. Maternal and child undernutrition: consequences for adult health and human capital. Lancet. 2008;371(9609):340–57.
3. Allen, LH. Folate and vitamin B12 status in the Americas. Nutr Rev. 2004 Jun; 62(6): S29-33.
4. Allen, LH. Causes of vitamin B12 and folate deficiency. Food Nutr Bull. 2008 Jun;29(2):20-34.
5. Ulak M, Chandyo RK, Adhikari RK, et al. Cobalamin and folate status in 6 to 35 months old children presenting with acute diarrhea in Bhaktapur, Nepal. PLoS One 2014;9: e90079.
6. Taneja S, Bhandari N, Strand TA, Sommerfelt H, Refsum H, Ueland PM, Scheede J, Bahl R, Bhan MK. Cobalamin and folate status in infants and young children in a low-to-middle income community in India. Am J Clin Nutr. 2007; 86:1302–1309.
7. Sklar R. Nutritional vitamin B12 deficiency in a breast-fed infant of a vegan-diet mother. Clin Pediatr (Phila). 1986;25(4):219–21.
8. Kuhne T, Bubl R, Baumgartner R. Maternal vegan diet causing a serious infantile neurological disorder due to vitamin B12 deficiency. Eur J Pediatr. 1991;150(3):205-208.
9. Lee C, Maza Stern B, Lakhanpaul M, Parikh P. Associations between the household environment and stunted child growth in rural India: a cross-sectional analysis. UCL Open: Environment. 2019: doi: 10.14324/111.444/000015.v1.
10. Ganguly P, Alam SF. Role of homocysteine in the development of cardiovascular disease. *Nutr J*. 2015; 14:6. 2015 Jan 10. doi:10.1186/1475-2891-14-6.
11. Srinivasan K, Thomas T, Kapanee ARM, Ramthal A, Bellinger DC, Bosch RJ et al. Effects of maternal vitamin B12 supplementation on early infant neurocognitive outcomes: a randomised controlled clinical trial. Matern Child Nutr. 2016: doi:10.1111/mcn/12345.
12. Black MM. Effects of Vitamin B12 and Folate Deficiency on Brain Development in Children. Food Nutr Bull. 2008;29(2): S126–31.
13. Molloy AM, Kirke PN, Troendle JF, Burke H, Sutton M, Brody LC, Scott JM, Mills JL. Maternal vitamin B12 status and risk of neural tube defects in a population with high neural tube defect prevalence and no folic acid fortification. Pediatrics. 2009; 123:917–923.
14. Refsum H. Folate, vitamin B12 and homocysteine in relation to birth defects and pregnancy outcome. Br J Nutr. 2001;85: S109–S113.

For peer review only

15. Siddiqua JT. Vitamin B12 deficiency in pregnancy and lactation: is there a need for pre and post-natal supplementation? J Nutr Disord Ther. 2014;4(2):1-8.
16. Dror DK, Allen LH. Effect of vitamin B12 deficiency on neurodevelopment in infants: current knowledge and possible mechanism. Nutr Rev. 2008; 66:250-255.
17. Bhate V, Deshpande S, Bhat D, Joshi N, Ladkat R, Watve S, et al. Vitamin B12 status of pregnant Indian women and cognitive function in their 9-year-old children. Food Nutr Bull. 2008; 29:249– 254.
18. Torksvik IK, Ueland PM, Markestad T et al. Cobalamin supplementation improves motor development and regurgitations in infants: results from a randomised intervention study. BMC Pediatr. 2015; 15:2018.
19. Siddiqua TJ, Ahmad SM, Ahsan SB, Rashid M, Roy A, Rahman SM et al. Vitamin B12 supplementation during pregnancy and postpartum improves B12 status of both mothers and infants but vaccine response in mothers only: a randomised control in Bangladesh. Eur J Nutr. 2016; 55(1)281-293.
20. Katre P, Bhat D, Lubree H, Otiv S, Joshi S, Joglekar C et al. Vitamin B12 and folic acid supplementation and plasma total homocysteine concentrations in pregnant women India women with low B12 and high folate status. Asia Pac J Clin Nutr. 2010;19(3):335-43.
21. Nagpal, J., Sachdeva, A., Sengupta Dhar, R., Bhargava, V., & Bhartia, A. Widespread non-adherence to evidence-based maternity care guidelines: a population-based cluster randomised household survey. BJOG: An International Journal of Obstetrics & Gynaecology. 2014; 122(2), 238–247.doi:10.1111/1471-0528.13054.
22. Nagpal J, Bhargava VL, Sengupta R, Dhall S, Agarwal N, Gulati K. A prospective cohort study to evaluate the utility of antenatal doppler velocimetry in prediction of fetal malnutrition, neonatal morbidity, mortality and neurodevelopmental outcome: Final report submitted to ICMR 2015. Downloaded from url: http://14.139.60.56/ICMRFINALREPORT/SQL_FR_ABS_PDF_Disp.aspx?Type=FR&Proj_No=2009-01550&SLNO=1
23. BradleyR.H., Caldwell B.M., Rock S.L.,Ramey C.T.,Barnard K.E.,Gray C. et al. Home environment and cognitive development in the first 3 years of life: a collaborative study involving six sites and three ethnic groups in NorthAmerica. Developmental Psychology. 1989; 25 (2),217.
24. Mearns GJ, Rush EC. Screening for inadequate dietary vitamin B12 intake in South Asian women using a nutrient-specific, semi-quantitative food frequency questionnaire. Asia Pacific journal of clinical nutrition. 2016 Nov 18.

For peer review only - http://bmjopen.bmj.com/site/about/guidelines.xhtml

25. Patni B. Developmental Assessment Scales for Indian Infants (DASII). Indian J Pract Pediatr. 2012;14(4):409–12.
26. WHO Multicentre Growth Reference Study Group. WHO Child Growth Standards based on length/height, weight and age. Acta paediatrica (Oslo, Norway: 1992). Supplement. 2006 Apr; 450:76.
27. R Core Team (2013). R: A language and environment for statistical computing. R Foundation for Statistical Computing, Vienna, Austria. Available via: //www.R-project.org.
28. ATLAS.ti: The Qualitative Data Analysis; Research Software.2019.
29. Chan A-W, Tetzlaff JM, Altman DG, Laupacis A, Gøtzsche PC, Krleža-Jerić K, Hróbjartsson A, Mann H, Dickersin K, Berlin J, Doré C, Parulekar W, Summerskill W, Groves T, Schulz K, Sox H, Rockhold FW, Rennie D, Moher D. SPIRIT 2013 Statement: Defining standard protocol items for clinical trials. Ann Intern Med. 2013;158(3):200-207."

For peer review only

For peer review only - http://bmjopen.bmj.com/site/about/guidelines.xhtml

For peer review only

For peer review only - http://bmjopen.bmj.com/site/about/guidelines.xhtml

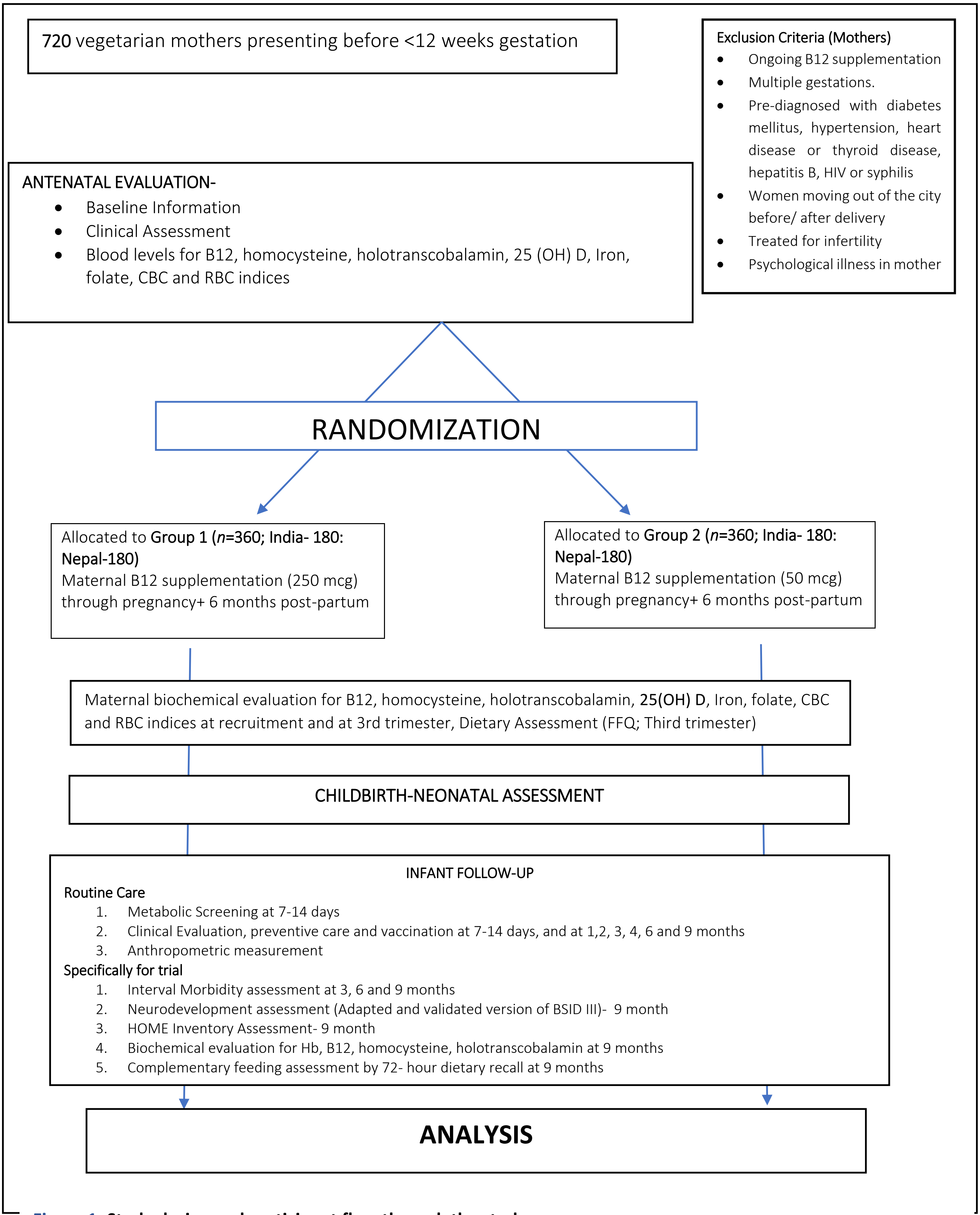

Figure 1. Study design and participant flow through the study.

For peer review only

Supplementary Material: Table 1. Visit schedule

Study Plan	Initial Contact (<12 weeks gestation)		M1	M2-M9	M10	At Birth	I1	I2-I6	I7
**Schedule**	**Screening**	**Recruitment**	**Antenatal Follow-up**	**Antenatal Follow-up (monthly until second trimester, fortnightly in the third trimester**	**Antenatal Follow-up (weekly after 36 weeks until delivery)**		**Infant 7-14 day**	**Infant follow-up: At months-1 (± 1 wk), 2 (± 2 wks), 3 (± 2 wks), 4 (± 2 wks) & 6 (± 2 wks)**	**Infant follow-up Age 9 months ± 14 days;**
First Screening Visit	X								
Maternal Inclusion/Exclusion criteria	X	X							
Informed consent of the participant		X							
Questionnaire Administration		X							
Randomization		X							
Supplementation		X	X	X	X	X	X	X	
Blood Sampling (1st trimester)		X							
Home Visit (if required)			X	X	X	X	X	X	X
Monitoring of the Compliance			X	X	X	X	X	X	X
Adverse events			X	X	X	X	X	X	X
Blood Sampling (3rd trimester, >26 weeks gestation)				X					
Mother Food Frequency Questionnaire (>26 weeks gestation)				X					
Infant Anthropometry (Weight/length/Head Circumference)						X	X	X	X
Monitoring for neonatal morbidity*						X	X	X	X
Application of Exit Criteria (specified earlier)			X	X	X	X	X	X	X
Metabolic Screening of infant							X		
Infant morbidity							X	X	X
Infant blood sampling									X
Complementary feeding 72 hr. Dietary Recall									X
DASII Scale									X

For peer review only

HOME Inventory									X

Table 2. Blood samples and measurements

Measurement	Estimate	Reference
Vitamin B12	Enhanced CLIA	Johnson & Johnson, USA
Iron	Pyridlazodye	Johnson & Johnson, USA
Folate	Enhanced CLIA	Beckman Coulter, USA
Complete blood count (CBC)		Beckman Coulter, USA
Red blood count (RBC)	Impedance	Beckman Coulter, USA
Homocysteine and holotranscobalamin	CMIA	

For peer review only

Reporting checklist for protocol of a clinical trial.

Based on the SPIRIT guidelines.

Instructions to authors

Complete this checklist by entering the page numbers from your manuscript where readers will find each of the items listed below.

Your article may not currently address all the items on the checklist. Please modify your text to include the missing information. If you are certain that an item does not apply, please write "n/a" and provide a short explanation.

Upload your completed checklist as an extra file when you submit to a journal.

In your methods section, say that you used the SPIRITreporting guidelines, and cite them as:

Chan A-W, Tetzlaff JM, Altman DG, Laupacis A, Gøtzsche PC, Krleža-Jerić K, Hróbjartsson A, Mann H, Dickersin K, Berlin J, Doré C, Parulekar W, Summerskill W, Groves T, Schulz K, Sox H, Rockhold FW, Rennie D, Moher D. SPIRIT 2013 Statement: Defining standard protocol items for clinical trials. Ann Intern Med. 2013;158(3):200-207

		Reporting Item	Page Number
**Administrative information**			
Title	#1	Descriptive title identifying the study design, population, interventions, and, if applicable, trial acronym	1

For peer review only

Trial registration	#2a	Trial identifier and registry name. If not yet registered, name of intended registry	5
Trial registration: data set	#2b	All items from the World Health Organization Trial Registration Data Set	5
Protocol version	#3	Date and version identifier	14
Funding	#4	Sources and types of financial, material, and other support	15
Roles and responsibilities: contributorship	#5a	Names, affiliations, and roles of protocol contributors	1
Roles and responsibilities: sponsor contact information	#5b	Name and contact information for the trial sponsor	15
Roles and responsibilities: sponsor and funder	#5c	Role of study sponsor and funders, if any, in study design; collection, management, analysis, and interpretation of data; writing of the report; and the decision to submit the report for publication, including whether they will have ultimate authority over any of these activities	15
Roles and responsibilities: committees	#5d	Composition, roles, and responsibilities of the coordinating centre, steering committee, endpoint adjudication committee, data management team, and other individuals or groups overseeing the trial, if applicable (see Item 21a for data monitoring committee)	

For peer review only

**Introduction**			
Background and rationale	#6a	Description of research question and justification for undertaking the trial, including summary of relevant studies (published and unpublished) examining benefits and harms for each intervention	4
Background and rationale: choice of comparators	#6b	Explanation for choice of comparators	4
Objectives	#7	Specific objectives or hypotheses	4
Trial design	#8	Description of trial design including type of trial (eg, parallel group, crossover, factorial, single group), allocation ratio, and framework (eg, superiority, equivalence, non-inferiority, exploratory)	
**Methods: Participants, interventions, and outcomes**			
Study setting	#9	Description of study settings (eg, community clinic, academic hospital) and list of countries where data will be collected. Reference to where list of study sites can be obtained	5
Eligibility criteria	#10	Inclusion and exclusion criteria for participants. If applicable, eligibility criteria for study centres and	7

For peer review only

		individuals who will perform the interventions (eg, surgeons, psychotherapists)	
Interventions: description	#11a	Interventions for each group with sufficient detail to allow replication, including how and when they will be administered	9
Interventions: modifications	#11b	Criteria for discontinuing or modifying allocated interventions for a given trial participant (eg, drug dose change in response to harms, participant request, or improving / worsening disease)	
Interventions: adherance	#11c	Strategies to improve adherence to intervention protocols, and any procedures for monitoring adherence (eg, drug tablet return; laboratory tests)	11
Interventions: concomitant care	#11d	Relevant concomitant care and interventions that are permitted or prohibited during the trial	10-11
Outcomes	#12	Primary, secondary, and other outcomes, including the specific measurement variable (eg, systolic blood pressure), analysis metric (eg, change from baseline, final value, time to event), method of aggregation (eg, median, proportion), and time point for each outcome. Explanation of the clinical relevance of chosen efficacy and harm outcomes is strongly recommended	11
Participant timeline	#13	Time schedule of enrolment, interventions (including any run-ins and washouts), assessments, and visits for participants. A schematic diagram is highly recommended	

For peer review only

		(see Figure)	
Sample size	#14	Estimated number of participants needed to achieve study objectives and how it was determined, including clinical and statistical assumptions supporting any sample size calculations	11
Recruitment	#15	Strategies for achieving adequate participant enrolment to reach target sample size	
**Methods: Assignment of interventions (for controlled trials)**			
Allocation: sequence generation	#16a	Method of generating the allocation sequence (eg, computer-generated random numbers), and list of any factors for stratification. To reduce predictability of a random sequence, details of any planned restriction (eg, blocking) should be provided in a separate document that is unavailable to those who enrol participants or assign interventions	8
Allocation concealment mechanism	#16b	Mechanism of implementing the allocation sequence (eg, central telephone; sequentially numbered, opaque, sealed envelopes), describing any steps to conceal the sequence until interventions are assigned	8-9
Allocation: implementation	#16c	Who will generate the allocation sequence, who will enrol participants, and who will assign participants to interventions	8

For peer review only

Blinding (masking)	#17a	Who will be blinded after assignment to interventions (eg, trial participants, care providers, outcome assessors, data analysts), and how	8
Blinding (masking): emergency unblinding	#17b	If blinded, circumstances under which unblinding is permissible, and procedure for revealing a participant's allocated intervention during the trial	
**Methods: Data collection, management, and analysis**			
Data collection plan	#18a	Plans for assessment and collection of outcome, baseline, and other trial data, including any related processes to promote data quality (eg, duplicate measurements, training of assessors) and a description of study instruments (eg, questionnaires, laboratory tests) along with their reliability and validity, if known. Reference to where data collection forms can be found, if not in the protocol	12
Data collection plan: retention	#18b	Plans to promote participant retention and complete follow-up, including list of any outcome data to be collected for participants who discontinue or deviate from intervention protocols	13
Data management	#19	Plans for data entry, coding, security, and storage, including any related processes to promote data quality (eg, double data entry; range checks for data values).	13

For peer review only

		Reference to where details of data management procedures can be found, if not in the protocol	
Statistics: outcomes	#20a	Statistical methods for analysing primary and secondary outcomes. Reference to where other details of the statistical analysis plan can be found, if not in the protocol	12-13
Statistics: additional analyses	#20b	Methods for any additional analyses (eg, subgroup and adjusted analyses)	
Statistics: analysis population and missing data	#20c	Definition of analysis population relating to protocol non-adherence (eg, as randomised analysis), and any statistical methods to handle missing data (eg, multiple imputation)	
**Methods: Monitoring**			
Data monitoring: formal committee	#21a	Composition of data monitoring committee (DMC); summary of its role and reporting structure; statement of whether it is independent from the sponsor and competing interests; and reference to where further details about its charter can be found, if not in the protocol. Alternatively, an explanation of why a DMC is not needed	
Data monitoring: interim analysis	#21b	Description of any interim analyses and stopping guidelines, including who will have access to these interim results and make the final decision to terminate the trial	
Harms	#22	Plans for collecting, assessing, reporting, and managing solicited and spontaneously reported adverse events and other unintended effects of trial interventions or trial	

For peer review only

		conduct	
Auditing	#23	Frequency and procedures for auditing trial conduct, if any, and whether the process will be independent from investigators and the sponsor	
**Ethics and dissemination**			
Research ethics approval	#24	Plans for seeking research ethics committee / institutional review board (REC / IRB) approval	14-15
Protocol amendments	#25	Plans for communicating important protocol modifications (eg, changes to eligibility criteria, outcomes, analyses) to relevant parties (eg, investigators, REC / IRBs, trial participants, trial registries, journals, regulators)	14-15
Consent or assent	#26a	Who will obtain informed consent or assent from potential trial participants or authorised surrogates, and how (see Item 32)	14-15
Consent or assent: ancillary studies	#26b	Additional consent provisions for collection and use of participant data and biological specimens in ancillary studies, if applicable	14-15
Confidentiality	#27	How personal information about potential and enrolled participants will be collected, shared, and maintained in order to protect confidentiality before, during, and after the trial	14-15
Declaration of	#28	Financial and other competing interests for principal	16

For peer review only

interests		investigators for the overall trial and each study site	
Data access	#29	Statement of who will have access to the final trial dataset, and disclosure of contractual agreements that limit such access for investigators	13-14
Ancillary and post trial care	#30	Provisions, if any, for ancillary and post-trial care, and for compensation to those who suffer harm from trial participation	
Dissemination policy: trial results	#31a	Plans for investigators and sponsor to communicate trial results to participants, healthcare professionals, the public, and other relevant groups (eg, via publication, reporting in results databases, or other data sharing arrangements), including any publication restrictions	13
Dissemination policy: authorship	#31b	Authorship eligibility guidelines and any intended use of professional writers	X
Dissemination policy: reproducible research	#31c	Plans, if any, for granting public access to the full protocol, participant-level dataset, and statistical code	
**Appendices**			
Informed consent materials	#32	Model consent form and other related documentation given to participants and authorised surrogates	X
Biological specimens	#33	Plans for collection, laboratory evaluation, and storage of biological specimens for genetic or molecular analysis in the current trial and for future use in ancillary studies, if applicable	X

For peer review only

None The SPIRIT checklist is distributed under the terms of the Creative Commons Attribution License CC-BY-ND 3.0. This checklist can be completed online using https://www.goodreports.org/, a tool made by the EQUATOR Network in collaboration with Penelope.ai

For peer review only

Efficacy of maternal B-12 supplementation in vegetarian women for improving infant neurodevelopment: Protocol for the MATCOBIND multicentre, double-blind, randomised controlled trial

Journal:	*BMJ Open*
Manuscript ID	bmjopen-2019-034987.R1
Article Type:	Protocol
Date Submitted by the Author:	06-Dec-2019
Complete List of Authors:	Nagpal, Jitender; Sitaram Bhartia Institute of Science and Research, Pediatrics<br>Mathur, Manu; Public Health Foundation of India,<br>Rawat , Swapnil ; Sitaram Bhartia Institute of Science and Research, Pediatrics<br>Nagrath, Deepti; Public Health Foundation of India,<br>Lee, Charlotte; University College London, Institute of Child Health<br>Singhal, Atul ; University College London Institute of Child Health<br>Heys, Michelle; University College London Institute of Child Health, University College London Institute of Child Health<br>Cortina Borja, Mario; UCL, Institute of Child Health<br>Augustin, Katrin ; University College London, Institute of Child Health<br>Gautam , Jageshwor; Paropakar Maternity & Women's Hospital<br>Pant , Rajendra; Paropakar Maternity & Women's Hospital<br>Lakhanpaul, Monica; University College London, Institute of Child Health
**Primary Subject Heading**:	Nutrition and metabolism
Secondary Subject Heading:	Paediatrics, Obstetrics and gynaecology
Keywords:	Maternal medicine < OBSTETRICS, PAEDIATRICS, NUTRITION & DIETETICS, Developmental neurology & neurodisability < PAEDIATRICS, Community child health < PAEDIATRICS

I, the Submitting Author has the right to grant and does grant on behalf of all authors of the Work (as defined in the below author licence), an exclusive licence and/or a non-exclusive licence for contributions from authors who are: i) UK Crown employees; ii) where BMJ has agreed a CC-BY licence shall apply, and/or iii) in accordance with the terms applicable for US Federal Government officers or employees acting as part of their official duties; on a worldwide, perpetual, irrevocable, royalty-free basis to BMJ Publishing Group Ltd ("BMJ") its licensees and where the relevant Journal is co-owned by BMJ to the co-owners of the Journal, to publish the Work in this journal and any other BMJ products and to exploit all rights, as set out in our licence.

The Submitting Author accepts and understands that any supply made under these terms is made by BMJ to the Submitting Author unless you are acting as an employee on behalf of your employer or a postgraduate student of an affiliated institution which is paying any applicable article publishing charge ("APC") for Open Access articles. Where the Submitting Author wishes to make the Work available on an Open Access basis (and intends to pay the relevant APC), the terms of reuse of such Open Access shall be governed by a Creative Commons licence – details of these licences and which Creative Commons licence will apply to this Work are set out in our licence referred to above.

Other than as permitted in any relevant BMJ Author's Self Archiving Policies, I confirm this Work has not been accepted for publication elsewhere, is not being considered for publication elsewhere and does not duplicate material already published. I confirm all authors consent to publication of this Work and authorise the granting of this licence.

For peer review only

TITLE PAGE

Title

Efficacy of maternal B-12 supplementation in vegetarian women for improving infant neurodevelopment: Protocol for the MATCOBIND multicentre, double-blind, randomised controlled trial

Running Head: Maternal Vit-B12 and Infant Neurodevelopment Protocol

For peer review only

Authors

Jitender Nagpal[a], Manu Raj Mathur[b], Swapnil Rawat[a], Deepti Nagrath[b], Charlotte Lee[c], Atul Singhal[c], Michelle Heys[c], Mario Cortina-Borja[c], Katrin Augustin[c],Jageshwor Gautam[d], Rajendra Pant[d], Monica Lakhanpaul[c*]

a. *Sitaram Bhartia Institute of Science and Research (SBISR), B16, Qutab Institutional Area, New Delhi, 110016, India. jitendernagpal@gmail.com; swapnil.rawat@gmail.com*
b. *Public Health Foundation of India (PHFI)* Plot Number 47, Sector 44, Institutional Area, Gurugram – 122002, India. manu.mathur@phfi.org ; deepti.nagrath@phfi.org
c. *UCL Great Ormond Street Institute of Child Health, University College London, 30 Guildford Street, London, WC1N 1EH, United Kingdom. a.singhal@ucl.ac.uk; m.heys@ucl.ac.uk; m.cortina@ucl.ac.uk; k.augustin@ucl.ac.uk; c.lee.12@ucl.ac.uk; m.lakhanpaul@ucl.ac.uk .*
d. *Paropakar Maternity and Womens' Hospital (PMWH), Thapathali, Kathmandu, Nepal. drjgautam48@gmail.com; rajendrapant8@gmail.com .*

*Corresponding Author

Professor Monica Lakhanpaul; UCL Great Ormond Street Institute of Child Health, University College London, 30 Guildford Street, London, WC1N 1EH, United Kingdom; m.lakhanpaul@ucl.ac.uk .

Word Count

Abstract: 247

Main text: 4475

Tables: 1

Figures: 1

Key Words- Maternal Health, Vitamin B-12, Infant Neurodevelopment

ABSTRACT

Introduction: Vitamin B-12 deficiency is widely prevalent across many low- and middle-income countries especially where the diet is low in animal sources of food. While many observational studies document the relationship between B-12 deficiency in pregnancy and infant cognitive functions including memory, language and motor skills, evidence from clinical trials documenting preventability/reversibility on supplementation is sparse and inconclusive.

Methods and Analysis: 720 vegetarian pregnant women in the first trimester will be recruited in this multicentric double blind randomised control trial from two sites (360 each). Enrolled participants will be randomised into two groups to receive either 250 mcg methylcobalamin or 50 mcg (quasi control dose) as oral supplements from enrolment to 6 months post-partum while ensuring blinding and allocation concealment. All mothers and their infants will continue to receive standard care at their respective sites. The infants' neurodevelopment at 9 months of age will be compared between the two groups as the primary outcome. Biochemical markers of B-12 status will be assessed for the mother at the first trimester and third trimester while the infants' B-12 status will be assessed at 9 months of age as secondary outcomes of efficacy.

Ethical Considerations and Dissemination: The Study Protocol was approved by the Institutional Ethics Committee (IEC) at SBISR, PHFI, PMWH, NHRC and UCL Research Ethics Committee. The study results will be disseminated globally with academic and health professionals via conferences, presentations and publications. Researchers at each study site will share emerging results with participants during their follow-up visits.

For peer review only - http://bmjopen.bmj.com/site/about/guidelines.xhtml

ARTICLE SUMMARY

Study strengths

- Large-scale, multi-centre randomised controlled, double-blind trial to determine efficacy of vitamin B-12 supplementation in improving neurodevelopmental outcomes in infancy.
- Study design benefits from preceding qualitative patient and public involvement (PPI), and nested qualitative sub-study that will ensure participant feedback and process evaluation to optimise trial conduct.
- The study is being conducted across two diverse socio-demographic populations, to increase generalisability of study findings.

Study limitations

- Different regulatory structures in two different countries.
- Normal/Routine care delivered in the two study sites differs. This could lead to differences in maternal and new-born care and monitoring between study sites.

For peer review only - http://bmjopen.bmj.com/site/about/guidelines.xhtml

1. INTRODUCTION

Malnutrition during pregnancy is a global health problem. Micronutrient deficiencies are associated with adverse developmental outcomes in children, including poorer neurocognitive functioning potentially leading to poorer educational achievement and economic prospects in affected children [1, 2].

Vitamin B-12 deficiency is prevalent across many low and middle-income countries (LMICs) [3-6]. Vegetarian diets and those low in animal food source are risk factors for B-12 deficiency [7, 8]. Sufficient B-12 intake during pregnancy is vital for foetal brain development. Deficiency of B-12 in pregnancy is linked to infant birth defects, intrauterine growth restriction, preterm delivery and neural tube defects [9-13]. Many observational studies detail the association between B-12 deficiency during pregnancy or early infancy with subsequent poorer infant neurodevelopment [14] and cognitive function [15, 16] including memory [17], language and motor skills [18].

There is limited evidence from randomised controlled trials (RCTs) regarding effect of vitamin B-12 supplementation and infant cognitive outcomes [19,20,21]. An infant supplementation trial reported neurodevelopmental benefit of infant B-12 supplementation with folate [21]. Except for vitamin B12 in relation to gross motor functioning, there was no significant benefit of giving vitamin B12 or folic acid alone on early child development. In another trial, maternal vitamin B-12 supplementation given as 50mcg a day from <14 weeks' gestation through to 6 weeks postpartum did not improve infant neurodevelopmental outcomes at 9 months [19]. In this trial hyperhomocysteinemia (a marker of B-12 insufficiency) during pregnancy was negatively associated with infant cognitive outcomes, suggesting that the dose or duration of B-12 supplementation may not have been adequate. Additionally, a high rate of participant attrition (51.4%), poor monitoring of supplement compliance, a lack of biochemical improvement in B-12 status in the supplemented group mothers and lack of infant biochemical evaluation for B-12 status in later infancy further limit the inferences that can be drawn from this trial.

For peer review only - http://bmjopen.bmj.com/site/about/guidelines.xhtml

Other RCTs that assessed a 250mcg dose of B-12 per day throughout pregnancy until 3 months' post-partum in Bangladesh [22], and ≥2000mcg from 17 to 34 weeks' gestation in India [23] did not evaluate neurodevelopment outcomes in infancy. Hence, there is insufficient evidence concerning timing, dose, duration and efficacy of B-12 supplementation. To address this evidence gap, we are conducting a double-blind multicentric RCT in one LMIC (India) and one low income country (Nepal), which will evaluate the efficacy of 250mcg/day vitamin B12 supplementation in vegetarian mothers from the first trimester to 6 months' post-partum on infant neurodevelopment outcomes measured at age 9 months. It is noteworthy that several published protocols report ongoing work on the subject from India and Nepal [24,25]. The proposed work differs significantly in several aspects from them.

We specifically chose to supplement vegetarian women as a high-risk population in view of the lower intake of B-12 and higher prevalence of deficiency. We chose the dose of 50 mcg as a quasi- control group as it has been specifically documented earlier to prevent deterioration in deficiency in Indian pregnant women. In addition, given the progressive decline in B-12 levels through pregnancy documented in the placebo group of an earlier trial and the potentially serious neonatal consequences of severe B-12 deficiency highlighted above, a pure placebo control was thought to be unethical. We chose an interventional dose of 250 mcg as it has been shown in an earlier trial to improve B-12 levels [22] although neurodevelopment was not assessed in this trial.

2. AIMS AND OBJECTIVES

Primary objective

To determine the effect of 250mcg daily oral vitamin B-12 supplementation of vegetarian pregnant and post-partum women on infant neurodevelopment as compared to 50mcg.

Secondary objectives

To determine the effect of 250mcg versus 50mcg of oral vitamin B-12 supplementation of vegetarian pregnant and post-partum women on biochemical parameters of B-12 status including:

a. Maternal - at the end of the third trimester;
b. Infant - at 9 months post-partum.

Tertiary objectives

To determine the:

a. Socio-economic mediators (income, education, profession) of the relationship between maternal B12 status, supplementation and infant neurodevelopment;

For peer review only - http://bmjopen.bmj.com/site/about/guidelines.xhtml

b. Association of maternal diet with maternal vitamin B12 status, infant B12 status and infant neurodevelopment;

c. Association between type of milk feeding on infant vitamin B12 status and infant neurodevelopment at 9 months;

d. Association of infant complementary feeding with infant vitamin B12 status and infant neurodevelopment at 9 months;

e. Influence of maternal iron and vitamin D status in first and third trimester on the relationship between infant vitamin B12 status and infant neurodevelopment at 9 months;

f. Association between maternal B12 and infant B12 status and neurodevelopment at 9 months after birth.

3. METHODOLOGY

Trial Registration:

The study is registered as a primary clinical trial at the WHO International Clinical Trials Registry Platform through the Clinical Trial Registry of India (CTRI/2018/07/015048) and at ClinicalTrials.gov (ID NCT03783104).

Setting

This study will be conducted across two sites, at Delhi, India and Kathmandu, Nepal. Both countries have documented a high prevalence of B-12 deficiency. [5,6]

For peer review only

Participants

Approximately 700 women (50-60% vegetarian) deliver annually at Sitaram Bhartia Institute of Science and Research (SBISR), New Delhi, India. SBISR is a privately funded, tertiary hospital catering to the higher and middle-income populace. Approximately 22,000 (5-10% vegetarian) women deliver annually at Paropakar Maternity and Women's Hospital (PMWH), Kathmandu, Nepal. PMWH is a government hospital facility and caters to people from poorer socio-economic strata. Data from SBISR from our earlier work (2011- 2014) documented that most of the mothers delivering at SBISR were well-educated (96.8% college education), working ladies (63.2%) living in well-earning (Mean Gross Family Income 16000 USD) nuclear families (57.9%) [26]. A directly comparable dataset was not available from PMWH.

Feasibility Data and Preliminary Work

Feasibility of data and preliminary qualitative work was conducted for the trial in 2017-18. Feasibility of recruitment within the given timelines was studied on the basis of previous data available from the study sites. The lead author (JN) examined neurodevelopmental outcome of 413 babies born at SBISR, in a cohort study conducted between 2011 and 2014. The project had a similar antenatal recruitment and post-delivery infant neurodevelopmental follow-up design as proposed in this trial [27]. Approximately 2/3 (67%) of subjects completed the cohort study. The loss to follow-up (33%) and speed of recruitment have been considered in the sample size calculations and the recruitment timeline for this trial. No similar recruitment and retention data were available from PMWH.

Patient and Public Involvement:

To represent the participants' perspective a Patient and Public Involvement (PPI) panel was established consisting of four parent pairs who had delivered a baby within the previous 2 years. One parent pair is also included in continued project development and the Trial Oversight Committee (TOC). We further conducted a qualitative study with pregnant mothers at both study sites to better understand the factors likely to influence the uptake and implementation of the proposed intervention, and proposed barriers to implementation. This qualitative study established that most respondents were unaware of B-12 dietary sources and the importance of B-12. All the participants understood the burden of the intervention and were eager to know about symptoms, treatment and prognosis of B-12 deficiency. Respondents' willingness to accept the intervention and drawing extra blood from the baby as a part of routine sampling for checking B-12 levels guided the study design. Perceived barriers including concerns around potential side effects of the B-12 supplement, relocation

For peer review only - http://bmjopen.bmj.com/site/about/guidelines.xhtml

and financial difficulties were considered in the design of patient information, consultation and follow-up procedures as follows:

- All participants presenting at the two study sites will be informed of the role and importance of vitamin B-12 in human health and its sources. This will be done through educational banners and posters and through community workshops.
- The initial information about the trial will be given by the doctor providing antenatal care to the mother. Subsequent detailed consent and recruitment will be conducted by study research staff.
- In order to improve compliance and study retention, reminder text messages are sent weekly to participating mothers.
- To incentivise women (sponsoring various biochemical tests at the Indian trial site and covering transportation cost at the Nepal site) at both sites to avoid the risk of financial stress resulting from study participation.
- To add Bradleys' Home Observation for Measurement of the Environment (HOME) inventory to the assessment given the divergent environmental profile of the two study sites [28] in order to assess the infant's environment and possible confounders of neurodevelopment.

The study results will be disseminated globally with academic and health professionals via conferences, presentations and publications. Researchers at each study site will share emerging results with participants during their follow-up visits.

We adapted a pre-validated Food Frequency Questionnaire (FFQ) for Vitamin B-12 intake developed by Gael et al. [29] to the Indian and Nepalese settings. For our purpose, the original Vitamin B12 FFQ by Gael et al. containing 30 questions on consumption of food and beverages containing B12 was reviewed independently by two nutritionists. A market survey was done to obtain information on egg and milk products in the diet as well as B-12 fortified items available in local markets of Delhi and Kathmandu. Homemade recipes which included egg and/or dairy products as a main ingredient were incorporated in the FFQ. Locally available household utensils were standardised to measure the amount of food consumed to maintain consistence across the sites.

All research staff from India and Nepal were jointly trained in study design, research methodology, good clinical practice, study protocol and study tools. Training lectures were video recorded and made available to the teams at both study sites as a reference material for continuous training.

For peer review only - http://bmjopen.bmj.com/site/about/guidelines.xhtml

For peer review only

Eligibility

Potentially eligible participants are screened at first presentation to the antenatal clinic at ≤12 weeks of gestation in accordance with the following selection criteria.

Inclusion Criteria:

- Vegetarian women defined as a self-reported dietary pattern that includes veganism and/or people who do eat egg and/or do consume milk and/or a portion meat/chicken/fish ≤ once a month;
- Living within an *a-priori* defined geographical area;
    - Delhi – national capital region;
    - Nepal – 10-km radius of PMWH;
- Able to understand at least one of English, Hindi or Nepalese.

Exclusion Criteria:

- Wish for medical termination of pregnancy;
- Aged <18 or >35 years;
- Already on medicinal vitamin B-12 supplementation including B-complex or multivitamins;
- Multiple gestation;
- Diagnosed with a chronic medical condition (including diabetes mellitus, hypertension, heart disease, neurological disease, or thyroid disease);
- Tested positive for hepatitis B, human immunodeficiency virus (HIV), or syphilis;
- Anticipate moving out of the *a-priori* defined geographical area before or after delivery;
- Treated for infertility;
- Having a known pre-diagnosed mental health disorder including depression, drug or alcohol abuse;
- Currently participating or having participated in another study within four weeks prior to trial start;
- Having a known allergy to vitamin B-12 or another supplement constituent.

Consent

Eligible participants meeting the selection criteria are given time to think over continuation of pregnancy, site of delivery, participation in the trial and any other considerations. They are contacted

For peer review only - http://bmjopen.bmj.com/site/about/guidelines.xhtml

within 48 hours by the research team about their decision and invited to provide written or video-recorded consent. Participants are able to opt into the trial any time before 12 weeks of gestation.

Randomisation

As presented in Figure 1, all eligible and consenting participants are randomised with an allocation ratio of 1:1 to either the intervention (250mcg), or control (50mcg) group. The randomisation sequence is generated at a single third-party location (non-study site) using a computer-generated sequence stratified for location. The sequence is allocated to serial numbers by a neutral person in each country using sequentially numbered, sealed, opaque envelopes. Codes within envelopes will identify boxes labelled with 5-digit codes. Each box contains enough dosages to cater to the study period of one participant. The strips containing intervention and control doses of B-12 are identical in taste, smell and appearance. All participants, recruiters, developmental therapists, laboratory staff and the data analyst are blinded to the B-12 allocation until final analysis.

Figure 1. Study design and participant flow through the study.

Procedure

At enrolment participants are invited to provide demographic details including age, height, weight, ethnicity, education and socio-economic status. Their preferred channel of subsequent communication (phone, text message, email) is also noted. Participants are allocated a study identification number and accompanied for blood sampling. The timing of blood sampling is synchronised with that of any other samples being drawn in the first trimester to minimise unnecessary venepunctures. Twelve ml of blood sample is drawn into 2 plain vials (5 ml each) and 2 ml is drawn into an EDTA vial. The EDTA sample is processed immediately for complete blood counts by coulter counter.The plain (serum separator tubes) vial samples are centrifuged at 3500 rpm for 10 minutes at the study site. The separated serum is transported on ice and stored at -70° C at round the clock temperature and custody monitored at both the study sites . Participants are given B-12 supplement doses to last five days beyond the expected date of their next visit after being fully informed as to the supplement and its administration. Within 48 hours, participants are contacted to confirm initialisation of supplementation and re-emphasise compliance.

For peer review only - http://bmjopen.bmj.com/site/about/guidelines.xhtml

Subsequent maternal visits take place monthly until 36 weeks and then weekly. At these visits, all participants visit their obstetrician as part of routine care. Participants also meet with the research staff at a pre-specified location at the study site. To maximise compliance, weekly supplement and pre-appointment reminders are sent via the preferred mode of communication. Acquired morbidity (gestational diabetes, hypertension, and hypothyroidism) and medication history are noted in case record forms (CRFs). Empty supplement packets are collected at each appointment as an indicator of adherence. In the third trimester, mothers' dietary B-12 intake is assessed using a pre-standardised and adapted FFQ and blood sampling will be repeated using the same procedures as the first trimester.

Neonatal assessment: During childbirth, a medical officer and/or paediatrician monitors the birth and post-delivery course of neonates for any morbidity potentially influencing neurodevelopment. Gestational age, growth retardation, congenital abnormalities and APGAR (appearance, pulse, grimace, activity, respiration) score is also routinely documented for neonates. Cord pH is measured for all new-borns with delayed cry and who require any intervention beyond the basic steps of resuscitation (ambu bagging, intubation, or pharmacological treatment). Any seizures, neurological problems, hypoglycaemia, hypothermia, hyperbilirubinemia are screened, diagnosed, evaluated, and managed as per institutional guidelines. As per the standard practice, all new-borns are screened for hearing deficits, vision, and critical congenital heart disease as per institutional protocols. Preterm neonates transferred to the neonatal unit are followed as per their customized follow-up and early intervention plans as recommended by the primary care provider. Neurodevelopment and biochemical assessment of preterm neonates is carried out as per their corrected ages.

Infant follow-up is conducted at 7-14 days, 4, 8, 12 and 16 weeks and at 6 and 9 months of age. At these visits, the infants undergo routine care as well as study specific evaluations (Visit Schedule: Supplementary Table 1).

Routine care: Each mother-infant dyad sees a paediatrician for age-appropriate routine care, which involves clinical evaluation, preventative care and vaccination as per standard procedure. Anthropometric measurements and signs of micronutrient deficiency (anaemia and rickets) are recorded during each visit as part of routine care. Metabolic disorders (congenital hypothyroidism, congenital adrenal hyperplasia, cystic fibrosis, biotinidase deficiency, phenylketonuria and galactosemia) are screened for and maternal support for breastfeeding by a paediatrician or lactation counsellor is provided at 7-14 days.

Visit Schedule: Supplementary Table 1

For peer review only - http://bmjopen.bmj.com/site/about/guidelines.xhtml

Study specific: Supplements are provided to mothers at each visit, with instructions for usage until 6 months after childbirth. Any morbidities and deficiencies are recorded and treated as per institutional guidelines. Maternal and infant tolerance for the supplementation including any gastrointestinal symptoms are recorded at each visit, as is maternal compliance. Provision of supplements to the mother in both groups will be stopped at 6 months post-partum.

The hospital evaluation at 9 months (±14 days) includes neurodevelopmental assessment by a developmental therapist using the Developmental Assessment Scales for Indian Infants (DASII) - an adapted and validated version of the Bayley's Scale for Infant Development Third Edition (BSID III) [30] and blood sampling for estimation of Hb, vitamin B-12, homocysteine and holo-transcobalamin concentrations. The DASII motor scale assesses control of gross and fine motor muscle group whilst cognitive, personal and social skills development is assessed through the mental scale. All neurodevelopmental assessments will be conducted by a trained developmental therapist at the institute in a soundproof room. In premature babies corrected age is used to calculate developmental quotients. Additional assessments include a complementary feeding assessment by a nutritionist using a 72-hour dietary recall and home environment assessment using the adapted Bradley HOME inventory [28] by a trained field worker.

Compliance: Research staff dispense blister packaging of ten B-12 capsules and study specific bags for participants to store, carry and keep supplements in one place. In case of loss of supplement or inability to come for follow-up, participants are able to contact the researcher at any time to request more supplements. The supplement packaging is numbered with a serial number. To maximise compliance, weekly reminders are sent through the preferred mode of communication as specified by the trial participants. Each participant is provided with a bag to save empty packaging strips and instructed to carry the bag with her at the next visit to monitor compliance.

Biochemical analysis

All saved samples will be processed as one batch after the completion of the study. The maternal samples will be used to determine the blood levels for B-12, total homocysteine, holo-transcobalamin, 25(OH)D, folate, Iron Studies (Serum Iron, ferritin and TIBC) CBC and RBC indices in the first and third trimesters. Infant blood will be used for Hb, Vitamin b-12, Homocysteine and Holo-transcobalamin measurements. Vitamin B-12 and Vitamin D will be measured by Enhanced CLIA (Johnson & Johnson, USA). Iron levels will be estimated by Pyridlazodye (Johnson & Johnson, USA). Folate will be analysed by Enhanced CLIA (Beckman Coulter, USA), and CBC by Beckman Coulter, USA. RBC indices will be

For peer review only - http://bmjopen.bmj.com/site/about/guidelines.xhtml

For peer review only

calculated by Impedance (Beckman Coulter, USA). Homocysteine and Holotranscobalamin will be analysed by Chemiluminescent Microparticle Immunoassay (CMIA).

Outcomes:

The following study outcomes will be investigated:

Primary Outcome:

- DASII scores for infants at 9 months (±2 weeks) post-partum

Secondary Outcome:

- Change in B-12 status (vitamin B12 level, homocysteine, and Holotranscobalamin) of mother between first (weeks' gestation) and third trimester ($\geq$ 27 weeks' gestation);
- B-12 status (vitamin B12 level, homocysteine, and Holotranscobalamin) of infants at 9 months (±2 weeks) post-partum.

Exploratory Outcome(s):

- Haemoglobin (Hb) of infants at 9 months ($\pm$14 days) post-partum;
- Infant anthropometry including weight, height, length and head circumference at 9 months after birth in all subjects.

Sample size considerations

A planned sample of 720 pregnant women, 360 per study site, will be randomly assigned to experimental (*n*=360; *n*=180 in per study site) or control (*n*=360; *n*=180 in per study site) groups. This is based on the number of infants needed to complete the neurodevelopmental screen in each of the two randomised groups to detect a difference in Development Quotient (DQ) at nine months (primary outcome) of 2 points (26). The difference of 2 DQ points was chosen because it is the smallest effect size thought to be clinically significant and hence a null trial result is likely to exclude a clinically relevant effect of maternal B12 supplementation on infant cognitive function. The sample size has been inflated by 35% to account for attrition and assumes 90% statistical power and a 5% two-sided significance level.

Statistical analysis

The primary and secondary outcomes are analysed using an intention-to-treat analysis by a blinded statistician. Comparability of participants assigned to study groups at enrolment is assessed using tests of association for categorical variables and by fitting generalised linear models for continuous

For peer review only - http://bmjopen.bmj.com/site/about/guidelines.xhtml

variables. These models include interaction terms to explore conditional differences between the trial's arms. If necessary, transformations are applied to the response variables to fulfil the model's assumptions. Covariates include trial site, infant feeding, gender, feeding by gender, weight *z*-score at birth, weight gain between birth and 3 months, and maternal body mass index (BMI). *Z*-scores for height and weight are calculated according to the World Health Organisation [31] growth standards using the R language for statistical computing [32].

We will examine the impact of B-12 supplementation in two subgroups – those that have any of the following conditions vs. all others (high risk vs. low risk):

- Maternal morbidity during pregnancy will be classified as high-risk (including gestational diabetes, pregnancy-induced hypertension/pre-eclampsia/eclampsia and absent or reversed end diastolic flow); and low-risk;
- Birth weight z-score categorized as high-risk (small for gestational age [SGA]: <10th centile for birth weight); and low-risk (appropriate for weight [>10th centile for birthweight]);
- Gestation categorized as preterm (low-risk; 33-37 weeks gestation); very preterm (high-risk; 28-32 weeks gestation); and term (low-risk: >37 weeks gestation). Extremely preterm (<28 weeks) will be exited;
- Higher risk neonatal course categorized as high risk (if neonate has: APGAR score <7 at 1 minute, hypoglycaemia, hyperbilirubinemia or prolonged neonatal intensive care stay >7 days); and low risk.
- Infants with documented neurological disease/anomaly/illness (Developmental Quotient (by DASII scale of the infant at 9 months) will be sub-grouped separately (high risk) and compared to those with no disease/anomaly illness (low risk).

Data management and dissemination

Data will be collected under the regulation of the data protection and management guidelines given by Governments of India and Nepal, and as per the UK Data Protection Act 2018. Data will be securely stored in an E-database with password protection. The E-database was designed using Access. Live data entry will be conducted at the study sites and tested for accuracy using duplicate data entry at an offsite location at periodic intervals.

The study results will be disseminated globally with academic and health professionals via conferences, presentations and publications. Researchers at each study site will share emerging results with participants during their follow-up visits. Regular site meetings with press offices, short

For peer review only

films, radio spots, periodic blog posts, Twitter and Facebook messages, and online articles are also planned to strengthen the trial's online presence and reach young and middle-aged internet users for whom the information may be of interest.

Qualitative evaluation proposed:

The qualitative sub-study aims to explore knowledge, attitudes and perceptions of pregnant women towards B-12 supplementation and child development, and potential barriers to implementation. Better understanding these domains across the two study sites may inform the perceived need for the intervention, help define patient-centric outcomes and allow the evaluation of any differences between the two arms.

We will use convenience sampling and estimate interviewing 30 participants across both trial sites. We will develop and pilot a semi-structured topic guide in collaboration with our PPI representatives serving on the Trial Steering Committee (TSC). The topic guide will be designed in English and translated into either Hindi or Nepali for administration across the study sites. The topic guide will then be back translated to English to ensure consistency and accuracy of content. It is intended to help direct the interview whilst remaining sensitive to unsolicited themes. An interviewer with prior training and experience in qualitative methods will facilitate the semi-structured interviews.

Data will be collected in quiet rooms in either hospital, away from other patients and staff. Interviews will be facilitated in English, Hindi or Nepalese. All interviews will be audio recorded, transcribed, translated into English (where necessary) and then crosschecked against the original recording. The translated transcripts will then be coded using the Atlas Ti 7.2 software for qualitative analysis. [33] A research team member will code and categorise data, and prepare an initial narrative. A final report will then be prepared with input from the interviewers and wider research team.

Ethical considerations:

The Study Protocol (10th December 2018. Version: 8.0), Informed Consent Form, Participant Information Sheet, Tool and other supportive materials have been approved by the Institutional Ethics Committee (IEC) at SBISR (FL/SBISR/IEC/2018-01); PHFI (TRC-IEC-380/18); PMWH (59-11-433); Nepal Health Research Council Ethical Review Board (NHRC) (Reg No. 761/2018) and the University College London (UCL) Research Ethics Committee (10901/001). The Investigators at each study site has submitted and, where necessary, obtained approval from the above parties for all substantial amendments to the original approved documents.

The trial is being conducted in accordance to the principles and rules of the Declaration of Helsinki. Informed and written consent is obtained from all participants by the research teams at each study

For peer review only - http://bmjopen.bmj.com/site/about/guidelines.xhtml

site. The trial is complying with the Data Protection Act (UK), 2018 ensuring the participants' anonymity. The participants are identified only by a participant identification number on all trial documents and electronic databases. Every effort is made to arrange interviews and examination at a time convenient to the participants without causing much disruption in their daily routine. Study data is stored securely and is only accessible to trial staff and authorised personnel. The Principal Investigator at each study site will submit an annual progress report, end of trial notification and a final report to the Institutional Ethics Committee, host organisations and the funders.

We used the SPIRIT checklist for writing our protocol [34].

DISCUSSION

This study will have considerable impact on current and future policy initiatives focusing on micronutrient supplementation in India and Nepal by underpinning micronutrient supplementation policy in India as envisaged in the recently released "National Health Policy", and the micronutrient initiative in Nepal. The project will provide key insights to strengthen existing national nutrition programmes like "National Iron Plus Initiative", "Janini Suraksha Yojna" and "Janini Shishu Suraksha Kariyakram" initiated by the Government of India and similar initiatives by the Government of Nepal. Because of the high worldwide prevalence of maternal B-12 deficiency (74%), the study findings will be highly relevant for maternal and neonatal health in nutritionally vulnerable and at-risk populations globally.

As one of the few large-scale trials of B-12 supplementation in pregnancy, the results of the study would be generalisable to vegetarian expecting mothers; in whom deficiency of Vitamin B12 is most prevalent in South Asia. a key aim of the project is to reset significant elements of the research agenda in maternal and child health. We aim to contribute to the evidence base on improving neurodevelopmental outcomes in vulnerable infants. While a positive outcome for the trial would help develop an intervention program to reduce developmental delay using Vitamin B-12 as a supplementation in pregnant women, a negative or null result would also add to existing knowledge and lay the groundwork for future work on the subject especially around utility, dose, duration and time of initiation of Vitamin B12 supplementation in pregnant women/infants.

DECLARATIONS

Acknowledgements: CL and ML were partly supported by the National Institute for Health Research (NIHR) Collaborations for Leadership in Applied Health Research and Care (CLAHRC) North Thames at the time of this work. We would also like to thank our Patient and Public Involvement (PPI) panel.

For peer review only - http://bmjopen.bmj.com/site/about/guidelines.xhtml

Author Statements: JN, MM, and ML conceived the study and AS, MH, MCB, JG, RP contributed to the study design. SR and DN conducted the qualitative study, adaptation of FFQ, standardisation of questionnaires and organised the training workshop. SR, DN, KA and CL drafted the manuscript. JN, MM and ML finalised the manuscript, and all authors assisted in developing the protocol and have read and approved the final manuscript.

Funding: The study is funded by the Newton Fund: The Department of Biotechnology (DBT) India, in collaboration with Department of International Development (DFID), the Economic and Social Research Council (ESRC) and the Medical Research Council (MRC) consortium (Grant Number MR/R020396/1). This research was supported by the National Institute for Health Research (NIHR) Biomedical Research Centre based at UCL Great Ormond Street Institute of Child Health/Great Ormond Street Hospital NHS Foundation Trust.

Competing interests: All authors declare no competing interests.

Provenance and peer review: Not commissioned; externally peer reviewed

Data sharing statement: For access to the data set, a formal request should be sent to the MATCOBIND study group. The request will only be considered when the results of the study have been published.

For peer review only

REFERENCES

1. Vohr BR, Poggi Davis E, Wanke CA, Krebs NF. Neurodevelopment: The Impact of Nutrition and Inflammation During Preconception and Pregnancy in Low-Resource Settings. Pediatrics. 2017;139(Suppl 1):S38–S49.
2. Victora CG, Adair L, Fall C, et al. Maternal and child undernutrition: consequences for adult health and human capital. Lancet. 2008;371(9609):340–57.
3. Allen, LH. Folate and vitamin B12 status in the Americas. Nutr Rev. 2004 Jun; 62(6): S29-33.
4. Allen, LH. Causes of vitamin B12 and folate deficiency. Food Nutr Bull. 2008 Jun;29(2):20-34.
5. Ulak M, Chandyo RK, Adhikari RK, et al. Cobalamin and folate status in 6 to 35 months old children presenting with acute diarrhea in Bhaktapur, Nepal. PLoS One 2014;9: e90079.
6. Taneja S, Bhandari N, Strand TA, Sommerfelt H, Refsum H, Ueland PM, Scheede J, Bahl R, Bhan MK. Cobalamin and folate status in infants and young children in a low-to-middle income community in India. Am J Clin Nutr. 2007; 86:1302–1309.
7. Sklar R. Nutritional vitamin B12 deficiency in a breast-fed infant of a vegan-diet mother. Clin Pediatr (Phila). 1986;25(4):219–21.
8. Kuhne T, Bubl R, Baumgartner R. Maternal vegan diet causing a serious infantile neurological disorder due to vitamin B12 deficiency. Eur J Pediatr. 1991;150(3):205-208.
9. Lee C, Maza, Stern B, Lakhanpaul M, Parikh P. Associations between the household environment and stunted child growth in rural India: a cross-sectional analysis. UCL Open: Environment. 2019: doi: 10.14324/111.444/000015.v1.
10. Ganguly P, Alam SF. Role of homocysteine in the development of cardiovascular disease. *Nutr J*. 2015; 14:6. 2015 Jan 10. doi:10.1186/1475-2891-14-6.
11. Black MM. Effects of Vitamin B12 and Folate Deficiency on Brain Development in Children. Food Nutr Bull. 2008;29(2): S126–31.
12. Molloy AM, Kirke PN, Troendle JF, Burke H, Sutton M, Brody LC, Scott JM, Mills JL. Maternal vitamin B12 status and risk of neural tube defects in a population with high neural tube defect prevalence and no folic acid fortification. Pediatrics. 2009; 123:917–923.
13. Refsum H. Folate, vitamin B12 and homocysteine in relation to birth defects and pregnancy outcome. Br J Nutr. 2001;85: S109–S113.
14. Azad C, Jat KR, Kaur J, Guglani V, Palta A, Tiwari A, Bansal D. Vitamin B12 status and neurodevelopmental delay in Indian infants: a hospital-based cross-sectional study. Paediatrics and international child health. 2019 Jul 4:1-7.

For peer review only - http://bmjopen.bmj.com/site/about/guidelines.xhtml

15. Siddiqua JT. Vitamin B12 deficiency in pregnancy and lactation: is there a need for pre and post-natal supplementation? J Nutr Disord Ther. 2014;4(2):1-8.
16. Dror DK, Allen LH. Effect of vitamin B12 deficiency on neurodevelopment in infants: current knowledge and possible mechanism. Nutr Rev. 2008; 66:250-255.
17. Bhate V, Deshpande S, Bhat D, Joshi N, Ladkat R, Watve S, et al. Vitamin B12 status of pregnant Indian women and cognitive function in their 9-year-old children. Food Nutr Bull. 2008; 29:249– 254.
18. Torsvik I, Ueland PM, Markestad T, Bjørke-Monsen AL. Cobalamin supplementation improves motor development and regurgitations in infants: results from a randomized intervention study. The American journal of clinical nutrition. 2013 Sep 11;98(5):1233-40.
19. Duggan C, Srinivasan K, Thomas T, Samuel T, Rajendran R, Muthayya S, Finkelstein JL, Lukose A, Fawzi W, Allen LH, Bosch RJ. Vitamin B-12 supplementation during pregnancy and early lactation increases maternal, breast milk, and infant measures of vitamin B-12 status. The Journal of nutrition. 2014 Mar 5;144(5):758-64.
20. Srinivasan K, Thomas T, Kapanee ARM, Ramthal A, Bellinger DC, Bosch RJ et al. Effects of maternal vitamin B12 supplementation on early infant neurocognitive outcomes: a randomised controlled clinical trial. Matern Child Nutr. 2016: doi:10.1111/mcn/12345.
21. Kvestad I, Taneja S, Kumar T, Hysing M, Refsum H, Yajnik CS, Bhandari N, Strand TA, Folate and Vitamin B12 Study Group. Vitamin B12 and folic acid improve gross motor and problem-solving skills in young North Indian children: a randomized placebo-controlled trial. Plos one. 2015 Jun 22;10(6):e0129915.
22. Siddiqua TJ, Ahmad SM, Ahsan SB, Rashid M, Roy A, Rahman SM et al. Vitamin B12 supplementation during pregnancy and postpartum improves B12 status of both mothers and infants but vaccine response in mothers only: a randomised control in Bangladesh. Eur J Nutr. 2016; 55(1)281-293.
23. Katre P, Bhat D, Lubree H, Otiv S, Joshi S, Joglekar C et al. Vitamin B12 and folic acid supplementation and plasma total homocysteine concentrations in pregnant women India women with low B12 and high folate status. Asia Pac J Clin Nutr. 2010;19(3):335-43.
24. Winje BA, Kvestad I, Krishnamachari S, Manji K, Taneja S, Bellinger DC, Bhandari N, Bisht S, Darling AM, Duggan CP, Fawzi W. Does early vitamin B12 supplementation improve neurodevelopment and cognitive function in childhood and into school age: a study protocol for extended follow-ups from randomised controlled trials in India and Tanzania. BMJ open. 2018 Feb 1;8(2):e018962.
25. Strand TA, Ulak M, Chandyo RK, Kvestad I, Hysing M, Shrestha M, Basnet S, Ranjitkar S, Shrestha L, Shrestha PS. The effect of vitamin B 12 supplementation in Nepalese infants on

For peer review only - http://bmjopen.bmj.com/site/about/guidelines.xhtml

growth and development: study protocol for a randomized controlled trial. Trials. 2017 Dec;18(1):187.

26. Nagpal, J., Sachdeva, A., Sengupta Dhar, R., Bhargava, V., & Bhartia, A. Widespread non-adherence to evidence-based maternity care guidelines: a population-based cluster randomised household survey. BJOG: An International Journal of Obstetrics & Gynaecology. 2014; 122(2), 238–247.doi:10.1111/1471-0528.13054.
27. Nagpal J, Bhargava VL, Sengupta R, Dhall S, Agarwal N, Gulati K. A prospective cohort study to evaluate the utility of antenatal doppler velocimetry in prediction of fetal malnutrition, neonatal morbidity, mortality and neurodevelopmental outcome: Final report submitted to ICMR 2015. Downloaded from url: http://14.139.60.56/ICMRFINALREPORT/SQL_FR_ABS_PDF_Disp.aspx?Type=FR&Proj_No=2009-01550&SLNO=1
28. Bradley R.H., Caldwell B.M., Rock S.L.,Ramey C.T.,Barnard K.E., Gray C. et al. Home environment and cognitive development in the first 3 years of life: a collaborative study involving six sites and three ethnic groups in North America. Developmental Psychology. 1989; 25 (2),217.
29. Mearns GJ, Rush EC. Screening for inadequate dietary vitamin B12 intake in South Asian women using a nutrient-specific, semi-quantitative food frequency questionnaire. Asia Pacific journal of clinical nutrition. 2016 Nov 18.
30. Patni B. Developmental Assessment Scales for Indian Infants (DASII). Indian J Pract Pediatr. 2012;14(4):409–12.
31. WHO Multicentre Growth Reference Study Group. WHO Child Growth Standards based on length/height, weight and age. Acta paediatrica (Oslo, Norway: 1992). Supplement. 2006 Apr; 450:76.
32. R Core Team (2013). R: A language and environment for statistical computing. R Foundation for Statistical Computing, Vienna, Austria. Available via: //www.R-project.org.
33. ATLAS.ti: The Qualitative Data Analysis; Research Software.2019.
34. Chan A-W, Tetzlaff JM, Altman DG, Laupacis A, Gøtzsche PC, Krleža-Jerić K, Hróbjartsson A, Mann H, Dickersin K, Berlin J, Doré C, Parulekar W, Summerskill W, Groves T, Schulz K, Sox H, Rockhold FW, Rennie D, Moher D. SPIRIT 2013 Statement: Defining standard protocol items for clinical trials. Ann Intern Med. 2013;158(3):200-207."

For peer review only

For peer review only - http://bmjopen.bmj.com/site/about/guidelines.xhtml

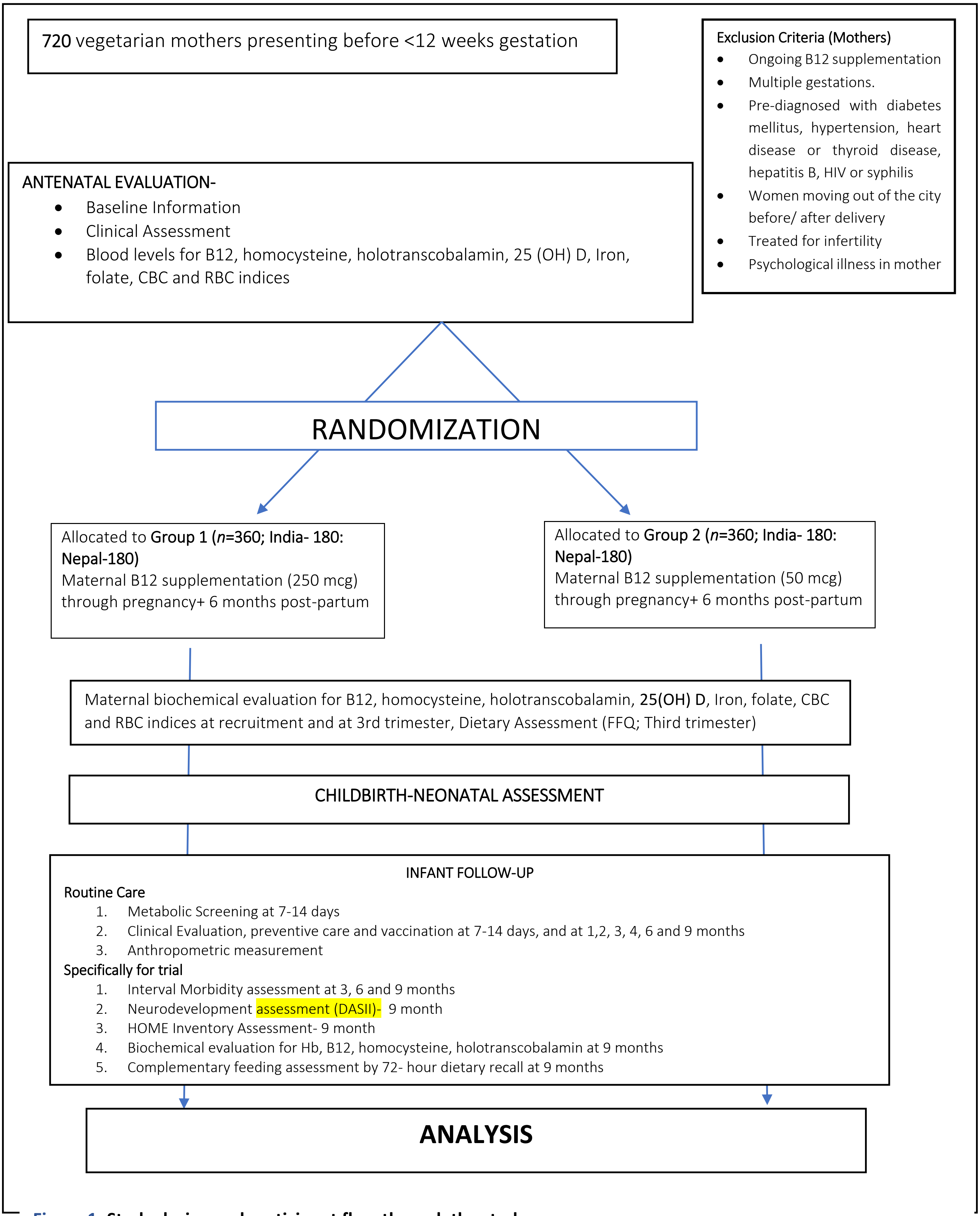

Figure 1. Study design and participant flow through the study.

For peer review only

Supplementary Material: Table 1. Visit schedule

**Study Plan**	**Initial Contact (<12 weeks gestation)**		**M1**	**M2-M9**	**M10**	**At Birth**	**I1**	**I2-I6**	**I7**
**Schedule**	**Screening**	**Recruitment**	**Antenatal Follow-up**	**Antenatal Follow-up (monthly until second trimester, fortnightly in the third trimester**	**Antenatal Follow-up (weekly after 36 weeks until delivery)**		**Infant 7-14 day**	**Infant follow-up: At months- 1 (± 1 wk), 2 (± 2 wks), 3 (± 2 wks), 4 (± 2 wks) & 6 (± 2 wks)**	**Infant follow-up Age 9 months ± 14 days;**
First Screening Visit	X								
Maternal Inclusion/Exclusion criteria	X	X							
Informed consent of the participant		X							
Questionnaire Administration		X							
Randomization		X							
Supplementation		X	X	X	X	X	X	X	
Blood Sampling (1st trimester)		X							
Home Visit (if required)			X	X	X	X	X	X	X
Monitoring of the Compliance			X	X	X	X	X	X	X
Adverse events			X	X	X	X	X	X	X
Blood Sampling (3rd trimester, >26 weeks gestation)				X					
Mother Food Frequency Questionnaire (>26 weeks gestation)				X					
Infant Anthropometry (Weight/length/Head Circumference)						X	X	X	X
Monitoring for neonatal morbidity*						X	X	X	X
Application of Exit Criteria (specified earlier)			X	X	X	X	X	X	X
Metabolic Screening of infant							X		
Infant morbidity							X	X	X
Infant blood sampling									X
Complementary feeding 72 hr. Dietary Recall									X
DASII Scale									X

For peer review only

HOME Inventory									X

For peer review only

Reporting checklist for protocol of a clinical trial.

Based on the SPIRIT guidelines.

Instructions to authors

Complete this checklist by entering the page numbers from your manuscript where readers will find each of the items listed below.

Your article may not currently address all the items on the checklist. Please modify your text to include the missing information. If you are certain that an item does not apply, please write "n/a" and provide a short explanation.

Upload your completed checklist as an extra file when you submit to a journal.

In your methods section, say that you used the SPIRITreporting guidelines, and cite them as:

Chan A-W, Tetzlaff JM, Altman DG, Laupacis A, Gøtzsche PC, Krleža-Jerić K, Hróbjartsson A, Mann H, Dickersin K, Berlin J, Doré C, Parulekar W, Summerskill W, Groves T, Schulz K, Sox H, Rockhold FW, Rennie D, Moher D. SPIRIT 2013 Statement: Defining standard protocol items for clinical trials. Ann Intern Med. 2013;158(3):200-207

		Reporting Item	Page Number
**Administrative information**			
Title	#1	Descriptive title identifying the study design, population, interventions, and, if applicable, trial acronym	1

For peer review only

Trial registration	#2a	Trial identifier and registry name. If not yet registered, name of intended registry	5
Trial registration: data set	#2b	All items from the World Health Organization Trial Registration Data Set	5
Protocol version	#3	Date and version identifier	14
Funding	#4	Sources and types of financial, material, and other support	15
Roles and responsibilities: contributorship	#5a	Names, affiliations, and roles of protocol contributors	1
Roles and responsibilities: sponsor contact information	#5b	Name and contact information for the trial sponsor	15
Roles and responsibilities: sponsor and funder	#5c	Role of study sponsor and funders, if any, in study design; collection, management, analysis, and interpretation of data; writing of the report; and the decision to submit the report for publication, including whether they will have ultimate authority over any of these activities	15
Roles and responsibilities: committees	#5d	Composition, roles, and responsibilities of the coordinating centre, steering committee, endpoint adjudication committee, data management team, and other individuals or groups overseeing the trial, if applicable (see Item 21a for data monitoring committee)	

For peer review only

Introduction

Background and rationale	#6a	Description of research question and justification for undertaking the trial, including summary of relevant studies (published and unpublished) examining benefits and harms for each intervention	4
Background and rationale: choice of comparators	#6b	Explanation for choice of comparators	4
Objectives	#7	Specific objectives or hypotheses	4
Trial design	#8	Description of trial design including type of trial (eg, parallel group, crossover, factorial, single group), allocation ratio, and framework (eg, superiority, equivalence, non-inferiority, exploratory)	

Methods: Participants, interventions, and outcomes

Study setting	#9	Description of study settings (eg, community clinic, academic hospital) and list of countries where data will be collected. Reference to where list of study sites can be obtained	5
Eligibility criteria	#10	Inclusion and exclusion criteria for participants. If applicable, eligibility criteria for study centres and	7

For peer review only

		individuals who will perform the interventions (eg, surgeons, psychotherapists)	
Interventions: description	#11a	Interventions for each group with sufficient detail to allow replication, including how and when they will be administered	9
Interventions: modifications	#11b	Criteria for discontinuing or modifying allocated interventions for a given trial participant (eg, drug dose change in response to harms, participant request, or improving / worsening disease)	
Interventions: adherance	#11c	Strategies to improve adherence to intervention protocols, and any procedures for monitoring adherence (eg, drug tablet return; laboratory tests)	11
Interventions: concomitant care	#11d	Relevant concomitant care and interventions that are permitted or prohibited during the trial	10-11
Outcomes	#12	Primary, secondary, and other outcomes, including the specific measurement variable (eg, systolic blood pressure), analysis metric (eg, change from baseline, final value, time to event), method of aggregation (eg, median, proportion), and time point for each outcome. Explanation of the clinical relevance of chosen efficacy and harm outcomes is strongly recommended	11
Participant timeline	#13	Time schedule of enrolment, interventions (including any run-ins and washouts), assessments, and visits for participants. A schematic diagram is highly recommended	

For peer review only

		(see Figure)	
Sample size	#14	Estimated number of participants needed to achieve study objectives and how it was determined, including clinical and statistical assumptions supporting any sample size calculations	11
Recruitment	#15	Strategies for achieving adequate participant enrolment to reach target sample size	
**Methods: Assignment of interventions (for controlled trials)**			
Allocation: sequence generation	#16a	Method of generating the allocation sequence (eg, computer-generated random numbers), and list of any factors for stratification. To reduce predictability of a random sequence, details of any planned restriction (eg, blocking) should be provided in a separate document that is unavailable to those who enrol participants or assign interventions	8
Allocation concealment mechanism	#16b	Mechanism of implementing the allocation sequence (eg, central telephone; sequentially numbered, opaque, sealed envelopes), describing any steps to conceal the sequence until interventions are assigned	8-9
Allocation: implementation	#16c	Who will generate the allocation sequence, who will enrol participants, and who will assign participants to interventions	8

For peer review only

Blinding (masking)	#17a	Who will be blinded after assignment to interventions (eg, trial participants, care providers, outcome assessors, data analysts), and how	8
Blinding (masking): emergency unblinding	#17b	If blinded, circumstances under which unblinding is permissible, and procedure for revealing a participant's allocated intervention during the trial	
**Methods: Data collection, management, and analysis**			
Data collection plan	#18a	Plans for assessment and collection of outcome, baseline, and other trial data, including any related processes to promote data quality (eg, duplicate measurements, training of assessors) and a description of study instruments (eg, questionnaires, laboratory tests) along with their reliability and validity, if known. Reference to where data collection forms can be found, if not in the protocol	12
Data collection plan: retention	#18b	Plans to promote participant retention and complete follow-up, including list of any outcome data to be collected for participants who discontinue or deviate from intervention protocols	13
Data management	#19	Plans for data entry, coding, security, and storage, including any related processes to promote data quality (eg, double data entry; range checks for data values).	13

For peer review only

		Reference to where details of data management procedures can be found, if not in the protocol	
Statistics: outcomes	#20a	Statistical methods for analysing primary and secondary outcomes. Reference to where other details of the statistical analysis plan can be found, if not in the protocol	12-13
Statistics: additional analyses	#20b	Methods for any additional analyses (eg, subgroup and adjusted analyses)	
Statistics: analysis population and missing data	#20c	Definition of analysis population relating to protocol non-adherence (eg, as randomised analysis), and any statistical methods to handle missing data (eg, multiple imputation)	
**Methods: Monitoring**			
Data monitoring: formal committee	#21a	Composition of data monitoring committee (DMC); summary of its role and reporting structure; statement of whether it is independent from the sponsor and competing interests; and reference to where further details about its charter can be found, if not in the protocol. Alternatively, an explanation of why a DMC is not needed	
Data monitoring: interim analysis	#21b	Description of any interim analyses and stopping guidelines, including who will have access to these interim results and make the final decision to terminate the trial	
Harms	#22	Plans for collecting, assessing, reporting, and managing solicited and spontaneously reported adverse events and other unintended effects of trial interventions or trial	

For peer review only

		conduct	
Auditing	#23	Frequency and procedures for auditing trial conduct, if any, and whether the process will be independent from investigators and the sponsor	
**Ethics and dissemination**			
Research ethics approval	#24	Plans for seeking research ethics committee / institutional review board (REC / IRB) approval	14-15
Protocol amendments	#25	Plans for communicating important protocol modifications (eg, changes to eligibility criteria, outcomes, analyses) to relevant parties (eg, investigators, REC / IRBs, trial participants, trial registries, journals, regulators)	14-15
Consent or assent	#26a	Who will obtain informed consent or assent from potential trial participants or authorised surrogates, and how (see Item 32)	14-15
Consent or assent: ancillary studies	#26b	Additional consent provisions for collection and use of participant data and biological specimens in ancillary studies, if applicable	14-15
Confidentiality	#27	How personal information about potential and enrolled participants will be collected, shared, and maintained in order to protect confidentiality before, during, and after the trial	14-15
Declaration of	#28	Financial and other competing interests for principal	16

For peer review only

interests		investigators for the overall trial and each study site	
Data access	#29	Statement of who will have access to the final trial dataset, and disclosure of contractual agreements that limit such access for investigators	13-14
Ancillary and post trial care	#30	Provisions, if any, for ancillary and post-trial care, and for compensation to those who suffer harm from trial participation	
Dissemination policy: trial results	#31a	Plans for investigators and sponsor to communicate trial results to participants, healthcare professionals, the public, and other relevant groups (eg, via publication, reporting in results databases, or other data sharing arrangements), including any publication restrictions	13
Dissemination policy: authorship	#31b	Authorship eligibility guidelines and any intended use of professional writers	X
Dissemination policy: reproducible research	#31c	Plans, if any, for granting public access to the full protocol, participant-level dataset, and statistical code	
**Appendices**			
Informed consent materials	#32	Model consent form and other related documentation given to participants and authorised surrogates	X
Biological specimens	#33	Plans for collection, laboratory evaluation, and storage of biological specimens for genetic or molecular analysis in the current trial and for future use in ancillary studies, if applicable	X

For peer review only

None The SPIRIT checklist is distributed under the terms of the Creative Commons Attribution License CC-BY-ND 3.0. This checklist can be completed online using https://www.goodreports.org/, a tool made by the EQUATOR Network in collaboration with Penelope.ai

For peer review only

BMJ Open

Efficacy of maternal B-12 supplementation in vegetarian women for improving infant neurodevelopment: Protocol for the MATCOBIND multicentre, double-blind, randomised controlled trial

Journal:	*BMJ Open*
Manuscript ID	bmjopen-2019-034987.R2
Article Type:	Protocol
Date Submitted by the Author:	27-Feb-2020
Complete List of Authors:	Nagpal, Jitender; Sitaram Bhartia Institute of Science and Research, Pediatrics<br>Mathur, Manu; Public Health Foundation of India,<br>Rawat , Swapnil ; Sitaram Bhartia Institute of Science and Research, Pediatrics<br>Nagrath, Deepti; Public Health Foundation of India,<br>Lee, Charlotte; University College London, Institute of Child Health<br>Singhal, Atul ; University College London Institute of Child Health<br>Heys, Michelle; Specialist Children's and Young People's Services, East London NHS Foundation Trust<br>Cortina Borja, Mario; UCL, Institute of Child Health<br>Augustin, Katrin ; University College London, Institute of Child Health<br>Gautam , Jageshwor; Paropakar Maternity & Women's Hospital<br>Pant , Rajendra; Paropakar Maternity & Women's Hospital<br>Lakhanpaul, Monica; University College London, Institute of Child Health
**Primary Subject Heading**:	Nutrition and metabolism
Secondary Subject Heading:	Paediatrics, Obstetrics and gynaecology
Keywords:	Maternal medicine < OBSTETRICS, PAEDIATRICS, NUTRITION & DIETETICS, Developmental neurology & neurodisability < PAEDIATRICS, Community child health < PAEDIATRICS

SCHOLARONE™
Manuscripts

I, the Submitting Author has the right to grant and does grant on behalf of all authors of the Work (as defined in the below author licence), an exclusive licence and/or a non-exclusive licence for contributions from authors who are: i) UK Crown employees; ii) where BMJ has agreed a CC-BY licence shall apply, and/or iii) in accordance with the terms applicable for US Federal Government officers or employees acting as part of their official duties; on a worldwide, perpetual, irrevocable, royalty-free basis to BMJ Publishing Group Ltd ("BMJ") its licensees and where the relevant Journal is co-owned by BMJ to the co-owners of the Journal, to publish the Work in this journal and any other BMJ products and to exploit all rights, as set out in our licence.

The Submitting Author accepts and understands that any supply made under these terms is made by BMJ to the Submitting Author unless you are acting as an employee on behalf of your employer or a postgraduate student of an affiliated institution which is paying any applicable article publishing charge ("APC") for Open Access articles. Where the Submitting Author wishes to make the Work available on an Open Access basis (and intends to pay the relevant APC), the terms of reuse of such Open Access shall be governed by a Creative Commons licence – details of these licences and which Creative Commons licence will apply to this Work are set out in our licence referred to above.

Other than as permitted in any relevant BMJ Author's Self Archiving Policies, I confirm this Work has not been accepted for publication elsewhere, is not being considered for publication elsewhere and does not duplicate material already published. I confirm all authors consent to publication of this Work and authorise the granting of this licence.

For peer review only

TITLE PAGE

Title

Efficacy of maternal B-12 supplementation in vegetarian women for improving infant neurodevelopment: Protocol for the MATCOBIND multicentre, double-blind, randomised controlled trial

Running Head: Maternal Vit-B12 and Infant Neurodevelopment Protocol

Authors

Jitender Nagpal[a], Manu Raj Mathur[b], Swapnil Rawat[a], Deepti Nagrath[b], Charlotte Lee[c], Atul Singhal[c], Michelle Heys[d], Mario Cortina-Borja[c], Katrin Augustin[c],Jageshwor Gautam[e], Rajendra Pant[e], Monica Lakhanpaul[c*]

a. *Sitaram Bhartia Institute of Science and Research (SBISR), B16, Qutab Institutional Area, New Delhi, 110016, India. jitendernagpal@gmail.com; swapnil.rawat@gmail.com*
b. *Public Health Foundation of India (PHFI)* Plot Number 47, Sector 44, Institutional Area, Gurugram – 122002, India. manu.mathur@phfi.org ; deepti.nagrath@phfi.org
c. *UCL Great Ormond Street Institute of Child Health, University College London, 30 Guildford Street, London, WC1N 1EH, United Kingdom. a.singhal@ucl.ac.uk; m.cortina@ucl.ac.uk; k.augustin@ucl.ac.uk; c.lee.12@ucl.ac.uk; m.lakhanpaul@ucl.ac.uk* .
d. *Specialist Children's and Young People's Services, East London NHS Foundation Trust, London, UK; m.heys@ucl.ac.uk*.
e. *Paropakar Maternity and Womens' Hospital (PMWH), Thapathali, Kathmandu, Nepal.* drjgautam48@gmail.com; *rajendrapant8@gmail.com* .

*Corresponding Author

Professor Monica Lakhanpaul; UCL Great Ormond Street Institute of Child Health, University College London, 30 Guildford Street, London, WC1N 1EH, United Kingdom; m.lakhanpaul@ucl.ac.uk .

Word Count

Abstract: 250

Main text: 4899

For peer review only - http://bmjopen.bmj.com/site/about/guidelines.xhtml

Tables: 1

Figures: 1

Key Words- Maternal Health, Vitamin B-12, Infant Neurodevelopment

ABSTRACT

Introduction: Vitamin B-12 deficiency is widely prevalent across many low and middle-income countries, especially where the diet is low in animal sources food. While many observational studies show associations between B-12 deficiency in pregnancy and infant cognitive function, (including memory, language and motor skills), evidence from clinical trials is sparse and inconclusive.

Methods and Analysis: This double blind, multi-centre randomised controlled trial will enrol 720 vegetarian pregnant women in the first trimester from ante-natal clinics at two hospitals, one in India and one in Nepal). Eligible mothers who give written consent will be randomised to receive either 250 mcg methylcobalamin or 50 mcg (quasi control) from enrolment to 6 months post-partum, given as an oral daily capsule. All mothers and their infants will continue to receive standard clinical care. The primary trial outcome is the offspring's neurodevelopment status at 9 months of age, assessed using the Development Assessment Scale of Indian Infants. Secondary outcomes include the infant's biochemical B-12 status at age 9 months and maternal biochemical B-12 status in the first and third trimesters. Maternal biochemical B-12 status will also be assessed in the first trimester. Modification of association by apriori identified factors will also be explored.

Ethical Considerations and Dissemination: The Study Protocol has been approved by ethical committees at each site and at University College London. The study results will be disseminated to healthcare professional and academics globally via conferences, presentations and publications. Researchers at each study site will share results with participants during their follow-up visits.

For peer review only - http://bmjopen.bmj.com/site/about/guidelines.xhtml

ARTICLE SUMMARY

Study strengths

- Large-scale, multi-centre randomised controlled, double-blind trial across a low-middle income country and low-income country to determine efficacy of maternal vitamin B-12 supplementation during pregnancy on neurodevelopmental outcomes six-month post-partum.
- Study design benefits from preceding qualitative patient and public involvement (PPI), and nested qualitative sub-study that will ensure participant feedback and process evaluation to optimise trial conduct.
- The study is being conducted across two diverse socio-demographic populations, to increase generalisability of study findings.

Study limitations

- Normal/Routine care differs at the two sites, which could lead to differences in the conduct and monitoring of the trial.
- Health system regulatory structures differ in the two countries.

For peer review only

For peer review only - http://bmjopen.bmj.com/site/about/guidelines.xhtml

1. INTRODUCTION

Malnutrition during pregnancy is a global health problem. Micronutrient deficiencies are associated with adverse developmental outcomes in children, including poorer neurocognitive functioning which can potentially lead to poorer educational achievement and economic prospects [1, 2].

Vitamin B-12 deficiency is prevalent across many low and middle-income countries (LMICs) [3-6]. Partaking in a vegetarian diet, or one low in animal food sources, is a key risk factor in developing B-12 deficiency [7, 8]. Sufficient B-12 intake during pregnancy is vital for foetal brain development [9]. Deficiency of B-12 in pregnancy is linked to infant birth defects, intrauterine growth restriction, preterm delivery and neural tube defects [10-14]. Many observational studies have shown associations between B-12 deficiency during pregnancy or early infancy with subsequent poorer infant neurodevelopment [15] and cognitive function [16, 17] including memory [18], language and motor skills [19].

There is limited evidence from randomised controlled trials (RCTs) regarding effect of vitamin B-12 supplementation and infant cognitive outcomes [20,21,22]. An infant supplementation trial observed neurodevelopmental benefit of infant B-12 supplementation with folate and documented 0.45 (95% CI 0.19, 0.73) and 0.28 (95% CI 0.02, 0.54) higher SD-units in the domains of gross motor and problem-solving functioning, respectively [22]. However, in this study there was no significant benefit of giving vitamin B-12 or folic acid alone on early child development (with the exception of vitamin B-12 in relation to gross motor functioning [22]). In another trial, maternal vitamin B-12 supplementation given as 50mcg a day from <14 weeks' gestation through to 6 weeks postpartum did not improve infant neurodevelopmental outcomes at 9 months [21]. In this trial hyperhomocysteinemia (a marker of B-12 insufficiency) during pregnancy was negatively associated with infant cognitive outcomes. The study documented a deterioration in biochemical B-12 status in the placebo group while the rise in B-12 status in the supplemented group (50 mcg) was inconsistent (no significant difference between first and third trimester levels). Additionally, a high rate of participant attrition (51.4%), poor monitoring of supplement compliance, a lack of biochemical improvement in B-12 status in the supplemented group mothers and lack of infant biochemical evaluation for B-12 status in later infancy further limit the inferences that can be drawn from this trial. Other studies that assessed a 250mcg dose of B-12 per day throughout pregnancy until 3 months' post-partum in Bangladesh [23], and >2000mcg from 17 to 34 weeks' gestation in India [24] did not evaluate neurodevelopment outcomes in infancy. Hence, there is insufficient evidence concerning timing, dose, duration and efficacy of B-12 supplementation with reference to infant neurodevelopment.

For peer review only - http://bmjopen.bmj.com/site/about/guidelines.xhtml

To address this evidence gap, we are conducting a double-blind multi-centre RCT in one Low- and Middle-Income Country (LMIC; India) and one Low- Income Country(LIC; Nepal), which will evaluate the efficacy of 250mcg/day vitamin B-12 supplementation in vegetarian mothers from the first trimester to 6 months' post-partum on infant neurodevelopmental outcomes at age 9 months. Although, several published protocols report ongoing work on B-12 supplementation from India and Nepal [25,26], the proposed work differs significantly in several aspects.

First, we specifically chose to supplement vegetarian women, as they are a high-risk population in view of their low B-12 intake and high prevalence of B-12 deficiency. Second, an interventional dose of 250 mcg was chosen because it has been shown in an earlier trial to improve B-12 levels in pregnancy [23] although infant neurodevelopment was not assessed in this trial. Third, we chose 50 mcg B-12 /day as a quasi- control group as this dose has been shown to prevent deterioration in B-12 status during pregnancy in India [21,22]. Furthermore, given the progressive decline in B-12 levels through pregnancy (documented in the placebo group of earlier trials [21,22]), and the potentially serious neonatal consequences of severe B-12 deficiency highlighted above, a pure placebo control was thought to be unethical.

2. AIMS AND OBJECTIVES

Primary objective

To determine the effect of 250mcg versus 50mcg daily oral vitamin B-12 supplementation of pregnant women from the first trimester to 6 months post-partum on infant neurodevelopment at age 9 months (specifically the impact on the Motor and Mental developmental quotient (DQ) by Development Assessment Scale for Indian Infants (DASII scale).

Secondary objectives

To determine the effect of 250mcg versus 50mcg of oral vitamin B-12 supplementation in pregnancy and 6 months post-partum on biochemical parameters of B-12 status (Vitamin B-12 levels, Homocysteine and Holo-transcobalamin) in mothers at the end of the third trimester and in infants at age 9 months.

Tertiary objectives

1. To evaluate the influence of the following effect modifiers:
    a. Socio-economic indicators (income, education, profession) on the relationship between maternal B12 status, supplementation and infant neurodevelopment;

For peer review only - http://bmjopen.bmj.com/site/about/guidelines.xhtml

b. Type of milk feeding on the infant vitamin B-12 status and infant neurodevelopment at 9 months;

c. Maternal dietary B-12 intake with maternal and infant B-12 status, supplementation and infant neurodevelopment;

d. Infant complementary feeding with infant vitamin B-12 status and infant neurodevelopment at 9 months;

e. Maternal and infant B-12 status and neurodevelopment at 9 months after birth.

2. To determine the interaction of maternal iron and vitamin D status with the relationship between infant vitamin B-12 status and infant neurodevelopment at 9 months.

For peer review only

3. METHODOLOGY

Trial Registration:

The study is registered as a primary clinical trial at the WHO International Clinical Trials Registry Platform through the Clinical Trial Registry of India (CTRI/2018/07/015048) and at ClinicalTrials.gov (ID NCT04083560).

Setting

This study includes two sites: Delhi, India and Kathmandu, Nepal. Both countries have documented a high prevalence of B-12 deficiency [5,6].

For peer review only - http://bmjopen.bmj.com/site/about/guidelines.xhtml

Participants

Mothers will be recruited from the 1) Sitaram Bhartia Institute of Science and Research (SBISR), New Delhi, India, a privately- funded, tertiary hospital catering for a high and middle-income population, with approximately 700 deliveries annually (50-60% mothers are vegetarian); and 2) Paropakar Maternity and Women's Hospital (PMWH), Kathmandu, Nepal, a government hospital catering for a poorer socio-economic area with approximately 22,000 deliveries annually (5-10% mothers are vegetarian). Our earlier work (2011- 2014) documented that the majority of mothers delivering at SBISR were well-educated (96.8% college education), employed (63.2%) living in high income (annual income 16,000 USD) nuclear families (57.9%) [27]. A directly comparable dataset was not available from PMWH.

Feasibility Data and Preliminary Work

Feasibility of data and preliminary qualitative work was conducted for the trial in 2017-18. Feasibility of recruitment within the given timelines was studied on the basis of previous data available from the study sites. The lead author (JN) examined neurodevelopmental outcomes of 413 babies born at SBISR in a cohort study conducted between 2011 and 2014. The project had a similar design, using antenatal recruitment and post-delivery infant neurodevelopmental follow-up as proposed in this trial [27]. Approximately 2/3 (67%) of subjects were retained to follow-up in the cohort study. The loss to follow-up (33%) and speed of recruitment have been considered in the sample size calculations and the recruitment timeline for this trial. No similar recruitment and retention data were available from PMWH.

Patient and Public Involvement:

To represent the participants' perspective, a Patient and Public Involvement (PPI) panel was established consisting of four parent pairs (2 pairs per site) who had delivered a baby within the previous 2 years. One parent pair has continued to be involved with the project development and the Trial Oversight Committee (TOC). We conducted an exploratory study with pregnant mothers at both study sites to better understand the factors likely to influence the uptake and implementation of the proposed intervention, and proposed barriers to implementation (data unpublished). This qualitative study established that most respondents were unaware of B-12 dietary sources and the importance of B-12. All the participants understood the burden of the intervention and were eager to know about symptoms, treatment and prognosis of B-12 deficiency. Respondents' were also willing to accept the intervention and the need for drawing extra blood from the baby as a part of routine sampling for B-

For peer review only - http://bmjopen.bmj.com/site/about/guidelines.xhtml

12 levels. Perceived barriers to the study included concerns around potential side effects of the B-12 supplement, relocation and financial difficulties. All of these preceding factors were considered in the study design, development of patient information, consultation and follow-up procedures as follows:

- All participants at the two study sites will be informed of the role, sources and importance of vitamin B-12 in human health using educational banners and posters and through community workshops.
- The initial information about the trial will be given by the doctor providing antenatal care to the mother. Subsequent detailed consent and recruitment will be conducted by study research staff.
- In order to improve compliance and study retention, reminder text messages will be sent weekly to participating mothers.
- To avoid the risk of financial loss to participants, various biochemical tests at the Indian trial site will be sponsored, and transportation costs at the Nepal site will be covered.
- Bradleys' Home Observation for Measurement of the Environment (HOME) inventory was added to the assessment given the divergent environmental profile of the two study sites [28], in order to assess the infant's environment and possible environmental confounders of neurodevelopment.

The study results will be disseminated globally to academics and health professionals via conferences, presentations and publications. Researchers at each study site will share results with participants during their follow-up visits.

Also, in preparation for this trial we adapted a pre-validated Food Frequency Questionnaire (FFQ) for Vitamin B-12 intake developed by Gael et al. [29] to the Indian and Nepalese settings. In brief, the original Vitamin B-12 FFQ by Gael et al. (containing 30 questions on consumption of food and beverages containing B-12) was reviewed independently by two nutritionists. A market survey was done to obtain information on egg and milk products in the diet as well as B-12 fortified items available in local markets of Delhi and Kathmandu. Homemade recipes which included egg and/or dairy products as a main ingredient were incorporated in the FFQ. Measurements used in the FFQ were standardised based on the typical/locally available household utensils to ensure accurate responses and to maintain consistency across the sites.

All research staff from India and Nepal will be jointly trained in study design, research methodology, good clinical practice, the study protocol and study tools. Training lectures will be video-recorded and made available to the teams at both study sites as a reference material for continuous training.

For peer review only - http://bmjopen.bmj.com/site/about/guidelines.xhtml

Eligibility

Potentially eligible participants will be screened at first presentation to the antenatal clinic at ≤12 weeks of gestation in accordance with the following criteria:

Inclusion Criteria:

- Consuming a vegetarian diet -including veganism and/or people who do eat egg and/or do consume milk and/or a portion meat/chicken/fish ≤ once a month;
- Living within an *a-priori* defined geographical area;
    - Delhi – national capital region;
    - Nepal – 10-km radius of PMWH;
- Able to understand at least one of English, Hindi or Nepalese.

Exclusion Criteria:

- Wish for medical termination of pregnancy;
- Aged <18 or >35 years;
- Already on medicinal vitamin B-12 supplementation including B-complex or multivitamins;
- Multiple gestation;
- Diagnosis of a chronic medical condition (including diabetes mellitus, hypertension, heart disease, neurological disease, or thyroid disease);
- Testing positive for hepatitis B, human immunodeficiency virus (HIV), or syphilis;
- Anticipating moving out of the *a-priori* defined geographical area before or after delivery;
- Undergoing treatment for infertility;
- Having a known pre-diagnosed mental health disorder, including depression, drug or alcohol abuse;
- Currently participating or having participated in another study within four weeks prior to the trial commencing;
- Having a known allergy to vitamin B-12 or another supplement constituent.

Consent

Eligible participants meeting the selection criteria will be given time to think over continuation of pregnancy, site of delivery, participation in the trial and any other considerations. They will be contacted within 48 hours by the research team regarding their decision to participate or not and will

For peer review only - http://bmjopen.bmj.com/site/about/guidelines.xhtml

be invited to provide written or video-recorded consent if relevant. Participants will be able to opt into the trial any time before 12 weeks of gestation.

Randomisation

As presented in Figure 1, all eligible and consenting participants will be randomised with an allocation ratio of 1:1 to either the intervention (250mcg), or control (50mcg) group. The randomisation sequence will be generated at a single, third-party location (non-study site) using a computer-generated sequence stratified for location. The sequence will be allocated to serial numbers by a neutral person in each country using sequentially numbered, sealed, opaque envelopes. Codes within envelopes will identify boxes labelled with 5-digit codes. Each box will contain enough dosages to cater to the study period of one participant. The strips containing intervention and control doses of B-12 will be identical in taste, smell and appearance. All participants, recruiters, developmental therapists, laboratory staff and the data analyst will be blinded to the B-12 allocation until results of final analysis are made available.

Figure 1. Study design and participant flow through the study.

Procedure

Upon enrolment, demographic and clinical details, including age, height, weight, ethnicity, education and socio-economic status, will be collected. The participants' preferred channel of subsequent communication (phone, text message, email) will be noted. Participants will be allocated a study identification number and accompanied by the researcher for blood sampling. The timing of blood sampling will be synchronised with that of any other samples being drawn in the first trimester to minimise unnecessary venepunctures. Twelve ml of blood sample will be drawn into two plain vials (five ml each) and two ml will be drawn into an EDTA vial. The EDTA sample will be processed immediately for complete blood counts by coulter counter. The plain (serum separator tubes) vial samples will be centrifuged at 3500 rpm (Rotor radius 15 cm) for 10 minutes at the study site. The separated serum will be transported on ice and consistently stored at -70° C and will be monitored at both the study sites. Participants will be given enough B-12 supplement doses to last five days beyond the expected date of their next visit, after being fully informed about the supplement and its

For peer review only - http://bmjopen.bmj.com/site/about/guidelines.xhtml

administration. Participants will be contacted 48 hours after receiving the B-12 doses to confirm initialisation of supplementation and re-emphasise compliance.

Subsequent maternal visits will take place monthly until 36 weeks, and then weekly thereafter. At these visits, all participants will visit their obstetrician as part of routine care. Participants will also meet with the research staff at a pre-specified location at the study site. To maximise compliance, weekly supplement and pre-appointment reminders will be sent via the pre-specified preferred mode of communication. Acquired morbidity (gestational diabetes, hypertension, and hypothyroidism) and medication history will be noted in case record forms (CRFs). Empty supplement packets will be collected at each appointment as an indicator of adherence. In the third trimester, mothers' dietary B-12 intake will be assessed using a pre-standardised and adapted FFQ, and blood sampling will be repeated using the same procedures as in the first trimester.

Neonatal assessment: During childbirth, a medical officer and/or paediatrician will monitor the birth and post-delivery course of neonates for any morbidity that might potentially influence neurodevelopment. Gestational age, growth retardation, congenital abnormalities and APGAR (appearance, pulse, grimace, activity, respiration) score will be routinely documented for neonates. Cord pH will be measured for all new-borns with a delayed cry and who require any intervention beyond the basic steps of resuscitation (ambu bagging, intubation, or pharmacological treatment). Any seizures, neurological problems, hypoglycaemia, hypothermia, or hyperbilirubinemia will be assessed, diagnosed, evaluated, and managed as per institutional guidelines. As per standard practice, all new-borns will be screened for hearing deficits, vision, and critical congenital heart disease according to institutional protocols. Preterm neonates transferred to the neonatal unit will be monitored as per their customized follow-up and early intervention plans, implemented as recommended by the paediatrics and/or primary care provider. Neurodevelopment and biochemical assessment of preterm neonates will be carried out as per their corrected gestational ages.

Infant follow-up will be conducted at 7-14 days, 4, 8, 12 and 16 weeks and at 6 and 9 months of age. At these visits, the infants will undergo routine care as well as study specific evaluations (see Visit Schedule: Supplementary Table 1).

Routine care: Each mother-infant dyad will see a paediatrician for age-appropriate routine care, which will involve clinical evaluation, preventative care and vaccination as per standard procedures in each site. Anthropometric measurements and signs of micronutrient deficiency (anaemia and rickets) will

For peer review only - http://bmjopen.bmj.com/site/about/guidelines.xhtml

be recorded during each visit as part of routine care. Metabolic disorders (congenital hypothyroidism, congenital adrenal hyperplasia, cystic fibrosis, biotinidase deficiency, phenylketonuria and galactosemia) will be screened for and maternal support for breastfeeding by a paediatrician or lactation counsellor will be provided at 7-14 days.

Study specific: Supplements will be provided to mothers at each visit, with instructions to take these until 6 months after childbirth. Any morbidities and deficiencies will be recorded and treated as per institutional guidelines. Maternal and infant tolerance for the supplementation including any gastrointestinal symptoms will be recorded at each visit, as will maternal compliance. Provision of supplements to the mother in both groups will be stopped at 6 months post-partum.

The hospital evaluation at 9 months (±14 days) will include neurodevelopmental assessment by a developmental therapist using the Developmental Assessment Scales for Indian Infants (DASII) - an adapted and validated version of the Bayley's Scale for Infant Development Third Edition (BSID II) [30] and blood sampling for estimation of Hb, vitamin B-12, homocysteine and Holo-transcobalamin concentrations. The DASII motor scale assesses gross and fine motor skills, whilst cognitive, personal and social skills development are assessed through the DASII mental scale. All neurodevelopmental assessments will be conducted by a trained developmental therapist at the institute in a soundproof room. In premature babies, corrected gestational age will be used to calculate developmental quotients. Additional assessments will include a complementary feeding assessment by a nutritionist using a 72-hour dietary recall and home environment assessment using the adapted Bradley HOME inventory [28] by a trained field worker.

Compliance: Research staff will dispense blister packaging of ten B-12 capsules and study specific bags for participants to store, carry and keep supplements in one place. In case of loss of supplement or inability to come for follow-up, participants will be able to contact the researcher at any time to request more supplements. The supplement packaging will be numbered with a serial number. To maximise compliance, weekly reminders will be sent through the preferred mode of communication as specified by the trial participants. Each participant will be provided with a bag to save empty packaging strips and instructed to carry the bag with her at the next visit to monitor compliance.

Biochemical analysis

All saved samples will be processed as one batch after the completion of the study. The maternal samples will be used to determine the blood levels for Vitamin B-12, Homocysteine, Holo-

For peer review only - http://bmjopen.bmj.com/site/about/guidelines.xhtml

For peer review only

transcobalamin, 25(OH)D, folate, Iron Studies (Serum Iron, ferritin and Total Iron Binding Capacity), Complete Blood Count (CBC) and Red Blood Cell (RBC) indices in the first and third trimesters. Infant blood will be used for Haemoglobin, Vitamin B-12, Homocysteine and Holo-transcobalamin measurements. Vitamin B-12 and 25(OH)D will be measured by Enhanced CLIA (Johnson & Johnson, USA). Iron levels will be estimated by Pyridyl azodye (Johnson & Johnson, USA). Folate will be analysed by Enhanced Chemiluminescence Immunoassay (CLIA) (Beckman Coulter, USA), and CBC by Beckman Coulter, USA. RBC indices will be calculated by Impedance (Beckman Coulter, USA). Homocysteine and Holo-transcobalamin will be analysed by Chemiluminescent Microparticle Immunoassay (CMIA).

Outcomes:

The following study outcomes will be investigated:

Primary Outcome:

- DASII scores for infants at 9 months (±2 weeks) post-partum.

Secondary Outcome (s):

- Change in B-12 status (vitamin B-12 level, Homocysteine, and Holo-transcobalamin) of mother between first (<u><</u>12 weeks' gestation) and third trimester (<u>></u> 27 weeks' gestation);
- B-12 status (vitamin B-12 level, Homocysteine, and Holo-transcobalamin) of infants at 9 months (±2 weeks) post-partum.

Exploratory Outcome(s):

- Haemoglobin (Hb) of infants at 9 months (±14 days) post-partum;
- Infant anthropometry including weight, height/ length and head circumference at 9 months after birth in all subjects.

Sample size considerations

A planned sample of 720 pregnant women, 360 per study site, will be randomly assigned to experimental (*n*=360; *n*=180 in per study site) or control (*n*=360; *n*=180 in per study site) groups. This is based on the number of infants needed to complete the neurodevelopmental screen in each of the two randomised groups to detect a difference in Development Quotient (DQ) at nine months (primary outcome) of 2 points (presumed mean DQ in this population of infants at 9 months of 96.9, SD 7.07 on basis of earlier work)([27]. The difference of 2 DQ points was chosen because it is the smallest

For peer review only - http://bmjopen.bmj.com/site/about/guidelines.xhtml
For peer review only

effect size thought to be clinically significant and hence a null trial result is likely to exclude a clinically relevant effect of maternal B12 supplementation on infant cognitive function. The sample size has been inflated by 35% to account for attrition, and assumes 90% statistical power and a 5% two-sided significance level.

Statistical analysis

The primary and secondary outcomes will be analysed using an intention-to-treat analysis by a blinded statistician. Comparability of participants assigned to study groups at enrolment will be assessed using tests of association for categorical variables and by fitting generalised linear models for continuous variables. These models will include interaction terms to explore conditional differences between the trial's arms. If necessary, transformations will be applied to the response variables to fulfil the model's assumptions. Covariates will include trial site, infant feeding, gender, feeding by gender, weight *z*-score at birth, weight gain between birth and 3 months, and maternal body mass index (BMI). *Z*-scores for height and weight will be calculated according to the World Health Organisation [31] growth standards using the R language for statistical computing [32].

Subgroup analysis will be conducted to examine the differential impact of B-12 supplementation in high versus low risk mothers and infants. Mothers identified as having pregnancy related or other morbidity will be classified as high-risk (e.g. including gestational diabetes, pregnancy-induced hypertension/pre-eclampsia/eclampsia and absent or reversed end diastolic flow); and low-risk. Babies will be defined as high risk if they are any of a combination of the following:

- Small for gestational age [SGA], defined as <10th centile for birth weight;
  - Very preterm (28-32 weeks gestation); Extremely preterm babies (<28 weeks) will be excluded from the analysis;
  - Experience of a higher risk neonatal course defined as experience of APGAR score <7 at 1 minute, hypoglycaemia, hyperbilirubinemia or prolonged neonatal intensive care stay >7 days);
  - Infants with documented neurological disease/anomaly/illness will be sub-grouped separately (high risk) and compared to those with no disease/anomaly illness (low risk).

Data management and dissemination

Data will be collected under the regulation of the data protection and management guidelines given by Governments of India and Nepal, and as per the UK Data Protection Act 2018. Data will be securely

For peer review only - http://bmjopen.bmj.com/site/about/guidelines.xhtml

stored in an E-database with password protection. The E-database will be designed using Access. Live data entry will be conducted at the study sites and tested for accuracy using duplicate data entry at an offsite location at periodic intervals.

The study results will be disseminated globally with academic and health professionals via conferences, presentations and publications. Researchers at each study site will share emerging results with participants during their follow-up visits. Regular site meetings with press offices, short films, radio spots, periodic blog posts, Twitter and Facebook messages, and online articles are also planned to strengthen the trial's online presence and reach young and middle-aged internet users for whom the information may be of interest.

Qualitative evaluation proposed

A qualitative sub-study aims to explore knowledge, attitudes and perceptions of pregnant women towards B-12 supplementation and child development, and potential barriers to implementation. Better understanding these domains across the two study sites may inform the perceived need for the intervention, help define patient-centric outcomes and allow the evaluation of any differences between the two arms.

We will use convenience sampling, and aim to interview 30 participants across both trial sites. We will develop and pilot a semi-structured topic guide in collaboration with our PPI representatives serving on the Trial Steering Committee (TSC). The topic guide will be designed in English and translated into either Hindi or Nepali. The topic guide will then be back-translated to English to ensure consistency and accuracy of content. It is intended to help direct the interview whilst remaining sensitive to unsolicited themes. An interviewer with prior training and experience in qualitative methods will facilitate the semi-structured interviews.

Data will be collected in quiet rooms in each hospital, away from other patients and staff. Interviews will be facilitated in English, Hindi or Nepalese, depending on the preference of the participant. All interviews will be audio recorded, transcribed, translated into English (where necessary) and then crosschecked against the original recording. The translated transcripts will then be coded using the Atlas Ti 7.2 software for qualitative analysis [33]. A research team member will code and categorise data, and prepare an initial narrative. A final report will then be prepared with input from the interviewers and wider research team.

Ethical considerations:

The Study Protocol (10th December 2018. Version: 8.0), Informed Consent Form, Participant Information Sheet, Tool and other supportive materials have been approved by the Institutional Ethics

For peer review only - http://bmjopen.bmj.com/site/about/guidelines.xhtml

Committee (IEC) at SBISR (FL/SBISR/IEC/2018-01); PHFI (TRC-IEC-380/18); PMWH (59-11-433); Nepal Health Research Council Ethical Review Board (NHRC) (Reg No. 761/2018) and the University College London (UCL) Research Ethics Committee (10901/001). The Investigators at each study site has submitted and, where necessary, obtained approval from the above parties for all substantial amendments to the original approved documents.

The trial will be conducted in accordance to the principles and rules of the Declaration of Helsinki. Informed and written consent will be obtained from all participants by the research teams at each study site. The trial will comply with the Data Protection Act (UK), 2018, ensuring the participants' anonymity. The participants will be identified only by a participant identification number on all trial documents and electronic databases. Every effort will be made to arrange interviews and examination at a time convenient to the participants, without causing much disruption in their daily routine. Study data will be stored securely and will only be accessible to trial staff and authorised personnel. The Principal Investigator at each study site will submit an annual progress report, end of trial notification and a final report to the Institutional Ethics Committee, host organisations and the funders.

We used the SPIRIT checklist for writing our protocol [34].

DISCUSSION

A positive result from this study will have considerable impact on current and future policy initiatives focusing on micronutrient supplementation in India and Nepal, by underpinning micronutrient supplementation policy in India as envisaged in the recently released "National Health Policy", and the micronutrient initiative in Nepal. The project will provide key insights to strengthen existing national nutrition programmes like "National Iron Plus Initiative", "Janini Suraksha Yojna" and "Janini Shishu Suraksha Kariyakram" initiated by the Government of India and similar initiatives by the Government of Nepal. Because of the high worldwide prevalence of maternal B-12 deficiency (74%), the study findings will also be highly relevant for maternal and neonatal health in nutritionally vulnerable and at-risk populations globally.

Using a 50 mcg quasi-control group limits the inferences that can be drawn from a negative result (neurodevelopment comparable between the two groups). However, the following interpretations might be possible:

1. *Findings:* Improvement in B-12 parameters from first trimester to third trimester is statistically significant in both groups and the change is statistically comparable between two groups for mother and baby. Substantial proportion of mother/infants in both groups improve from B-12 deficient to replete state.

For peer review only - http://bmjopen.bmj.com/site/about/guidelines.xhtml

For peer review only

Interpretation: 250 mcg dose is as effective as 50 mcg in improving biochemical parameters and both doses result in comparable neurodevelopment. 250 mcg dosing offers no additional advantage over 50 mcg.

2. *Findings:* Improvement in B-12 parameters from first trimester to third trimester is significant in either 250 mcg alone or both groups, but 250 mcg causes higher rise (statistically significant) in B-12 biochemical parameters than 50 mcg for mother and baby.

   Interpretation: 250 mcg is more efficacious than 50 mcg in improving biochemical B-12 status. Since neurodevelopment is equivalent between two groups a higher dose of supplementation does not offer any neurodevelopmental advantage despite better biochemical B-12 status.

3. *Findings:* A lack of biochemical improvement in both groups, although unexpected, would be interpreted to mean poor efficacy of 50 mcg or 250 mcg oral B-12 in improving B-12 status of mother-infant pairs. Both groups continue to have a high proportion of mothers deficient in B-12 before and after supplementation.

   Interpretation: Factors affecting B-12 status in the current trial (including compliance and socio-demographics) would have to carefully analysed. Further work would be required to study the determinants of oral B-12 efficacy in detail.

Many studies have evaluated the impact of varying Vitamin B-12 intake (physiological (2 mcg/d) to pharmacological (up to 2000 mcg/d); supplemented/observed) in diverse population sets [20-24, 35]. Our study specifically seeks to address the question of optimum dose for vegetarian pregnant women. As one of the few large-scale trials of B-12 supplementation in pregnancy, the results of this study would be generalisable to vegetarian expecting mothers. We aim to contribute to the evidence base on improving neurodevelopmental outcomes in vulnerable infants. While a positive outcome for the trial would help to develop an intervention program to reduce developmental delay using Vitamin B-12 as a supplementation in pregnant women, a negative or null result would also add to existing knowledge and lay the groundwork for future work on the subject especially around utility, dose, duration and time of initiation of Vitamin B-12 supplementation in pregnant women/infants.

DECLARATIONS

Acknowledgements: CL and ML were partly supported by the National Institute for Health Research (NIHR) Collaborations for Leadership in Applied Health Research and Care (CLAHRC) North Thames at the time of this work. We would also like to thank our Patient and Public Involvement (PPI) panel. MH is funded by East London NHS Foundation Trust.

For peer review only - http://bmjopen.bmj.com/site/about/guidelines.xhtml

Author Statements: JN, MM, and ML conceived the study and AS, MH, MCB, JG, RP, ML, CL contributed to the study design. SR and DN conducted the qualitative study, adaptation of FFQ, standardisation of questionnaires and organised the training workshop. SR, DN, KA and CL drafted the manuscript. JN, MM and ML finalised the manuscript, and all authors assisted in developing the protocol and have read, reviewed, edited and approved the final manuscript.

Funding: The study is funded by the Newton Fund: The Department of Biotechnology (DBT) India, in collaboration with Department of International Development (DFID), the Economic and Social Research Council (ESRC) and the Medical Research Council (MRC) consortium (Grant Number MR/R020396/1). This research was supported by the National Institute for Health Research (NIHR) Biomedical Research Centre based at UCL Great Ormond Street Institute of Child Health/Great Ormond Street Hospital NHS Foundation Trust.

Competing interests: All authors declare no competing interests.

Provenance and peer review: Not commissioned; externally peer reviewed

Data sharing statement: For access to the data set, a formal request should be sent to the MATCOBIND study group at manu.mathur@phfi.org. The request will only be considered when the results of the study have been published.

For peer review only

REFERENCES

1. Vohr BR, Poggi Davis E, Wanke CA, Krebs NF. Neurodevelopment: The Impact of Nutrition and Inflammation During Preconception and Pregnancy in Low-Resource Settings. Paediatrics. 2017;139(Suppl 1): S38–S49.
2. Victora CG, Adair L, Fall C, et al. Maternal and child undernutrition: consequences for adult health and human capital. Lancet. 2008;371(9609):340–57.
3. Allen, LH. Folate and vitamin B12 status in the Americas. Nutr Rev. 2004 Jun; 62(6): S29-33.
4. Allen, LH. Causes of vitamin B12 and folate deficiency. Food Nutr Bull. 2008 Jun;29(2):20-34.
5. Ulak M, Chandyo RK, Adhikari RK, et al. Cobalamin and folate status in 6 to 35 months old children presenting with acute diarrhea in Bhaktapur, Nepal. PLoS One 2014;9: e90079.
6. Taneja S, Bhandari N, Strand TA, Sommerfelt H, Refsum H, Ueland PM, Scheede J, Bahl R, Bhan MK. Cobalamin and folate status in infants and young children in a low-to-middle income community in India. Am J Clin Nutr. 2007; 86:1302–1309.
7. Sklar R. Nutritional vitamin B12 deficiency in a breast-fed infant of a vegan-diet mother. Clin Pediatr (Phila). 1986;25(4):219–21.
8. Kuhne T, Bubl R, Baumgartner R. Maternal vegan diet causing a serious infantile neurological disorder due to vitamin B12 deficiency. Eur J Pediatr. 1991;150(3):205-208.
9. Pepper MR, Black MM. B12 in foetal development. *Semin Cell Dev Biol*. 2011;22(6):619–623. doi: 10.1016/j.semcdb.2011.05.005
10. Lee C, Maza, Stern B, Lakhanpaul M, Parikh P. Associations between the household environment and stunted child growth in rural India: a cross-sectional analysis. UCL Open: Environment. 2019: doi: 10.14324/111.444/000015.v1.
11. Ganguly P, Alam SF. Role of homocysteine in the development of cardiovascular disease. *Nutr J*. 2015; 14:6. 2015 Jan 10. doi:10.1186/1475-2891-14-6.
12. Black MM. Effects of Vitamin B12 and Folate Deficiency on Brain Development in Children. Food Nutr Bull. 2008;29(2): S126–31.
13. Molloy AM, Kirke PN, Troendle JF, Burke H, Sutton M, Brody LC, Scott JM, Mills JL. Maternal vitamin B12 status and risk of neural tube defects in a population with high neural tube defect prevalence and no folic acid fortification. Pediatrics. 2009; 123:917–923.
14. Refsum H. Folate, vitamin B12 and homocysteine in relation to birth defects and pregnancy outcome. Br J Nutr. 2001;85: S109–S113.
15. Del Río Garcia C, Torres-Sánchez L, Chen J, Schnaas L, Hernández C, Osorio E, Portillo MG, López-Carrillo L. Maternal MTHFR 677C> T genotype and dietary intake of folate and vitamin

For peer review only - http://bmjopen.bmj.com/site/about/guidelines.xhtml

B12: their impact on child neurodevelopment. Nutritional neuroscience. 2009 Feb 1;12(1):13-20.

16. Siddiqua JT. Vitamin B12 deficiency in pregnancy and lactation: is there a need for pre and post-natal supplementation? J Nutr Disord Ther. 2014;4(2):1-8.
17. Dror DK, Allen LH. Effect of vitamin B12 deficiency on neurodevelopment in infants: current knowledge and possible mechanism. Nutr Rev. 2008; 66:250-255.
18. Bhate V, Deshpande S, Bhat D, Joshi N, Ladkat R, Watve S, et al. Vitamin B12 status of pregnant Indian women and cognitive function in their 9-year-old children. Food Nutr Bull. 2008; 29:249– 254.
19. Torsvik I, Ueland PM, Markestad T, Bjørke-Monsen AL. Cobalamin supplementation improves motor development and regurgitations in infants: results from a randomized intervention study. The American journal of clinical nutrition. 2013 Sep 11;98(5):1233-40.
20. Duggan C, Srinivasan K, Thomas T, Samuel T, Rajendran R, Muthayya S, Finkelstein JL, Lukose A, Fawzi W, Allen LH, Bosch RJ. Vitamin B-12 supplementation during pregnancy and early lactation increases maternal, breast milk, and infant measures of vitamin B-12 status. The Journal of nutrition. 2014 Mar 5;144(5):758-64.
21. Srinivasan K, Thomas T, Kapanee ARM, Ramthal A, Bellinger DC, Bosch RJ et al. Effects of maternal vitamin B12 supplementation on early infant neurocognitive outcomes: a randomised controlled clinical trial. Matern Child Nutr. 2016: doi:10.1111/mcn/12345.
22. Kvestad I, Taneja S, Kumar T, Hysing M, Refsum H, Yajnik CS, Bhandari N, Strand TA, Folate and Vitamin B12 Study Group. Vitamin B12 and folic acid improve gross motor and problem-solving skills in young North Indian children: a randomized placebo-controlled trial. Plos one. 2015 Jun 22;10(6):e0129915.
23. Siddiqua TJ, Ahmad SM, Ahsan SB, Rashid M, Roy A, Rahman SM et al. Vitamin B12 supplementation during pregnancy and postpartum improves B12 status of both mothers and infants but vaccine response in mothers only: a randomised control in Bangladesh. Eur J Nutr. 2016; 55(1)281-293.
24. Katre P, Bhat D, Lubree H, Otiv S, Joshi S, Joglekar C et al. Vitamin B12 and folic acid supplementation and plasma total homocysteine concentrations in pregnant women India women with low B12 and high folate status. Asia Pac J Clin Nutr. 2010;19(3):335-43.
25. Winje BA, Kvestad I, Krishnamachari S, Manji K, Taneja S, Bellinger DC, Bhandari N, Bisht S, Darling AM, Duggan CP, Fawzi W. Does early vitamin B12 supplementation improve neurodevelopment and cognitive function in childhood and into school age: a study protocol

For peer review only - http://bmjopen.bmj.com/site/about/guidelines.xhtml

for extended follow-ups from randomised controlled trials in India and Tanzania. BMJ open. 2018 Feb 1;8(2):e018962.

26. Strand TA, Ulak M, Chandyo RK, Kvestad I, Hysing M, Shrestha M, Basnet S, Ranjitkar S, Shrestha L, Shrestha PS. The effect of vitamin B 12 supplementation in Nepalese infants on growth and development: study protocol for a randomized controlled trial. Trials. 2017 Dec;18(1):187.
27. Nagpal J, Bhargava VL, Sengupta R, Dhall S, Agarwal N, Gulati K. A prospective cohort study to evaluate the utility of antenatal doppler velocimetry in prediction of foetal malnutrition, neonatal morbidity, mortality and neurodevelopmental outcome: Final report submitted to ICMR 2015. Downloaded from url: http://14.139.60.56/ICMRFINALREPORT/SQL_FR_ABS_PDF_Disp.aspx?Type=FR&Proj_No=2009-01550&SLNO=1
28. Bradley RH, Caldwell BM, Rock SL,Ramey CT,Barnard KE, Gray C et al. Home environment and cognitive development in the first 3 years of life: a collaborative study involving six sites and three ethnic groups in North America. Developmental Psychology. 1989; 25 (2),217.
29. Mearns GJ, Rush EC. Screening for inadequate dietary vitamin B12 intake in South Asian women using a nutrient-specific, semi-quantitative food frequency questionnaire. Asia Pacific journal of clinical nutrition. 2016 Nov 18.
30. Patni B. Developmental Assessment Scales for Indian Infants (DASII). Indian J Pract Pediatr. 2012;14(4):409–12.
31. World Health Organization Multicentre Growth Reference Study Group. World Health Organization Child Growth Standards based on length/height, weight and age. Acta paediatrica (Oslo, Norway: 1992). Supplement. 2006 Apr; 450:76.
32. R Core Team (2013). R: A language and environment for statistical computing. R Foundation for Statistical Computing, Vienna, Austria. Available via: //www.R-project.org.
33. ATLAS.Ti: The Qualitative Data Analysis; Research Software.2019.
34. Chan A-W, Tetzlaff JM, Altman DG, Laupacis A, Gøtzsche PC, Krleža-Jerić K, Hróbjartsson A, Mann H, Dickersin K, Berlin J, Doré C, Parulekar W, Summerskill W, Groves T, Schulz K, Sox H, Rockhold FW, Rennie D, Moher D. SPIRIT 2013 Statement: Defining standard protocol items for clinical trials. Ann Intern Med. 2013;158(3):200-207.
35. Yajnik CS, Behere RV, Bhat DS, Memane N, Raut D, Ladkat R, Yajnik PC, Kumaran K, Fall CH. A physiological dose of oral vitamin B-12 improves hematological, biochemical-metabolic indices and peripheral nerve function in B-12 deficient Indian adolescent women. PloS one. 2019;14(10).

For peer review only

For peer review only - http://bmjopen.bmj.com/site/about/guidelines.xhtml

For peer review only

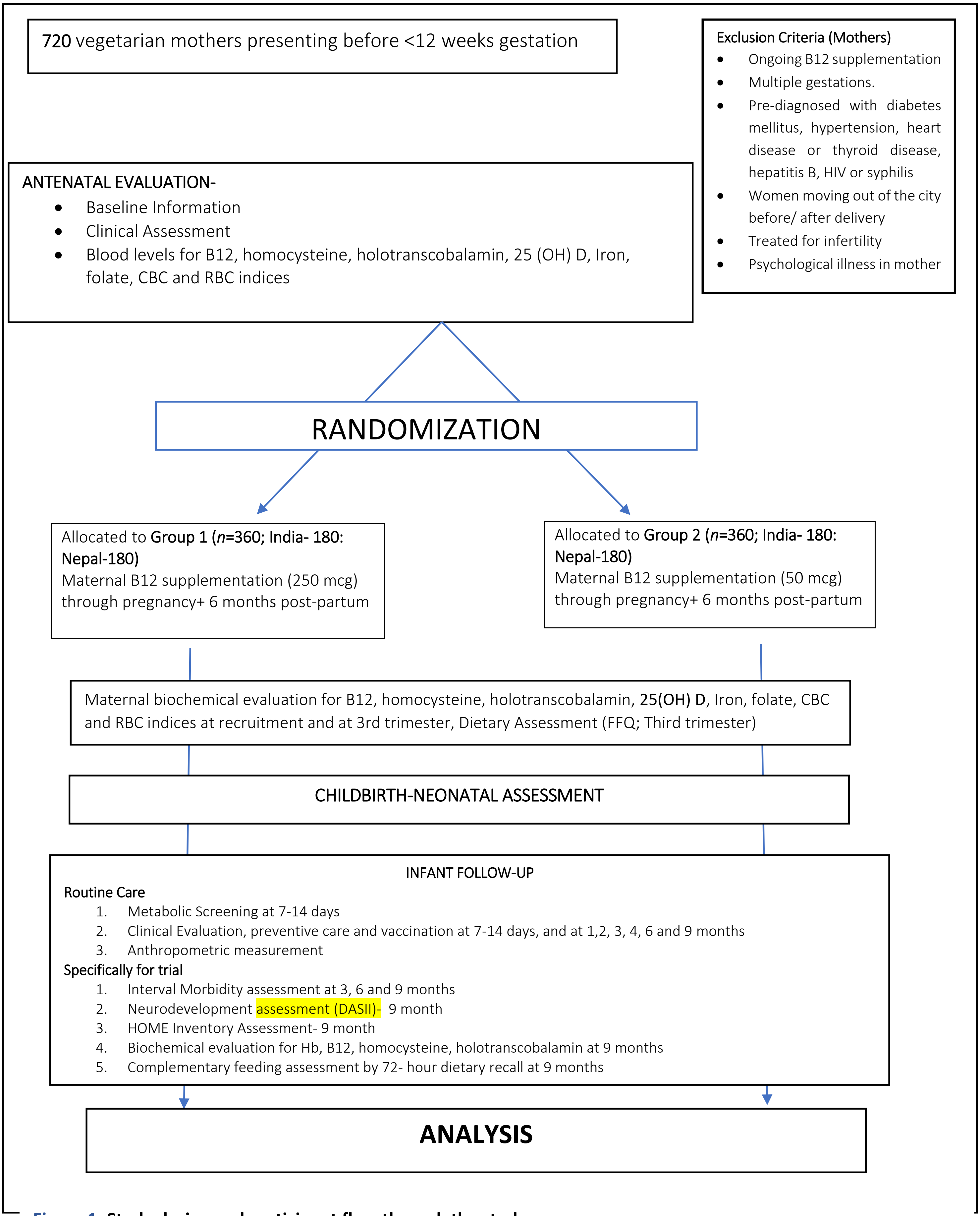

Figure 1. Study design and participant flow through the study.

For peer review only

Supplementary Material: Table 1. Visit schedule

Study Plan	Initial Contact (<12 weeks gestation)		M1	M2-M9	M10	At Birth	I1	I2-I6	I7
**Schedule**	**Screening**	**Recruitment**	**Antenatal Follow-up**	**Antenatal Follow-up (monthly until second trimester, fortnightly in the third trimester**	**Antenatal Follow-up (weekly after 36 weeks until delivery)**		**Infant 7-14 day**	**Infant follow-up: At months- 1 (± 1 wk), 2 (± 2 wks), 3 (± 2 wks), 4 (± 2 wks) & 6 (± 2 wks)**	**Infant follow-up Age 9 months ± 14 days;**
First Screening Visit	X								
Maternal Inclusion/Exclusion criteria	X	X							
Informed consent of the participant		X							
Questionnaire Administration		X							
Randomization		X							
Supplementation		X	X	X	X	X	X	X	
Blood Sampling (1st trimester)		X							
Home Visit (if required)			X	X	X	X	X	X	X
Monitoring of the Compliance			X	X	X	X	X	X	X
Adverse events			X	X	X	X	X	X	X
Blood Sampling (3rd trimester, >26 weeks gestation)				X					
Mother Food Frequency Questionnaire (>26 weeks gestation)				X					
Infant Anthropometry (Weight/length/Head Circumference)						X	X	X	X
Monitoring for neonatal morbidity*						X	X	X	X
Application of Exit Criteria (specified earlier)			X	X	X	X	X	X	X
Metabolic Screening of infant							X		
Infant morbidity							X	X	X
Infant blood sampling									X
Complementary feeding 72 hr. Dietary Recall									X
DASII Scale									X

For peer review only

HOME Inventory									X

For peer review only

Reporting checklist for protocol of a clinical trial.

Based on the SPIRIT guidelines.

Instructions to authors

Complete this checklist by entering the page numbers from your manuscript where readers will find each of the items listed below.

Your article may not currently address all the items on the checklist. Please modify your text to include the missing information. If you are certain that an item does not apply, please write "n/a" and provide a short explanation.

Upload your completed checklist as an extra file when you submit to a journal.

In your methods section, say that you used the SPIRITreporting guidelines, and cite them as:

Chan A-W, Tetzlaff JM, Altman DG, Laupacis A, Gøtzsche PC, Krleža-Jerić K, Hróbjartsson A, Mann H, Dickersin K, Berlin J, Doré C, Parulekar W, Summerskill W, Groves T, Schulz K, Sox H, Rockhold FW, Rennie D, Moher D. SPIRIT 2013 Statement: Defining standard protocol items for clinical trials. Ann Intern Med. 2013;158(3):200-207

		Reporting Item	Page Number
**Administrative information**			
Title	#1	Descriptive title identifying the study design, population, interventions, and, if applicable, trial acronym	1

For peer review only

Trial registration	#2a	Trial identifier and registry name. If not yet registered, name of intended registry	6
Trial registration: data set	#2b	All items from the World Health Organization Trial Registration Data Set	6
Protocol version	#3	Date and version identifier	15
Funding	#4	Sources and types of financial, material, and other support	17
Roles and responsibilities: contributorship	#5a	Names, affiliations, and roles of protocol contributors	1
Roles and responsibilities: sponsor contact information	#5b	Name and contact information for the trial sponsor	17
Roles and responsibilities: sponsor and funder	#5c	Role of study sponsor and funders, if any, in study design; collection, management, analysis, and interpretation of data; writing of the report; and the decision to submit the report for publication, including whether they will have ultimate authority over any of these activities	17
Roles and responsibilities: committees	#5d	Composition, roles, and responsibilities of the coordinating centre, steering committee, endpoint adjudication committee, data management team, and other individuals or groups overseeing the trial, if applicable (see Item 21a for data monitoring committee)	

For peer review only

Introduction

Background and rationale	#6a	Description of research question and justification for undertaking the trial, including summary of relevant studies (published and unpublished) examining benefits and harms for each intervention	4-5
Background and rationale: choice of comparators	#6b	Explanation for choice of comparators	4-5
Objectives	#7	Specific objectives or hypotheses	5
Trial design	#8	Description of trial design including type of trial (eg, parallel group, crossover, factorial, single group), allocation ratio, and framework (eg, superiority, equivalence, non-inferiority, exploratory)	

Methods: Participants, interventions, and outcomes

Study setting	#9	Description of study settings (eg, community clinic, academic hospital) and list of countries where data will be collected. Reference to where list of study sites can be obtained	6
Eligibility criteria	#10	Inclusion and exclusion criteria for participants. If applicable, eligibility criteria for study centres and	8

For peer review only

		individuals who will perform the interventions (eg, surgeons, psychotherapists)	
Interventions: description	#11a	Interventions for each group with sufficient detail to allow replication, including how and when they will be administered	10-12
Interventions: modifications	#11b	Criteria for discontinuing or modifying allocated interventions for a given trial participant (eg, drug dose change in response to harms, participant request, or improving / worsening disease)	
Interventions: adherance	#11c	Strategies to improve adherence to intervention protocols, and any procedures for monitoring adherence (eg, drug tablet return; laboratory tests)	12
Interventions: concomitant care	#11d	Relevant concomitant care and interventions that are permitted or prohibited during the trial	11
Outcomes	#12	Primary, secondary, and other outcomes, including the specific measurement variable (eg, systolic blood pressure), analysis metric (eg, change from baseline, final value, time to event), method of aggregation (eg, median, proportion), and time point for each outcome. Explanation of the clinical relevance of chosen efficacy and harm outcomes is strongly recommended	12-13
Participant timeline	#13	Time schedule of enrolment, interventions (including any run-ins and washouts), assessments, and visits for participants. A schematic diagram is highly recommended	11

For peer review only

		(see Figure)	
Sample size	#14	Estimated number of participants needed to achieve study objectives and how it was determined, including clinical and statistical assumptions supporting any sample size calculations	13
Recruitment	#15	Strategies for achieving adequate participant enrolment to reach target sample size	
**Methods: Assignment of interventions (for controlled trials)**			
Allocation: sequence generation	#16a	Method of generating the allocation sequence (eg, computer-generated random numbers), and list of any factors for stratification. To reduce predictability of a random sequence, details of any planned restriction (eg, blocking) should be provided in a separate document that is unavailable to those who enrol participants or assign interventions	9
Allocation concealment mechanism	#16b	Mechanism of implementing the allocation sequence (eg, central telephone; sequentially numbered, opaque, sealed envelopes), describing any steps to conceal the sequence until interventions are assigned	9
Allocation: implementation	#16c	Who will generate the allocation sequence, who will enrol participants, and who will assign participants to interventions	9

For peer review only

Blinding (masking)	#17a	Who will be blinded after assignment to interventions (eg, trial participants, care providers, outcome assessors, data analysts), and how	9
Blinding (masking): emergency unblinding	#17b	If blinded, circumstances under which unblinding is permissible, and procedure for revealing a participant's allocated intervention during the trial	
**Methods: Data collection, management, and analysis**			
Data collection plan	#18a	Plans for assessment and collection of outcome, baseline, and other trial data, including any related processes to promote data quality (eg, duplicate measurements, training of assessors) and a description of study instruments (eg, questionnaires, laboratory tests) along with their reliability and validity, if known. Reference to where data collection forms can be found, if not in the protocol	10-12
Data collection plan: retention	#18b	Plans to promote participant retention and complete follow-up, including list of any outcome data to be collected for participants who discontinue or deviate from intervention protocols	12
Data management	#19	Plans for data entry, coding, security, and storage, including any related processes to promote data quality (eg, double data entry; range checks for data values).	14

For peer review only

		Reference to where details of data management procedures can be found, if not in the protocol	
Statistics: outcomes	#20a	Statistical methods for analysing primary and secondary outcomes. Reference to where other details of the statistical analysis plan can be found, if not in the protocol	13-14
Statistics: additional analyses	#20b	Methods for any additional analyses (eg, subgroup and adjusted analyses)	13-14
Statistics: analysis population and missing data	#20c	Definition of analysis population relating to protocol non-adherence (eg, as randomised analysis), and any statistical methods to handle missing data (eg, multiple imputation)	
**Methods: Monitoring**			
Data monitoring: formal committee	#21a	Composition of data monitoring committee (DMC); summary of its role and reporting structure; statement of whether it is independent from the sponsor and competing interests; and reference to where further details about its charter can be found, if not in the protocol. Alternatively, an explanation of why a DMC is not needed	
Data monitoring: interim analysis	#21b	Description of any interim analyses and stopping guidelines, including who will have access to these interim results and make the final decision to terminate the trial	
Harms	#22	Plans for collecting, assessing, reporting, and managing solicited and spontaneously reported adverse events and other unintended effects of trial interventions or trial	

For peer review only

		conduct	
Auditing	#23	Frequency and procedures for auditing trial conduct, if any, and whether the process will be independent from investigators and the sponsor	
**Ethics and dissemination**			
Research ethics approval	#24	Plans for seeking research ethics committee / institutional review board (REC / IRB) approval	15
Protocol amendments	#25	Plans for communicating important protocol modifications (eg, changes to eligibility criteria, outcomes, analyses) to relevant parties (eg, investigators, REC / IRBs, trial participants, trial registries, journals, regulators)	15
Consent or assent	#26a	Who will obtain informed consent or assent from potential trial participants or authorised surrogates, and how (see Item 32)	15
Consent or assent: ancillary studies	#26b	Additional consent provisions for collection and use of participant data and biological specimens in ancillary studies, if applicable	
Confidentiality	#27	How personal information about potential and enrolled participants will be collected, shared, and maintained in order to protect confidentiality before, during, and after the trial	15
Declaration of	#28	Financial and other competing interests for principal	17

For peer review only

interests		investigators for the overall trial and each study site	
Data access	#29	Statement of who will have access to the final trial dataset, and disclosure of contractual agreements that limit such access for investigators	17
Ancillary and post-trial care	#30	Provisions, if any, for ancillary and post-trial care, and for compensation to those who suffer harm from trial participation	
Dissemination policy: trial results	#31a	Plans for investigators and sponsor to communicate trial results to participants, healthcare professionals, the public, and other relevant groups (eg, via publication, reporting in results databases, or other data sharing arrangements), including any publication restrictions	14
Dissemination policy: authorship	#31b	Authorship eligibility guidelines and any intended use of professional writers	
Dissemination policy: reproducible research	#31c	Plans, if any, for granting public access to the full protocol, participant-level dataset, and statistical code	14
**Appendices**			
Informed consent materials	#32	Model consent form and other related documentation given to participants and authorised surrogates	
Biological specimens	#33	Plans for collection, laboratory evaluation, and storage of biological specimens for genetic or molecular analysis in the current trial and for future use in ancillary studies, if applicable	

For peer review only

None The SPIRIT checklist is distributed under the terms of the Creative Commons Attribution License CC-BY-ND 3.0. This checklist can be completed online using https://www.goodreports.org/, a tool made by the EQUATOR Network in collaboration with Penelope.ai

For peer review only

BMJ Open

Efficacy of maternal B-12 supplementation in vegetarian women for improving infant neurodevelopment: Protocol for the MATCOBIND multicentre, double-blind, randomised controlled trial

Journal:	*BMJ Open*
Manuscript ID	bmjopen-2019-034987.R3
Article Type:	Protocol
Date Submitted by the Author:	25-Mar-2020
Complete List of Authors:	Nagpal, Jitender; Sitaram Bhartia Institute of Science and Research, Pediatrics<br>Mathur, Manu; Public Health Foundation of India,<br>Rawat , Swapnil ; Sitaram Bhartia Institute of Science and Research, Pediatrics<br>Nagrath, Deepti; Public Health Foundation of India,<br>Lee, Charlotte; University College London, Institute of Child Health<br>Singhal, Atul ; University College London Institute of Child Health<br>Heys, Michelle; Specialist Children's and Young People's Services, East London NHS Foundation Trust<br>Cortina Borja, Mario; UCL, Institute of Child Health<br>Augustin, Katrin ; University College London, Institute of Child Health<br>Gautam , Jageshwor; Paropakar Maternity & Women's Hospital<br>Pant , Rajendra; Paropakar Maternity & Women's Hospital<br>Lakhanpaul, Monica; University College London, Institute of Child Health
**Primary Subject Heading**:	Nutrition and metabolism
Secondary Subject Heading:	Paediatrics, Obstetrics and gynaecology
Keywords:	Maternal medicine < OBSTETRICS, PAEDIATRICS, NUTRITION & DIETETICS, Developmental neurology & neurodisability < PAEDIATRICS, Community child health < PAEDIATRICS

SCHOLARONE™ Manuscripts

I, the Submitting Author has the right to grant and does grant on behalf of all authors of the Work (as defined in the below author licence), an exclusive licence and/or a non-exclusive licence for contributions from authors who are: i) UK Crown employees; ii) where BMJ has agreed a CC-BY licence shall apply, and/or iii) in accordance with the terms applicable for US Federal Government officers or employees acting as part of their official duties; on a worldwide, perpetual, irrevocable, royalty-free basis to BMJ Publishing Group Ltd ("BMJ") its licensees and where the relevant Journal is co-owned by BMJ to the co-owners of the Journal, to publish the Work in this journal and any other BMJ products and to exploit all rights, as set out in our licence.

The Submitting Author accepts and understands that any supply made under these terms is made by BMJ to the Submitting Author unless you are acting as an employee on behalf of your employer or a postgraduate student of an affiliated institution which is paying any applicable article publishing charge ("APC") for Open Access articles. Where the Submitting Author wishes to make the Work available on an Open Access basis (and intends to pay the relevant APC), the terms of reuse of such Open Access shall be governed by a Creative Commons licence – details of these licences and which Creative Commons licence will apply to this Work are set out in our licence referred to above.

Other than as permitted in any relevant BMJ Author's Self Archiving Policies, I confirm this Work has not been accepted for publication elsewhere, is not being considered for publication elsewhere and does not duplicate material already published. I confirm all authors consent to publication of this Work and authorise the granting of this licence.

For peer review only

TITLE PAGE

Title

Efficacy of maternal B-12 supplementation in vegetarian women for improving infant neurodevelopment: Protocol for the MATCOBIND multicentre, double-blind, randomised controlled trial

Running Head: Maternal Vit-B12 and Infant Neurodevelopment Protocol

Authors

Jitender Nagpal[a], Manu Raj Mathur[b], Swapnil Rawat[a], Deepti Nagrath[b], Charlotte Lee[c], Atul Singhal[c], Michelle Heys[d], Mario Cortina-Borja[c], Katrin Augustin[c], Jageshwor Gautam[e], Rajendra Pant[e], Monica Lakhanpaul[c*]

a. *Sitaram Bhartia Institute of Science and Research (SBISR), B16, Qutab Institutional Area, New Delhi, 110016, India.* jitendernagpal@gmail.com; swapnil.rawat@gmail.com
b. *Public Health Foundation of India (PHFI)* Plot Number 47, Sector 44, Institutional Area, Gurugram – 122002, India. manu.mathur@phfi.org ; deepti.nagrath@phfi.org
c. *UCL Great Ormond Street Institute of Child Health, University College London, 30 Guildford Street, London, WC1N 1EH, United Kingdom.* a.singhal@ucl.ac.uk; m.cortina@ucl.ac.uk; k.augustin@ucl.ac.uk; c.lee.12@ucl.ac.uk; m.lakhanpaul@ucl.ac.uk .
d. *Specialist Children's and Young People's Services, East London NHS Foundation Trust, London, UK;* m.heys@ucl.ac.uk.
e. *Paropakar Maternity and Womens' Hospital (PMWH), Thapathali, Kathmandu, Nepal.* drjgautam48@gmail.com; rajendrapant8@gmail.com .

*Corresponding Author

Professor Monica Lakhanpaul; UCL Great Ormond Street Institute of Child Health, University College London, 30 Guildford Street, London, WC1N 1EH, United Kingdom; m.lakhanpaul@ucl.ac.uk .

Word Count

Abstract: 250

Main text: 4846

For peer review only - http://bmjopen.bmj.com/site/about/guidelines.xhtml

Tables: 1

Figures: 1

Key Words- Maternal Health, Vitamin B-12, Infant Neurodevelopment

ABSTRACT

Introduction: Vitamin B-12 deficiency is widely prevalent across many low and middle-income countries, especially where the diet is low in animal sources . While many observational studies show associations between B-12 deficiency in pregnancy and infant cognitive function (including memory, language, and motor skills), evidence from clinical trials is sparse and inconclusive.

Methods and Analysis: This double blind, multi-centre randomised controlled trial will enrol 720 vegetarian pregnant women in their first trimester from ante-natal clinics at two hospitals (one in India and one in Nepal). Eligible mothers who give written consent will be randomised to receive either 250 mcg methylcobalamin or 50 mcg (quasi control), from enrolment to 6 months post-partum, given as an oral daily capsule. All mothers and their infants will continue to receive standard clinical care. The primary trial outcome is the offspring's neurodevelopment status at 9 months of age, assessed using the Development Assessment Scale of Indian Infants. Secondary outcomes include the infant's biochemical B-12 status at age 9 months and maternal biochemical B-12 status in the first and third trimesters. Maternal biochemical B-12 status will also be assessed in the first trimester. Modification of association by apriori identified factors will also be explored.

Ethical Considerations and Dissemination: The Study Protocol has been approved by ethical committees at each study site (India and Nepal) and at UCL, UK. The study results will be disseminated to healthcare professionals and academics globally via conferences, presentations and publications. Researchers at each study site will share results with participants during their follow-up visits.

For peer review only - http://bmjopen.bmj.com/site/about/guidelines.xhtml

ARTICLE SUMMARY

Study strengths

- Large-scale, multi-centre, randomised controlled, double-blind trial across a low-middle income country (LMIC) and low-income country (LIC) to determine the efficacy of maternal vitamin B-12 supplementation during pregnancy on neurodevelopmental outcomes six-month post-partum.
- Study design benefits from the preceding qualitative patient and public involvement (PPI) and nested qualitative sub-study, ensuring participant feedback and process evaluation to optimise trial conduct.
- The study is being conducted across two diverse socio-demographic populations to increase generalisability of study findings.

Study limitations

- Normal/Routine care differs at the two sites, which could lead to differences in the conduct and monitoring of the trial.
- Health system regulatory structures differ in the two countries.

For peer review only - http://bmjopen.bmj.com/site/about/guidelines.xhtml

1. INTRODUCTION

Malnutrition during pregnancy is a global health problem. Micronutrient deficiencies are associated with adverse developmental outcomes in children, including poorer neurocognitive functioning which can potentially lead to poorer educational achievement and economic prospects [1, 2].

Vitamin B-12 deficiency is prevalent across many low and middle-income countries (LMICs) [3-6]. Partaking in a vegetarian diet, or one low in animal food sources, is a key risk factor in developing B-12 deficiency [7, 8]. Sufficient B-12 intake during pregnancy is vital for foetal brain development [9]. Deficiency of B-12 in pregnancy is linked to infant birth defects, intrauterine growth restriction, preterm delivery and neural tube defects [10-14]. Many observational studies have shown associations between B-12 deficiency during pregnancy or early infancy with subsequent poorer infant neurodevelopment [15] and cognitive function [16, 17] including memory [18], language, and motor skills [19].

There is limited evidence from randomised controlled trials (RCTs) regarding the effect of vitamin B-12 supplementation and infant cognitive outcomes [20,21,22]. An infant supplementation trial observed neurodevelopmental benefits of infant B-12 supplementation with folate, and documented 0.45 (95% CI 0.19, 0.73) and 0.28 (95% CI 0.02, 0.54) higher SD-units in the domains of gross motor and problem-solving functioning, respectively [22]. However, in this study there was no significant benefit of giving vitamin B-12 or folic acid alone on early child development (with the exception of vitamin B-12 in relation to gross motor functioning [22]). In another trial, maternal vitamin B-12 supplementation given as 50mcg a day from <14 weeks' gestation through to 6 weeks postpartum did not improve infant neurodevelopmental outcomes at 9 months [21]. In this trial hyperhomocysteinemia (a marker of B-12 insufficiency) during pregnancy was negatively associated with infant cognitive outcomes. The study documented a deterioration in biochemical B-12 status in the placebo group, while the rise in B-12 status in the supplemented group (50 mcg) was inconsistent (no significant difference between first and third trimester levels). Additionally, a high rate of participant attrition (51.4%), poor monitoring of supplement compliance, a lack of biochemical improvement in B-12 status in the supplemented group of mothers, and lack of infant biochemical evaluation for B-12 status in later infancy, further limit the inferences that can be drawn from this trial. Other studies that assessed a 250mcg dose of B-12 per day throughout pregnancy until 3 months' post-partum in Bangladesh [23], and >2000mcg from 17 to 34 weeks' gestation in India [24] did not evaluate neurodevelopment outcomes in infancy. Hence, there is insufficient evidence concerning timing, dose, duration and efficacy of B-12 supplementation with reference to infant neurodevelopment.

For peer review only - http://bmjopen.bmj.com/site/about/guidelines.xhtml

To address this evidence gap, we are conducting a double-blind multi-centre RCT in one Low- and Middle-Income Country (LMIC; India) and one Low- Income Country (LIC; Nepal), which will evaluate the efficacy of 250mcg/day vitamin B-12 supplementation in vegetarian mothers from the first trimester to 6 months' post-partum on infant neurodevelopmental outcomes at age 9 months. Although, several published protocols report ongoing work on B-12 supplementation in India and Nepal [25,26], the proposed work differs significantly in several ways.

First, we specifically chose to supplement vegetarian women, as they are a high-risk population due to their low B-12 intake, and as such have a high prevalence of B-12 deficiency. Second, an interventional dose of 250 mcg was chosen because it has been shown in an earlier trial to improve B-12 levels in pregnancy [23] (although infant neurodevelopment was not assessed in this trial). Third, we chose 50 mcg B-12 /day as a quasi- control group as this dose has been shown to prevent deterioration in B-12 status during pregnancy in India [21]. Furthermore, given the progressive decline in B-12 levels throughout pregnancy (documented in the placebo group of earlier trials [21,22]), and the potentially serious neonatal consequences of severe B-12 deficiency highlighted above, a pure placebo control was thought to be unethical.

2. AIMS AND OBJECTIVES

Primary objective

To determine the effect of 250mcg versus 50mcg daily oral vitamin B-12 supplementation of pregnant women from the first trimester to 6 months post-partum on infant neurodevelopment at age 9 months.

Secondary objectives

To determine the effect of 250mcg versus 50mcg of oral vitamin B-12 supplementation in pregnancy and 6 months post-partum on biochemical parameters of B-12 status (Vitamin B-12 levels, Homocysteine and Holo-transcobalamin) in mothers at the end of the third trimester and in infants at age 9 months.

Tertiary objectives

1. To evaluate the influence of the following effect modifiers:
   a. Socio-economic indicators (income, education, profession) on the relationship between maternal B12 status, supplementation and infant neurodevelopment;

For peer review only - http://bmjopen.bmj.com/site/about/guidelines.xhtml

For peer review only

b. Type of milk feeding on the infant vitamin B-12 status and infant neurodevelopment at 9 months;

c. Maternal dietary B-12 intake with maternal and infant B-12 status, supplementation and infant neurodevelopment;

d. Infant complementary feeding with infant vitamin B-12 status and infant neurodevelopment at 9 months;

e. Maternal and infant B-12 status and neurodevelopment at 9 months after birth.

2. To determine the interaction of maternal iron and vitamin D status with the relationship between infant vitamin B-12 status and infant neurodevelopment at 9 months.

3. METHODOLOGY

Trial Registration:

The study is registered as a primary clinical trial at the WHO International Clinical Trials Registry Platform through the Clinical Trial Registry of India (CTRI/2018/07/015048) and at ClinicalTrials.gov (ID NCT04083560).

Setting

This study includes two sites: Delhi, India and Kathmandu, Nepal. Both countries have documented a high prevalence of B-12 deficiency [5,6].

For peer review only

For peer review only - http://bmjopen.bmj.com/site/about/guidelines.xhtml

Participants

Mothers will be recruited from the 1) Sitaram Bhartia Institute of Science and Research (SBISR), New Delhi, India, a privately- funded, tertiary hospital catering for a high and middle-income population, with approximately 700 deliveries annually; and 2) Paropakar Maternity and Women's Hospital (PMWH), Kathmandu, Nepal, a government hospital catering for a poorer socio-economic area with approximately 22,000 deliveries annually. Our earlier work (2011- 2014) documented that the majority of mothers delivering at SBISR were well-educated (96.8% college education), employed (63.2%) living in high income (annual income 16,000 USD) nuclear families (57.9%) [27]. A directly comparable dataset was not available from PMWH.

Feasibility Data and Preliminary Work

Feasibility of data and preliminary qualitative work was conducted for the trial in 2017-18. Feasibility of recruitment within the given timelines was studied on the basis of previous data available from the study sites. The lead author (JN) examined neurodevelopmental outcomes of 413 babies born at SBISR in a cohort study conducted between 2011 and 2014. The project had a similar design to that proposed for this trial, using antenatal recruitment and post-delivery infant neurodevelopmental follow-up [27]. Approximately 2/3 (67%) of subjects were retained to follow-up in the cohort study. The loss to follow-up (33%) and speed of recruitment have been considered in the sample size calculations and the recruitment timeline for this trial. No similar recruitment and retention data were available from PMWH.

Patient and Public Involvement:

To represent the participants' perspective, a Patient and Public Involvement (PPI) panel was established consisting of four parent pairs (2 pairs per site) who had delivered a baby within the previous 2 years. One parent pair has continued to be involved with the project development and the Trial Oversight Committee (TOC). We conducted an exploratory study with pregnant mothers at both study sites to better understand the factors likely to influence the uptake and implementation of the proposed intervention, and proposed barriers to implementation (data unpublished). This qualitative study established that most respondents were unaware of B-12 dietary sources and the importance of B-12. All the participants involved in this exploratory study understood the burden of the intervention and were eager to know about symptoms, treatment and prognosis of B-12 deficiency. Respondents were also willing to accept the intervention and the need for drawing extra blood from the baby as a part of routine sampling for B-12 levels. Perceived barriers to the study included

For peer review only - http://bmjopen.bmj.com/site/about/guidelines.xhtml

concerns around potential side effects of the B-12 supplement, relocation mid-trial, and financial difficulties. All of these factors were considered in the study design, development of patient information, consultation, and follow-up procedures as follows:

- All participants at the two study sites will be informed of the role, sources and importance of vitamin B-12 in human health using educational banners and posters and through community workshops;
- The initial information about the trial will be given by the doctor providing antenatal care to the mother. Subsequent detailed consent and recruitment will be conducted by study research staff;
- In order to improve compliance and study retention, reminder text messages will be sent weekly to participating mothers;
- To avoid the risk of financial loss to participants, various biochemical tests at the Indian trial site will be sponsored, and transportation costs at the Nepal site will be covered;
- Bradleys' Home Observation for Measurement of the Environment (HOME) inventory was added to the assessment given the divergent environmental profile of the two study sites [28], in order to assess the infant's environment and possible environmental confounders on neurodevelopment;

The study results will be disseminated globally to academics and health professionals via conferences, presentations and publications. Researchers at each study site will share results with participants during their follow-up visits.

Also, in preparation for this trial we adapted a pre-validated Food Frequency Questionnaire (FFQ) for Vitamin B-12 intake [29] to the Indian and Nepalese settings. In brief, the original Vitamin B-12 FFQ by Gael et al. (including 30 questions on consumption of food and beverages containing VitaminB-12) was reviewed independently by two nutritionists. A market survey was done to obtain information on egg and milk products consumption, as well as the availability of B-12 fortified items at local markets in Delhi and Kathmandu. Homemade recipes which included egg and/or dairy products as a main ingredient were incorporated into the FFQ. Measurements used in the FFQ were standardised based on the typical/locally available household utensils to ensure accurate responses and to maintain consistency across the sites.

All research staff from India and Nepal will be jointly trained in study design, research methodology, good clinical practice, the study protocol, and study tools. Training lectures will be video-recorded and made available to the teams at both study sites as a reference material for continuous training.

For peer review only - http://bmjopen.bmj.com/site/about/guidelines.xhtml

For peer review only

Eligibility

Potentially eligible participants will be screened at first presentation to the antenatal clinic at ≤12 weeks of gestation in accordance with the following criteria:

Inclusion Criteria:

- Consuming a vegetarian diet -including veganism and/or people who do eat egg and/or do consume milk and/or a portion meat/chicken/fish ≤ once a month;
- Living within an *a-priori* defined geographical area;
  - Delhi – national capital region;
  - Nepal – 10-km radius of PMWH;
- Able to understand at least one of the following languages-English, Hindi or Nepalese.

Exclusion Criteria:

- Wish for medical termination of pregnancy;
- Aged <18 or >35 years;
- Already on medicinal vitamin B-12 supplementation (including B-complex or multivitamins);
- Multiple gestation;
- Diagnosis of a chronic medical condition (including diabetes mellitus, hypertension, heart disease, neurological disease, or thyroid disease);
- Testing positive for hepatitis B, human immunodeficiency virus (HIV), or syphilis;
- Anticipating moving out of the *a-priori* defined geographical area before or after delivery;
- Undergoing treatment for infertility;
- Having a known pre-diagnosed mental health disorder, including depression, drug or alcohol abuse;
- Currently participating or having participated in another study within four weeks prior to the trial commencing;
- Having a known allergy to vitamin B-12 or another supplement constituent.

Consent

Eligible participants meeting the selection criteria will be given time to think over continuation of pregnancy, site of delivery, participation in the trial and any other considerations. They will be contacted within 48 hours by the research team regarding their decision to participate, and will be

For peer review only - http://bmjopen.bmj.com/site/about/guidelines.xhtml

invited to provide written or video-recorded consent. Participants will be able to opt into the trial any time before 12 weeks of gestation.

Randomisation

As presented in Figure 1, all eligible and consenting participants will be randomised with an allocation ratio of 1:1 to either the intervention (250mcg), or control (50mcg) group. The randomisation sequence will be generated at a single, third-party location (non-study site) using a computer-generated sequence stratified for location. The sequence will be allocated to serial numbers by a neutral person in each country using sequentially numbered, sealed, opaque envelopes. Codes within envelopes will identify boxes labelled with 5-digit codes. Each box will contain enough dosages to cater to the study period of one participant. The strips containing intervention and control doses of B-12 will be identical in taste, smell and appearance. All participants, recruiters, developmental therapists, laboratory staff, and the data analyst will be blinded to the B-12 allocation until results of final analysis are made available.

Figure 1. Study design and participant flow through the study.

Procedure

Upon enrolment, demographic and clinical details, including age, height, weight, ethnicity, education and socio-economic status, will be collected. The participants' preferred channel of subsequent communication (phone, text message, email) will be noted. Participants will be allocated a study identification number and accompanied by the researcher for blood sampling. These samples will be taken at the same time as other samples being drawn in the first trimester to minimise unnecessary venepunctures. Twelveml of blood sample will be drawn into two plain vials (5ml each) and 2ml will be drawn into an EDTA vial. The EDTA sample will be processed immediately for complete blood counts by coulter counter. The plain (serum separator tubes) vial samples will be centrifuged at 3500 rpm (Rotor radius 15 cm) for 10 minutes at the study site. The separated serum will be transported on ice and consistently stored at -70° C, and will be monitored at both the study sites. Participants will be given enough B-12 supplement doses to last five days beyond the expected date of their next visit, after being fully informed about the supplement and its administration. Participants will be contacted

For peer review only - http://bmjopen.bmj.com/site/about/guidelines.xhtml

48 hours after receiving the B-12 doses to confirm initialisation of supplementation and re-emphasise compliance.

Subsequent maternal visits will take place monthly until 36 weeks, and then weekly thereafter. At these visits, all participants will visit their obstetrician as part of routine care. Participants will also meet with the research staff at a pre-specified location at the study site. To maximise compliance, weekly adherence and pre-appointment reminders will be sent via the pre-specified preferred mode of communication. Acquired morbidity (gestational diabetes, hypertension, and hypothyroidism) and medication history will be noted in case record forms (CRFs). Empty supplement packets will be collected at each appointment as an indicator of adherence. In the third trimester, mothers' dietary B-12 intake will be assessed using a pre-standardised and adapted FFQ, and blood sampling will be repeated using the same procedures as in the first trimester.

Neonatal assessment: During childbirth, a medical officer and/or paediatrician will monitor the birth and post-delivery health of neonates for any morbidity that might potentially influence neurodevelopment. Gestational age, growth retardation, congenital abnormalities and APGAR (appearance, pulse, grimace, activity, respiration) score will be routinely documented for neonates. Cord pH will be measured for all new-borns with a delayed cry and who require any intervention beyond the basic steps of resuscitation (ambu bagging, intubation, or pharmacological treatment). Any seizures, neurological problems, hypoglycaemia, hypothermia, or hyperbilirubinemia will be assessed, diagnosed, evaluated, and managed as per institutional guidelines. As per standard practice, all new-borns will be screened for hearing deficits, vision, and critical congenital heart disease according to institutional protocols. Preterm neonates transferred to the neonatal unit will be monitored as per their customized follow-up and early intervention plans, implemented as recommended by the paediatrics and/or primary care provider. Neurodevelopment and biochemical assessment of preterm neonates will be carried out as per their corrected gestational ages.

Infant follow-up will be conducted at 7-14 days, 4, 8, 12 and 16 weeks and at 6 and 9 months of age. At these visits, the infants will undergo routine care as well as study specific evaluations (see Visit Schedule: Supplementary Table 1).

Routine care: Each mother-infant dyad will see a paediatrician for age-appropriate routine care, which will involve clinical evaluation, preventative care and vaccination as per standard procedures in each site. Anthropometric measurements and signs of micronutrient deficiency (anaemia and rickets) will

For peer review only

be recorded during each visit as part of routine care. Metabolic disorders (congenital hypothyroidism, congenital adrenal hyperplasia, cystic fibrosis, biotinidase deficiency, phenylketonuria and galactosemia) will be screened for and maternal support for breastfeeding by a paediatrician or lactation counsellor will be provided at 7-14 days.

Study specific: Supplements will be provided to mothers at each visit, with instructions to take until 6 months after childbirth. Any morbidities and deficiencies will be recorded and treated as per institutional guidelines. Maternal and infant tolerance for the supplementation, including any gastrointestinal symptoms, will be recorded at each visit, as will maternal compliance. Provision of supplements to the mother in both groups will be stopped at 6 months post-partum.

The hospital evaluation at 9 months (±14 days) will include neurodevelopmental assessment by a developmental therapist using the Developmental Assessment Scales for Indian Infants (DASII) - an adapted and validated version of the Bayley's Scale for Infant Development Third Edition (BSID II) [30] - and blood sampling for estimation of Haemoglobin, Vitamin B-12, Homocysteine and Holo-transcobalamin concentrations. The DASII motor scale assesses gross and fine motor skills, whilst cognitive, personal and social skills development are assessed through the DASII mental scale. All neurodevelopmental assessments will be conducted by a trained developmental therapist at the institute in a soundproof room. In premature babies, corrected gestational age will be used to calculate developmental quotients. Additional assessments will include a complementary feeding assessment by a nutritionist using a 72-hour dietary recall, and a home environment assessment using the adapted Bradley HOME inventory [28] by a trained field worker.

Compliance: Research staff will dispense blister packaging of 10 B-12 capsules and study specific bags for participants to store, carry, and keep supplements in one place. In case of loss of supplement or inability to come for follow-up, participants will be able to contact the researcher at any time to request more supplements. The supplement packaging will be numbered with a serial number. To maximise compliance, weekly reminders will be sent through the preferred mode of communication as specified by the trial participants. Each participant will be provided with a bag to save empty packaging strips and instructed to carry the bag with her at the next visit to monitor compliance.

Biochemical analysis

All saved samples will be processed as one batch after the completion of the study. The maternal samples will be used to determine the blood levels for Vitamin B-12, Homocysteine, Holo-

For peer review only - http://bmjopen.bmj.com/site/about/guidelines.xhtml

transcobalamin, 25(OH)D, folate, Iron Studies (Serum Iron, Ferritin and Total Iron Binding Capacity), Complete Blood Count (CBC) and Red Blood Cell (RBC) indices in the first and third trimesters. Infant blood will be used for Haemoglobin, Vitamin B-12, Homocysteine and Holo-transcobalamin measurements. Vitamin B-12 and 25(OH)D will be measured by Enhanced CLIA (Johnson & Johnson, USA). Iron levels will be estimated by Pyridylazodye (Johnson & Johnson, USA). Folate will be analysed by Enhanced Chemiluminescence Immunoassay (CLIA) (Beckman Coulter, USA), and CBC by Beckman Coulter, USA. Red Blood Cell(RBC) indices will be calculated by Impedance (Beckman Coulter, USA). Homocysteine and Holo-transcobalamin will be analysed by Chemiluminescent Microparticle Immunoassay (CMIA).

Outcomes:

The following study outcomes will be investigated:

Primary Outcome:

- DASII scores for infants at 9 months (±2 weeks) post-partum.

Secondary Outcome (s):

- Change in B-12 status (vitamin B-12 level, Homocysteine, and Holo-transcobalamin) of mother between first (≤12 weeks' gestation) and third trimester (≥ 27 weeks' gestation);
- B-12 status (vitamin B-12 level, Homocysteine, and Holo-transcobalamin) of infants at 9 months (±2 weeks) post-partum.

Exploratory Outcome(s):

- Haemoglobin (Hb) of infants at 9 months (±14 days) post-partum;
- Infant anthropometry including weight, height/ length and head circumference at 9 months after birth in all subjects.

Sample size considerations

A planned sample of 720 pregnant women, 360 per study site, will be randomly assigned to experimental (*n*=360; *n*=180 in per study site) or control (*n*=360; *n*=180 in per study site) groups. This is based on the number of infants needed to complete the neurodevelopmental screen in each of the two randomised groups to detect a difference in Development Quotient (DQ) at nine months (primary outcome) of 2 points (presumed mean DQ in this population of infants at 9 months of 96.9, SD 7.07

For peer review only - http://bmjopen.bmj.com/site/about/guidelines.xhtml

on basis of earlier work)([27]. The difference of 2 DQ points was chosen because it is the smallest effect size thought to be clinically significant, and hence a null trial result is likely to exclude a clinically relevant effect of maternal B12 supplementation on infant cognitive function. The sample size has been inflated by 35% to account for attrition, and assumes 90% statistical power and a 5% two-sided significance level.

Statistical analysis

The primary and secondary outcomes will be analysed using an intention-to-treat analysis by a blinded statistician. Comparability of participants assigned to study groups at enrolment will be assessed using tests of association for categorical variables and by fitting generalised linear models for continuous variables. These models will include interaction terms to explore conditional differences between the trial's arms. If necessary, transformations will be applied to the response variables to fulfil the model's assumptions. Covariates will include trial site, infant feeding, gender, feeding by gender, weight *z*-score at birth, weight gain between birth and 3 months, and maternal body mass index (BMI). *Z*-scores for height and weight will be calculated according to the World Health Organisation [31] growth standards using the R language for statistical computing [32].

Subgroup analysis will be conducted to examine the differential impact of B-12 supplementation in high versus low risk mothers and infants. Mothers identified as having pregnancy-related or other morbidity will be classified as high-risk (e.g. including gestational diabetes, pregnancy-induced hypertension/pre-eclampsia/eclampsia and absent or reversed end diastolic flow); and low-risk. Babies will be defined as high risk if there is any combination of the following:

- Small for gestational age [SGA], defined as <10th centile for birth weight;
- Very preterm (28-32 weeks gestation); Extremely preterm babies (<28 weeks) will be excluded from the analysis;
- Experience of a higher risk neonatal course defined as experience of APGAR score <7 at 1 minute, hypoglycaemia, hyperbilirubinemia or prolonged neonatal intensive care stay >7 days);
- Infants with documented neurological disease/anomaly/illness will be sub-grouped separately (high risk) and compared to those with no disease/anomaly illness (low risk).

For peer review only - http://bmjopen.bmj.com/site/about/guidelines.xhtml

Data management and dissemination

Data will be collected under the regulation of the data protection and management guidelines provided by the Governments of India and Nepal, and as per the UK Data Protection Act 2018. Data will be securely stored in an E-database with password protection. The E-database will be designed using Access. Live data entry will be conducted at the study sites and tested for accuracy using duplicate data entry at an offsite location at periodic intervals.

The study results will be disseminated globally with academic and health professionals via conferences, presentations and publications. Researchers at each study site will share emerging results with participants during their follow-up visits. Regular site meetings with press offices, short films, radio spots, periodic blog posts, and Twitter and Facebook messages are also planned to strengthen the trial's online presence and reach young and middle-aged internet users for whom the information may be of interest.

Qualitative evaluation

A qualitative analysis of the information learned during preliminary PPI work aimed to explore knowledge, attitudes and perceptions of pregnant women towards B-12 supplementation and child development, and the potential barriers to implementation. Better understanding these domains across the two study sites informed the perceived need for the intervention, helped define patient-centric outcomes and allowed the evaluation of any differences between the two arms.

We used convenience sampling to recruit and interview 25 participants across both trial sites (SBISR 12; PMWH 13). We developed and piloted a semi-structured topic guide in collaboration with our PPI representatives serving on the Trial Steering Committee (TSC). The topic guide was designed in English and translated into either Hindi or Nepali. The topic guide was then back-translated into English to ensure consistency and accuracy of content. It was created with the intention to help direct the interview whilst remaining sensitive to unsolicited themes. We ensured that an interviewer with prior training and experience in qualitative methods facilitated the semi-structured interviews.

Data was collected in quiet rooms in each hospital, away from other patients and staff. Interviews were facilitated in English, Hindi or Nepalese, depending on the preference of the participant. All interviews were audio recorded, transcribed, translated into English (where necessary) and then crosschecked against the original recording. The translated transcripts were then coded using the Atlas Ti 7.2 software for qualitative analysis [33]. A research team member coded and categorised the data, and prepared an initial narrative. A report detailing the findings is in the process of being finalised, with input from the interviewers and wider research team.

For peer review only - http://bmjopen.bmj.com/site/about/guidelines.xhtml

Ethical considerations:

The Study Protocol (10th December 2018. Version: 8.0), Informed Consent Form, Participant Information Sheet, Tool and other supportive materials have been approved by the Institutional Ethics Committee (IEC) at SBISR (FL/SBISR/IEC/2018-01); PHFI (TRC-IEC-380/18); PMWH (59-11-433); Nepal Health Research Council Ethical Review Board (NHRC) (Reg No. 761/2018) and the UCL Research Ethics Committee (10901/001). The Investigators at each study site have submitted to, and where necessary, obtained approval from the above parties for all substantial amendments to the original approved documents.

The trial will be conducted in accordance with the principles and rules of the Declaration of Helsinki. Informed and written consent will be obtained from all participants by the research teams at each study site. The trial will comply with the Data Protection Act (UK), 2018, ensuring the participants' anonymity. The participants will be identified only by a participant identification number on all trial documents and electronic databases. Every effort will be made to arrange interviews and examinations at a time convenient to the participants, without causing much disruption in their daily routine. Study data will be stored securely and will only be accessible to trial staff and authorised personnel. The Principal Investigator at each study site will submit an annual progress report, end of trial notification and a final report to the Institutional Ethics Committee, host organisations and the funders.

We used the SPIRIT checklist for writing our protocol [34].

DISCUSSION

A positive result from this study will have considerable impact on current and future policy initiatives, by underpinning current micronutrient supplementation policy in India and Nepal. The project will provide key insights to strengthen existing Indian governmental nutrition programmes such as "National Iron Plus Initiative", "Janini Suraksha Yojna" and "Janini Shishu Suraksha Kariyakram". Because of the high worldwide prevalence of maternal B-12 deficiency (74%), the study findings will also be highly relevant to maternal and neonatal health in nutritionally vulnerable and at-risk populations globally.

Using a 50 mcg quasi-control group limits the inferences that can be drawn from a negative result (neurodevelopment comparable between the two groups). The study outcome could produce several results, which could be interpreted as follows:

1. *Findings:* Improvement in B-12 parameters from first trimester to third trimester is statistically significant in both groups, and the change is statistically comparable between two groups for

For peer review only - http://bmjopen.bmj.com/site/about/guidelines.xhtml

For peer review only

mother and baby. Substantial proportion of mother/infants in both groups improve from B-12 deficient to replete state.

Interpretation: 250 mcg dose is as effective as 50 mcg in improving biochemical parameters and both doses result in comparable neurodevelopment. 250 mcg dosing offers no additional advantage over 50 mcg.

2. *Findings:* Improvement in B-12 parameters from first trimester to third trimester is significant in either 250 mcg alone or both groups, but 250 mcg causes higher rise (statistically significant) in B-12 biochemical parameters than 50 mcg for mother and baby.

   Interpretation: 250 mcg is more efficacious than 50 mcg in improving biochemical B-12 status. Since neurodevelopment is equivalent between two groups a higher dose of supplementation does not offer any neurodevelopmental advantage despite better biochemical B-12 status.

3. *Findings:* A lack of biochemical improvement in both groups, although unexpected, would be interpreted to mean poor efficacy of 50 mcg or 250 mcg oral B-12 in improving B-12 status of mother-infant pairs. Both groups continue to have a high proportion of mothers deficient in B-12 before and after supplementation.

   Interpretation: Factors affecting B-12 status in the current trial (including compliance and socio-demographics) would have to carefully analysed. Further work would be required to study the determinants of oral B-12 efficacy in detail.

Many studies have evaluated the impact of varying Vitamin B-12 intake (physiological (2 mcg/d) to pharmacological dosage (up to 2000 mcg/d); supplemented/observed) in diverse population sets [20-24, 35]. Our study specifically seeks to address the question of the optimum dosage for vegetarian pregnant women. As one of the few large-scale trials of B-12 supplementation in pregnancy, the results of this study would be generalisable to vegetarian expecting mothers. We aim to contribute to the evidence base on improving neurodevelopmental outcomes in vulnerable infants. While a positive outcome for the trial would help to develop an intervention program to reduce developmental delay using Vitamin B-12 as a supplementation in pregnant women, a negative or null result would also add to existing knowledge and lay the groundwork for future work on the subject - especially around utility, dose, duration and time of initiation of Vitamin B-12 supplementation.

DECLARATIONS

Acknowledgements: CL and ML were partly supported by the National Institute for Health Research (NIHR) Collaborations for Leadership in Applied Health Research and Care (CLAHRC) North

For peer review only - http://bmjopen.bmj.com/site/about/guidelines.xhtml

Thames at the time of this work. We would also like to thank our Patient and Public Involvement (PPI) panel. MH is funded by East London NHS Foundation Trust.

Author Statements: JN, MM, and ML conceived the study and AS, MH, MCB, JG, RP, ML, CL contributed to the study design. SR and DN conducted the qualitative study, adaptation of FFQ, standardisation of questionnaires and organised the training workshop. SR, DN, KA and CL drafted the manuscript. JN, MM and ML finalised the manuscript, and all authors assisted in developing the protocol and have read, reviewed, edited and approved the final manuscript.

Funding: The study is funded by the Newton Fund: The Department of Biotechnology (DBT) India, in collaboration with Department of International Development (DFID), the Economic and Social Research Council (ESRC) and the Medical Research Council (MRC) consortium (Grant Number MR/R020396/1). This research was supported by the National Institute for Health Research (NIHR) Biomedical Research Centre based at UCL Great Ormond Street Institute of Child Health/Great Ormond Street Hospital NHS Foundation Trust.

Competing interests: All authors declare no competing interests.

Provenance and peer review: Not commissioned; externally peer reviewed

Data sharing statement: For access to the data set, a formal request should be sent to the MATCOBIND study group at manu.mathur@phfi.org. The request will only be considered when the results of the study have been published.

For peer review only

For peer review only - http://bmjopen.bmj.com/site/about/guidelines.xhtml

REFERENCES

1. Vohr BR, Poggi Davis E, Wanke CA, Krebs NF. Neurodevelopment: The Impact of Nutrition and Inflammation During Preconception and Pregnancy in Low-Resource Settings. Paediatrics. 2017;139(Suppl 1): S38–S49.
2. Victora CG, Adair L, Fall C, et al. Maternal and child undernutrition: consequences for adult health and human capital. Lancet. 2008;371(9609):340–57.
3. Allen, LH. Folate and vitamin B12 status in the Americas. Nutr Rev. 2004 Jun; 62(6): S29-33.
4. Allen, LH. Causes of vitamin B12 and folate deficiency. Food Nutr Bull. 2008 Jun;29(2):20-34.
5. Ulak M, Chandyo RK, Adhikari RK, et al. Cobalamin and folate status in 6 to 35 months old children presenting with acute diarrhea in Bhaktapur, Nepal. PLoS One 2014;9: e90079.
6. Taneja S, Bhandari N, Strand TA, Sommerfelt H, Refsum H, Ueland PM, Scheede J, Bahl R, Bhan MK. Cobalamin and folate status in infants and young children in a low-to-middle income community in India. Am J Clin Nutr. 2007; 86:1302–1309.
7. Sklar R. Nutritional vitamin B12 deficiency in a breast-fed infant of a vegan-diet mother. Clin Pediatr (Phila). 1986;25(4):219–21.
8. Kuhne T, Bubl R, Baumgartner R. Maternal vegan diet causing a serious infantile neurological disorder due to vitamin B12 deficiency. Eur J Pediatr. 1991;150(3):205-208.
9. Pepper MR, Black MM. B12 in foetal development. *Semin Cell Dev Biol*. 2011;22(6):619–623. doi: 10.1016/j.semcdb.2011.05.005
10. Lee C, Maza, Stern B, Lakhanpaul M, Parikh P. Associations between the household environment and stunted child growth in rural India: a cross-sectional analysis. UCL Open: Environment. 2019: doi: 10.14324/111.444/000015.v1.
11. Ganguly P, Alam SF. Role of homocysteine in the development of cardiovascular disease. *Nutr J*. 2015; 14:6. 2015 Jan 10. doi:10.1186/1475-2891-14-6.
12. Black MM. Effects of Vitamin B12 and Folate Deficiency on Brain Development in Children. Food Nutr Bull. 2008;29(2): S126–31.
13. Molloy AM, Kirke PN, Troendle JF, Burke H, Sutton M, Brody LC, Scott JM, Mills JL. Maternal vitamin B12 status and risk of neural tube defects in a population with high neural tube defect prevalence and no folic acid fortification. Pediatrics. 2009; 123:917–923.
14. Refsum H. Folate, vitamin B12 and homocysteine in relation to birth defects and pregnancy outcome. Br J Nutr. 2001;85: S109–S113.
15. Del Río Garcia C, Torres-Sánchez L, Chen J, Schnaas L, Hernández C, Osorio E, Portillo MG, López-Carrillo L. Maternal MTHFR 677C> T genotype and dietary intake of folate and vitamin

For peer review only - http://bmjopen.bmj.com/site/about/guidelines.xhtml

B12: their impact on child neurodevelopment. Nutritional neuroscience. 2009 Feb 1;12(1):13-20.

16. Siddiqua JT. Vitamin B12 deficiency in pregnancy and lactation: is there a need for pre and post-natal supplementation? J Nutr Disord Ther. 2014;4(2):1-8.
17. Dror DK, Allen LH. Effect of vitamin B12 deficiency on neurodevelopment in infants: current knowledge and possible mechanism. Nutr Rev. 2008; 66:250-255.
18. Bhate V, Deshpande S, Bhat D, Joshi N, Ladkat R, Watve S, et al. Vitamin B12 status of pregnant Indian women and cognitive function in their 9-year-old children. Food Nutr Bull. 2008; 29:249– 254.
19. Torsvik I, Ueland PM, Markestad T, Bjørke-Monsen AL. Cobalamin supplementation improves motor development and regurgitations in infants: results from a randomized intervention study. The American journal of clinical nutrition. 2013 Sep 11;98(5):1233-40.
20. Duggan C, Srinivasan K, Thomas T, Samuel T, Rajendran R, Muthayya S, Finkelstein JL, Lukose A, Fawzi W, Allen LH, Bosch RJ. Vitamin B-12 supplementation during pregnancy and early lactation increases maternal, breast milk, and infant measures of vitamin B-12 status. The Journal of nutrition. 2014 Mar 5;144(5):758-64.
21. Srinivasan K, Thomas T, Kapanee ARM, Ramthal A, Bellinger DC, Bosch RJ et al. Effects of maternal vitamin B12 supplementation on early infant neurocognitive outcomes: a randomised controlled clinical trial. Matern Child Nutr. 2016: doi:10.1111/mcn/12345.
22. Kvestad I, Taneja S, Kumar T, Hysing M, Refsum H, Yajnik CS, Bhandari N, Strand TA, Folate and Vitamin B12 Study Group. Vitamin B12 and folic acid improve gross motor and problem-solving skills in young North Indian children: a randomized placebo-controlled trial. Plos one. 2015 Jun 22;10(6):e0129915.
23. Siddiqua TJ, Ahmad SM, Ahsan SB, Rashid M, Roy A, Rahman SM et al. Vitamin B12 supplementation during pregnancy and postpartum improves B12 status of both mothers and infants but vaccine response in mothers only: a randomised control in Bangladesh. Eur J Nutr. 2016; 55(1)281-293.
24. Katre P, Bhat D, Lubree H, Otiv S, Joshi S, Joglekar C et al. Vitamin B12 and folic acid supplementation and plasma total homocysteine concentrations in pregnant women India women with low B12 and high folate status. Asia Pac J Clin Nutr. 2010;19(3):335-43.
25. Winje BA, Kvestad I, Krishnamachari S, Manji K, Taneja S, Bellinger DC, Bhandari N, Bisht S, Darling AM, Duggan CP, Fawzi W. Does early vitamin B12 supplementation improve neurodevelopment and cognitive function in childhood and into school age: a study protocol

For peer review only - http://bmjopen.bmj.com/site/about/guidelines.xhtml

for extended follow-ups from randomised controlled trials in India and Tanzania. BMJ open. 2018 Feb 1;8(2):e018962.

26. Strand TA, Ulak M, Chandyo RK, Kvestad I, Hysing M, Shrestha M, Basnet S, Ranjitkar S, Shrestha L, Shrestha PS. The effect of vitamin B 12 supplementation in Nepalese infants on growth and development: study protocol for a randomized controlled trial. Trials. 2017 Dec;18(1):187.
27. Nagpal J, Bhargava VL, Sengupta R, Dhall S, Agarwal N, Gulati K. A prospective cohort study to evaluate the utility of antenatal doppler velocimetry in prediction of foetal malnutrition, neonatal morbidity, mortality and neurodevelopmental outcome: Final report submitted to ICMR 2015. Downloaded from url: http://14.139.60.56/ICMRFINALREPORT/SQL_FR_ABS_PDF_Disp.aspx?Type=FR&Proj_No=2009-01550&SLNO=1
28. Bradley RH, Caldwell BM, Rock SL,Ramey CT,Barnard KE, Gray C et al. Home environment and cognitive development in the first 3 years of life: a collaborative study involving six sites and three ethnic groups in North America. Developmental Psychology. 1989; 25 (2),217.
29. Mearns GJ, Rush EC. Screening for inadequate dietary vitamin B12 intake in South Asian women using a nutrient-specific, semi-quantitative food frequency questionnaire. Asia Pacific journal of clinical nutrition. 2016 Nov 18.
30. Patni B. Developmental Assessment Scales for Indian Infants (DASII). Indian J Pract Pediatr. 2012;14(4):409–12.
31. World Health Organization Multicentre Growth Reference Study Group. World Health Organization Child Growth Standards based on length/height, weight and age. Acta paediatrica (Oslo, Norway: 1992). Supplement. 2006 Apr; 450:76.
32. R Core Team (2013). R: A language and environment for statistical computing. R Foundation for Statistical Computing, Vienna, Austria. Available via: //www.R-project.org.
33. ATLAS.Ti: The Qualitative Data Analysis; Research Software.2019.
34. Chan A-W, Tetzlaff JM, Altman DG, Laupacis A, Gøtzsche PC, Krleža-Jerić K, Hróbjartsson A, Mann H, Dickersin K, Berlin J, Doré C, Parulekar W, Summerskill W, Groves T, Schulz K, Sox H, Rockhold FW, Rennie D, Moher D. SPIRIT 2013 Statement: Defining standard protocol items for clinical trials. Ann Intern Med. 2013;158(3):200-207.
35. Yajnik CS, Behere RV, Bhat DS, Memane N, Raut D, Ladkat R, Yajnik PC, Kumaran K, Fall CH. A physiological dose of oral vitamin B-12 improves hematological, biochemical-metabolic indices and peripheral nerve function in B-12 deficient Indian adolescent women. PloS one. 2019;14(10).

For peer review only - http://bmjopen.bmj.com/site/about/guidelines.xhtml

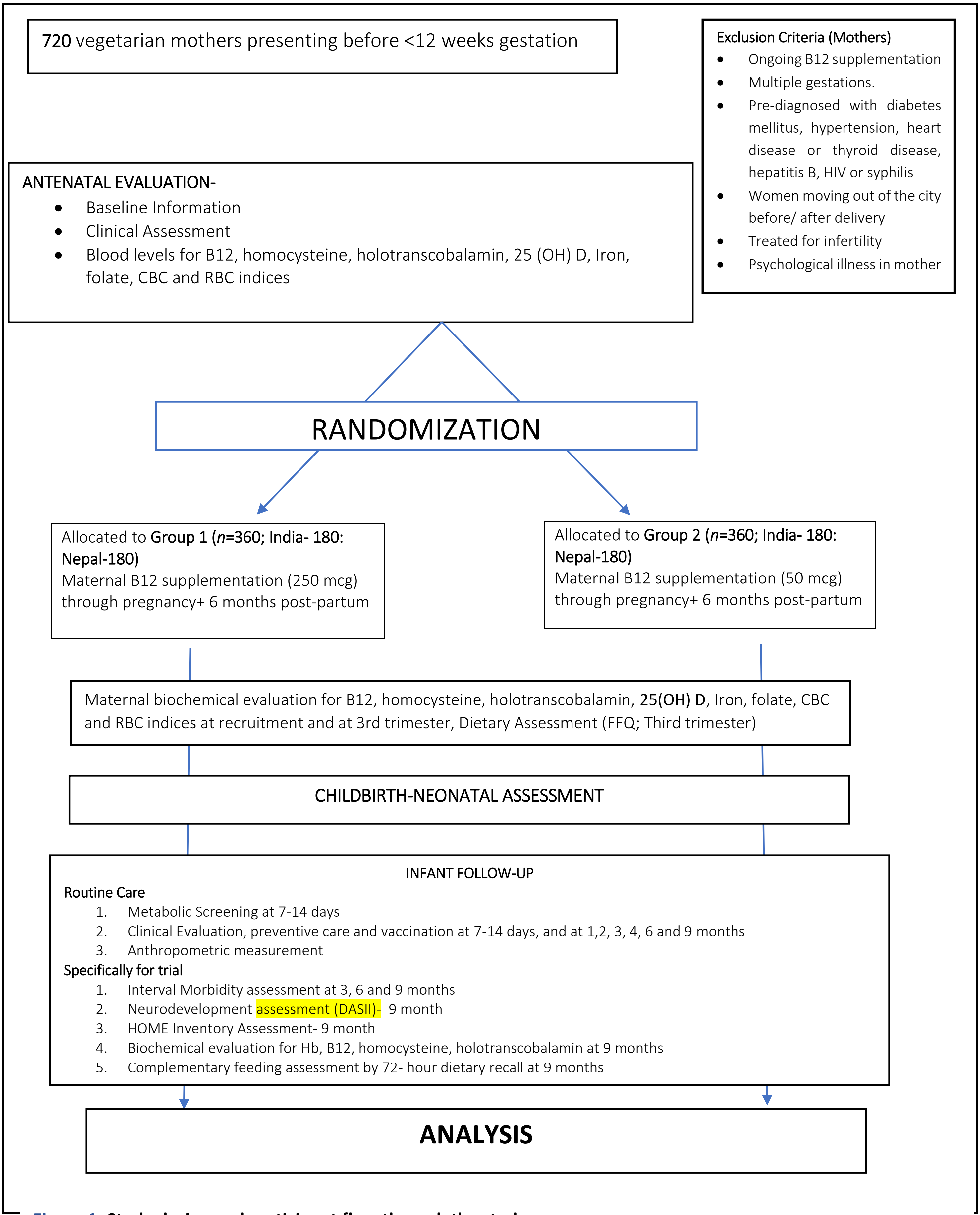

Figure 1. Study design and participant flow through the study.

For peer review only

Supplementary Material: Table 1. Visit schedule

**Study Plan**	**Initial Contact (<12 weeks gestation)**		**M1**	**M2-M9**	**M10**	**At Birth**	**I1**	**I2-I6**	**I7**
**Schedule**	**Screening**	**Recruitment**	**Antenatal Follow-up**	**Antenatal Follow-up (monthly until second trimester, fortnightly in the third trimester**	**Antenatal Follow-up (weekly after 36 weeks until delivery)**		**Infant 7-14 day**	**Infant follow-up: At months-1 (± 1 wk), 2 (± 2 wks), 3 (± 2 wks), 4 (± 2 wks) & 6 (± 2 wks)**	**Infant follow-up Age 9 months ± 14 days;**
First Screening Visit	X								
Maternal Inclusion/Exclusion criteria	X	X							
Informed consent of the participant		X							
Questionnaire Administration		X							
Randomization		X							
Supplementation		X	X	X	X	X	X	X	
Blood Sampling (1st trimester)		X							
Home Visit (if required)			X	X	X	X	X	X	X
Monitoring of the Compliance			X	X	X	X	X	X	X
Adverse events			X	X	X	X	X	X	X
Blood Sampling (3rd trimester, >26 weeks gestation)				X					
Mother Food Frequency Questionnaire (>26 weeks gestation)				X					
Infant Anthropometry (Weight/length/Head Circumference)						X	X	X	X
Monitoring for neonatal morbidity*						X	X	X	X
Application of Exit Criteria (specified earlier)			X	X	X	X	X	X	X
Metabolic Screening of infant							X		
Infant morbidity							X	X	X
Infant blood sampling									X
Complementary feeding 72 hr. Dietary Recall									X
DASII Scale									X

For peer review only

HOME Inventory									X

For peer review only

Reporting checklist for protocol of a clinical trial.

Based on the SPIRIT guidelines.

Instructions to authors

Complete this checklist by entering the page numbers from your manuscript where readers will find each of the items listed below.

Your article may not currently address all the items on the checklist. Please modify your text to include the missing information. If you are certain that an item does not apply, please write "n/a" and provide a short explanation.

Upload your completed checklist as an extra file when you submit to a journal.

In your methods section, say that you used the SPIRITreporting guidelines, and cite them as:

Chan A-W, Tetzlaff JM, Altman DG, Laupacis A, Gøtzsche PC, Krleža-Jerić K, Hróbjartsson A, Mann H, Dickersin K, Berlin J, Doré C, Parulekar W, Summerskill W, Groves T, Schulz K, Sox H, Rockhold FW, Rennie D, Moher D. SPIRIT 2013 Statement: Defining standard protocol items for clinical trials. Ann Intern Med. 2013;158(3):200-207

For peer review only

		Reporting Item	Page Number
**Administrative information**			
Title	#1	Descriptive title identifying the study design, population, interventions, and, if applicable, trial acronym	1

Trial registration	#2a	Trial identifier and registry name. If not yet registered, name of intended registry	6
Trial registration: data set	#2b	All items from the World Health Organization Trial Registration Data Set	6
Protocol version	#3	Date and version identifier	15
Funding	#4	Sources and types of financial, material, and other support	17
Roles and responsibilities: contributorship	#5a	Names, affiliations, and roles of protocol contributors	1
Roles and responsibilities: sponsor contact information	#5b	Name and contact information for the trial sponsor	17
Roles and responsibilities: sponsor and funder	#5c	Role of study sponsor and funders, if any, in study design; collection, management, analysis, and interpretation of data; writing of the report; and the decision to submit the report for publication, including whether they will have ultimate authority over any of these activities	17
Roles and responsibilities: committees	#5d	Composition, roles, and responsibilities of the coordinating centre, steering committee, endpoint adjudication committee, data management team, and other individuals or groups overseeing the trial, if applicable (see Item 21a for data monitoring committee)	

For peer review only

Introduction

Background and rationale	#6a	Description of research question and justification for undertaking the trial, including summary of relevant studies (published and unpublished) examining benefits and harms for each intervention	4-5
Background and rationale: choice of comparators	#6b	Explanation for choice of comparators	4-5
Objectives	#7	Specific objectives or hypotheses	5
Trial design	#8	Description of trial design including type of trial (eg, parallel group, crossover, factorial, single group), allocation ratio, and framework (eg, superiority, equivalence, non-inferiority, exploratory)	

Methods: Participants, interventions, and outcomes

Study setting	#9	Description of study settings (eg, community clinic, academic hospital) and list of countries where data will be collected. Reference to where list of study sites can be obtained	6
Eligibility criteria	#10	Inclusion and exclusion criteria for participants. If applicable, eligibility criteria for study centres and	8

For peer review only

		individuals who will perform the interventions (eg, surgeons, psychotherapists)	
Interventions: description	#11a	Interventions for each group with sufficient detail to allow replication, including how and when they will be administered	10-12
Interventions: modifications	#11b	Criteria for discontinuing or modifying allocated interventions for a given trial participant (eg, drug dose change in response to harms, participant request, or improving / worsening disease)	
Interventions: adherance	#11c	Strategies to improve adherence to intervention protocols, and any procedures for monitoring adherence (eg, drug tablet return; laboratory tests)	12
Interventions: concomitant care	#11d	Relevant concomitant care and interventions that are permitted or prohibited during the trial	11
Outcomes	#12	Primary, secondary, and other outcomes, including the specific measurement variable (eg, systolic blood pressure), analysis metric (eg, change from baseline, final value, time to event), method of aggregation (eg, median, proportion), and time point for each outcome. Explanation of the clinical relevance of chosen efficacy and harm outcomes is strongly recommended	12-13
Participant timeline	#13	Time schedule of enrolment, interventions (including any run-ins and washouts), assessments, and visits for participants. A schematic diagram is highly recommended	11

For peer review only

		(see Figure)	
Sample size	#14	Estimated number of participants needed to achieve study objectives and how it was determined, including clinical and statistical assumptions supporting any sample size calculations	13
Recruitment	#15	Strategies for achieving adequate participant enrolment to reach target sample size	
**Methods: Assignment of interventions (for controlled trials)**			
Allocation: sequence generation	#16a	Method of generating the allocation sequence (eg, computer-generated random numbers), and list of any factors for stratification. To reduce predictability of a random sequence, details of any planned restriction (eg, blocking) should be provided in a separate document that is unavailable to those who enrol participants or assign interventions	9
Allocation concealment mechanism	#16b	Mechanism of implementing the allocation sequence (eg, central telephone; sequentially numbered, opaque, sealed envelopes), describing any steps to conceal the sequence until interventions are assigned	9
Allocation: implementation	#16c	Who will generate the allocation sequence, who will enrol participants, and who will assign participants to interventions	9

For peer review only

Blinding (masking)	#17a	Who will be blinded after assignment to interventions (eg, trial participants, care providers, outcome assessors, data analysts), and how	9
Blinding (masking): emergency unblinding	#17b	If blinded, circumstances under which unblinding is permissible, and procedure for revealing a participant's allocated intervention during the trial	
**Methods: Data collection, management, and analysis**			
Data collection plan	#18a	Plans for assessment and collection of outcome, baseline, and other trial data, including any related processes to promote data quality (eg, duplicate measurements, training of assessors) and a description of study instruments (eg, questionnaires, laboratory tests) along with their reliability and validity, if known. Reference to where data collection forms can be found, if not in the protocol	10-12
Data collection plan: retention	#18b	Plans to promote participant retention and complete follow-up, including list of any outcome data to be collected for participants who discontinue or deviate from intervention protocols	12
Data management	#19	Plans for data entry, coding, security, and storage, including any related processes to promote data quality (eg, double data entry; range checks for data values).	14

For peer review only

		Reference to where details of data management procedures can be found, if not in the protocol	
Statistics: outcomes	#20a	Statistical methods for analysing primary and secondary outcomes. Reference to where other details of the statistical analysis plan can be found, if not in the protocol	13-14
Statistics: additional analyses	#20b	Methods for any additional analyses (eg, subgroup and adjusted analyses)	13-14
Statistics: analysis population and missing data	#20c	Definition of analysis population relating to protocol non-adherence (eg, as randomised analysis), and any statistical methods to handle missing data (eg, multiple imputation)	
**Methods: Monitoring**			
Data monitoring: formal committee	#21a	Composition of data monitoring committee (DMC); summary of its role and reporting structure; statement of whether it is independent from the sponsor and competing interests; and reference to where further details about its charter can be found, if not in the protocol. Alternatively, an explanation of why a DMC is not needed	
Data monitoring: interim analysis	#21b	Description of any interim analyses and stopping guidelines, including who will have access to these interim results and make the final decision to terminate the trial	
Harms	#22	Plans for collecting, assessing, reporting, and managing solicited and spontaneously reported adverse events and other unintended effects of trial interventions or trial	

For peer review only

		conduct	
Auditing	#23	Frequency and procedures for auditing trial conduct, if any, and whether the process will be independent from investigators and the sponsor	
**Ethics and dissemination**			
Research ethics approval	#24	Plans for seeking research ethics committee / institutional review board (REC / IRB) approval	15
Protocol amendments	#25	Plans for communicating important protocol modifications (eg, changes to eligibility criteria, outcomes, analyses) to relevant parties (eg, investigators, REC / IRBs, trial participants, trial registries, journals, regulators)	15
Consent or assent	#26a	Who will obtain informed consent or assent from potential trial participants or authorised surrogates, and how (see Item 32)	15
Consent or assent: ancillary studies	#26b	Additional consent provisions for collection and use of participant data and biological specimens in ancillary studies, if applicable	
Confidentiality	#27	How personal information about potential and enrolled participants will be collected, shared, and maintained in order to protect confidentiality before, during, and after the trial	15
Declaration of	#28	Financial and other competing interests for principal	17

For peer review only

interests		investigators for the overall trial and each study site	
Data access	#29	Statement of who will have access to the final trial dataset, and disclosure of contractual agreements that limit such access for investigators	17
Ancillary and post-trial care	#30	Provisions, if any, for ancillary and post-trial care, and for compensation to those who suffer harm from trial participation	
Dissemination policy: trial results	#31a	Plans for investigators and sponsor to communicate trial results to participants, healthcare professionals, the public, and other relevant groups (eg, via publication, reporting in results databases, or other data sharing arrangements), including any publication restrictions	14
Dissemination policy: authorship	#31b	Authorship eligibility guidelines and any intended use of professional writers	
Dissemination policy: reproducible research	#31c	Plans, if any, for granting public access to the full protocol, participant-level dataset, and statistical code	14
**Appendices**			
Informed consent materials	#32	Model consent form and other related documentation given to participants and authorised surrogates	
Biological specimens	#33	Plans for collection, laboratory evaluation, and storage of biological specimens for genetic or molecular analysis in the current trial and for future use in ancillary studies, if applicable	

For peer review only

None The SPIRIT checklist is distributed under the terms of the Creative Commons Attribution License CC-BY-ND 3.0. This checklist can be completed online using https://www.goodreports.org/, a tool made by the EQUATOR Network in collaboration with Penelope.ai

For peer review only
